# ATM-dependent DNA damage response constrains cell growth and drives clonal hematopoiesis in telomere biology disorders

**DOI:** 10.1172/JCI181659

**Published:** 2025-04-03

**Authors:** Christopher M. Sande, Stone Chen, Dana V. Mitchell, Ping Lin, Diana M. Abraham, Jessie Minxuan Cheng, Talia Gebhard, Rujul J. Deolikar, Colby Freeman, Mary Zhou, Sushant Kumar, Michael Bowman, Robert L. Bowman, Shannon Zheng, Bolormaa Munkhbileg, Qijun Chen, Natasha L. Stanley, Kathy Guo, Ajibike Lapite, Ryan Hausler, Deanne M. Taylor, James Corines, Jennifer J.D. Morrissette, David B. Lieberman, Guang Yang, Olga Shestova, Saar Gill, Jiayin Zheng, Kelcy Smith-Simmer, Lauren G. Banaszak, Kyle N. Shoger, Erica F. Reinig, Madilynn Peterson, Peter Nicholas, Amanda J. Walne, Inderjeet Dokal, Justin P. Rosenheck, Karolyn A. Oetjen, Daniel C. Link, Andrew E. Gelman, Christopher R. Reilly, Ritika Dutta, R. Coleman Lindsley, Karyn J. Brundige, Suneet Agarwal, Alison A. Bertuch, Jane E. Churpek, Laneshia K. Tague, F. Brad Johnson, Timothy S. Olson, Daria V. Babushok

**Affiliations:** 1 Department of Pathology and Laboratory Medicine, Hospital of the University of Pennsylvania, Philadelphia, Pennsylvania, USA.; 2 Department of Laboratories, Seattle Children’s Hospital, Seattle, Washington, USA.; 3 Department of Laboratory Medicine and Pathology, University of Washington, Seattle, Washington, USA.; 4 Division of Hematology-Oncology, Department of Medicine, Hospital of the University of Pennsylvania, Philadelphia, Pennsylvania, USA.; 5 Department of Biomedical and Health Informatics, Children’s Hospital of Philadelphia, Philadelphia, Pennsylvania, USA.; 6 Comprehensive Bone Marrow Failure Center, Children’s Hospital of Philadelphia, Philadelphia, Pennsylvania, USA.; 7 Drexel University College of Medicine, Drexel University, Philadelphia, Pennsylvania, USA.; 8 Department of Cancer Biology, University of Pennsylvania, Philadelphia, Pennsylvania, USA.; 9 Department of Pediatrics, Children’s Hospital of Pennsylvania, Philadelphia, Pennsylvania, USA.; 10 Department of Pediatrics, Division of Hematology/Oncology, Baylor College of Medicine, Houston, Texas, USA.; 11 Department of Pediatrics, University of Pennsylvania Perelman School of Medicine, Philadelphia, Pennsylvania, USA.; 12 Department of Biostatistics, Epidemiology, and Informatics, University of Pennsylvania Perelman School of Medicine, Children’s Hospital of Philadelphia, Philadelphia, Pennsylvania, USA.; 13 Division of Hematology, Medical Oncology, and Palliative Care, Department of Medicine and; 14 Department of Pathology and Laboratory Medicine, University of Wisconsin-Madison, Madison, Wisconsin, USA.; 15 Division of Oncology, Department of Pediatrics, Children’s Hospital of Philadelphia, Philadelphia, Pennsylvania, USA.; 16 Blizard Institute Faculty of Medicine and Dentistry, Queen Mary University of London, London, United Kingdom.; 17 Division of Pulmonary, Allergy, Critical Care and Sleep Medicine, Department of Medicine, Ohio State University, Columbus, Ohio, USA.; 18 Division of Oncology, Section of Stem Cell Biology, Department of Medicine,; 19 Department of Pathology & Immunology, and; 20 Department of Surgery, Division of Cardiothoracic Surgery, Washington University in St. Louis, St. Louis, Missouri, USA.; 21 Division of Hematological Malignancies, Department of Medical Oncology, Dana-Farber Cancer Institute, Boston, Massachusetts, USA.; 22 Division of Hematology/Oncology, Boston Children’s Hospital, Pediatric Oncology, Dana-Farber Cancer Institute, Department of Pediatrics, Harvard Medical School, Boston, Massachusetts, USA.; 23 Texas Children’s Cancer and Hematology Centers, Houston, Texas, USA.; 24 Division of Pulmonary & Critical Care Medicine, Department of Medicine, Washington University in St. Louis, St. Louis, Missouri, USA.

**Keywords:** Hematology, Oncology, Clonal selection, Hematopoietic stem cells, Telomeres

## Abstract

Telomere biology disorders (TBDs) are genetic diseases caused by defective telomere maintenance. TBD patients often develop bone marrow failure and have an increased risk of myeloid neoplasms. To better understand the factors underlying hematopoietic outcomes in TBD, we comprehensively evaluated acquired genetic alterations in hematopoietic cells from 166 pediatric and adult TBD patients. Of these patients, 47.6% (28.8% of children, 56.1% of adults) had clonal hematopoiesis. Recurrent somatic alterations involved telomere maintenance genes (7.6%), spliceosome genes (10.4%, mainly *U2AF1* p.S34), and chromosomal alterations (20.2%), including 1q gain (5.9%). Somatic variants affecting the DNA damage response (DDR) were identified in 21.5% of patients, including 20 presumed loss-of-function variants in ataxia-telangiectasia mutated (*ATM*). Using multimodal approaches, including single-cell sequencing, assays of ATM activation, telomere dysfunction-induced foci analysis, and cell-growth assays, we demonstrate telomere dysfunction–induced activation of the ATM-dependent DDR pathway with increased senescence and apoptosis in TBD patient cells. Pharmacologic ATM inhibition, modeling the effects of somatic *ATM* variants, selectively improved TBD cell fitness by allowing cells to bypass DDR-mediated senescence without detectably inducing chromosomal instability. Our results indicate that ATM-dependent DDR induced by telomere dysfunction is a key contributor to TBD pathogenesis and suggest dampening hyperactive ATM-dependent DDR as a potential therapeutic intervention.

## Abstract 2

A multicenter study of 166 patients with telomere biology disorders identified frequent somatic mutations in patients’ hematopoietic cells, uncovering a previously unknown mechanism of somatic rescue.

## Graphical abstract

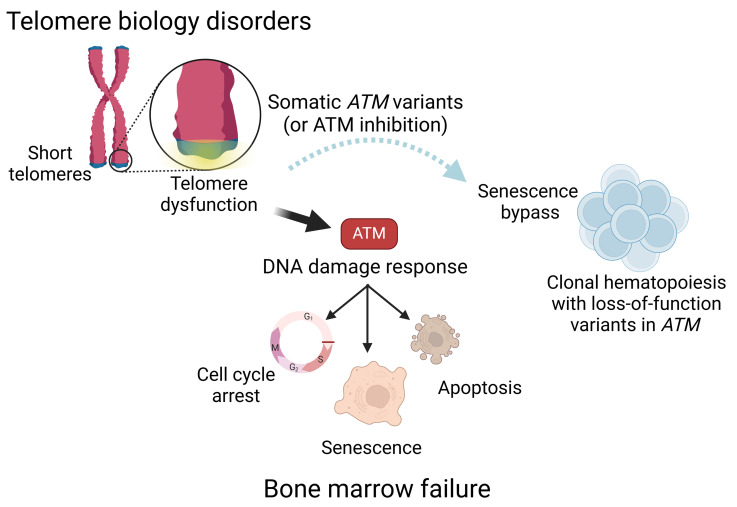

## Introduction

Telomere biology disorders (TBDs) are a heterogeneous group of diseases caused by genetic defects in telomere maintenance (1). In TBD patients, the resultant telomere shortening leads to a multisystem disease, manifesting as a variety of conditions that may include cytopenias, bone marrow failure (BMF), mucocutaneous abnormalities, interstitial lung disease (ILD), cirrhosis, hepatopulmonary syndrome, and cancer (2). Notably, TBD patients have an increased risk of myeloid malignancies and are estimated to have a greater than 20-fold higher risk of acute myeloid leukemia (AML) and a greater than 150-fold higher risk of myelodysplastic syndrome (MDS) compared with the general population (3–5). Emerging data show that occult TBD underlies a subset of apparently sporadic MDS cases, where TBD is predictive of inferior survival and increased nonrelapse mortality (6). Despite the greatly increased relative risk of MDS/AML, only 8%–10% (3–5) of TBD patients develop myeloid neoplasms, and the ability to predict an individual patient’s risk of MDS/AML remains limited.

Clonal hematopoiesis (CH) occurs when hematopoietic stem cells or self-renewing hematopoietic progenitor cells acquire postzygotic genetic or epigenetic variants that give them a competitive advantage, leading to their clonal expansion. CH can occur without apparent hematologic abnormalities — a phenomenon known as CH of indeterminate potential (CHIP) — or it can occur in patients with cytopenias, termed clonal cytopenia of undetermined significance (CCUS). Both CHIP and CCUS may progress to overt myeloid malignancies. With the growing appreciation of the role of CH in the development of hematologic malignancies, several studies have explored CH in TBD (5, 7–13). While most TBD patients have evidence of clonality (7), somatic variants characteristic of age-related CH and MDS/AML in the general population are relatively infrequent (one notable exception being variants in *U2AF1*) (5, 7, 9, 14). Instead, somatic alterations leading to the reversion of the germline TBD gene variants and those leading to compensatory telomere elongation are recurrently seen in TBD (4, 10, 11, 13) and may provide a partial “rescue” of the short telomere phenotype, protecting against malignant transformation (4, 10, 11).

Mammalian telomeres consist of double-stranded TTAGGG repeats that terminate in a single-stranded 3′ overhang (15). This 3′ overhang region is bound by POT1 and hidden with a large duplex loop structure called the t-loop, which prevents single-stranded telomere ends from being recognized as double-strand breaks (15–17). When telomeres fail to form the t-loop or are critically shortened, a DNA damage response (DDR) is triggered (18–21). Activation of the ataxia-telangiectasia mutated (ATM) and ataxia-telangiectasia and Rad3-related (ATR) protein kinases is central to the DDR, setting off a cascade of events culminating in cell-cycle arrest and p53-dependent senescence or apoptosis (22–25). We hypothesized that telomere dysfunction–induced DDR contributes to BMF in TBD and selects for the emergence of DDR gene variants that allow cells to bypass these barriers to proliferation and survival.

To test this hypothesis, we comprehensively analyzed CH in a multi-institutional cohort of 166 TBD patients using a combination of methods, including cytogenetics, whole-exome sequencing (WES), and panel-based next-generation sequencing (NGS) focusing on DDR, senescence, and telomere maintenance pathways. We found several recurrent, somatic alterations in TBD, most notably targeting the DDR, with recurrent loss-of-function variants in *ATM*. We found that cells of TBD patients have upregulated ATM-dependent DDR and grow poorly, while ATM inhibition selectively improves TBD cell growth, suggesting a possible therapeutic strategy.

## Results

### Clinical characteristics of TBD patients.

**Figure 1:**
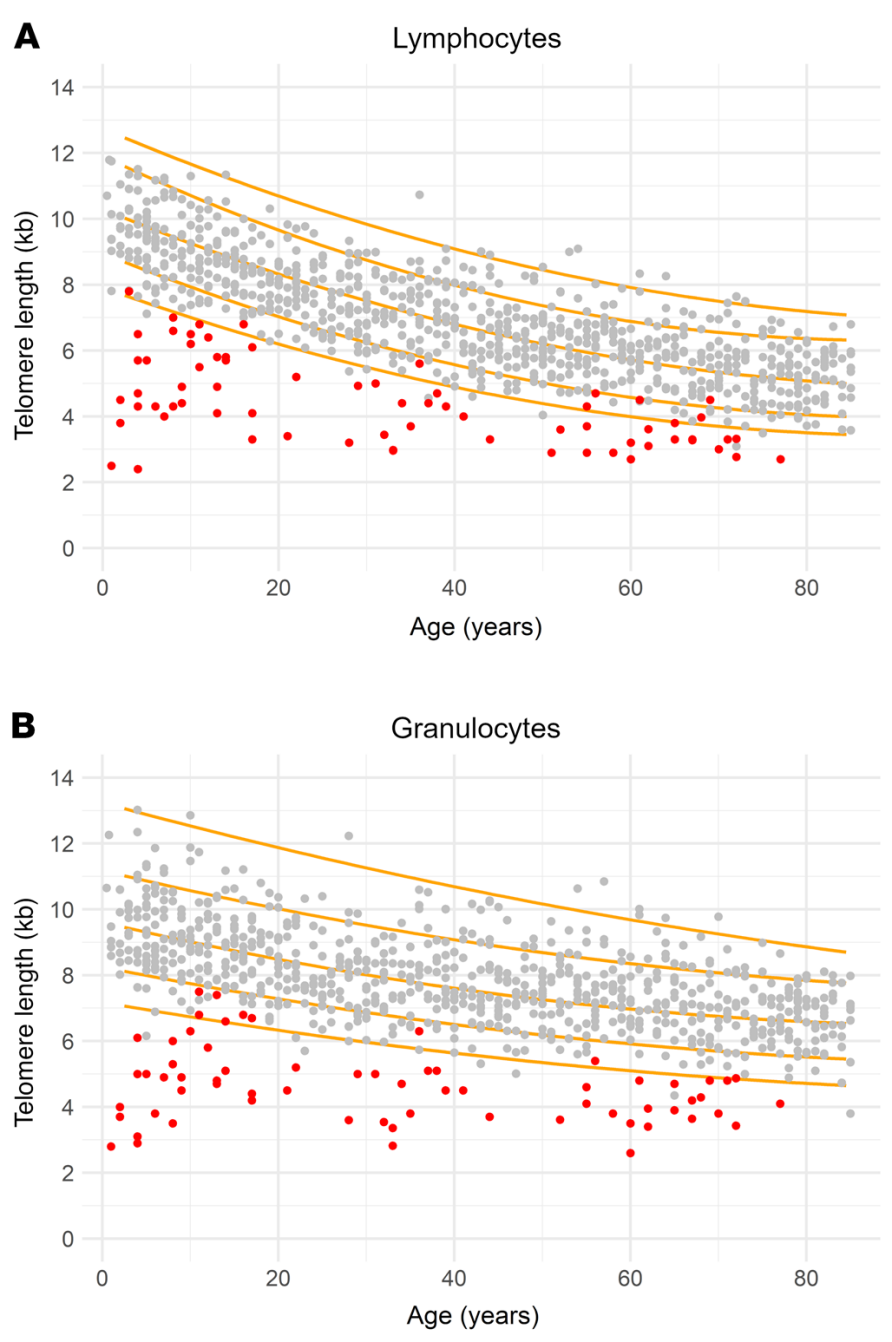
Telomere lengths of the patient cohort. Nomograms of average telomere lengths in (**A**) lymphocytes and (**B**) granulocytes obtained by flow-FISH for 70 patients in our cohort with available raw clinical telomere length measurement data (red) were plotted alongside published telomere length from healthy controls of different ages from the Johns Hopkins (*n* = 192) and Vancouver (*n* = 444) groups (81, 82), alongside the percentile curves.

Overall, 166 TBD patients from 146 families were recruited from 4 pediatric and 6 adult centers (Table 1). Patients had a median age of 41 years (range 1–78), and 140 (98.6% of evaluable patients) had lymphocyte telomere lengths less than the tenth percentile, with 110 (77.5%) having telomere lengths of less than the first percentile (Figure 1). Germline variants in TBD-associated genes were identified in 144 patients (86.7%), most commonly in *TERC* (*n* = 49, 29.5%), *TERT* (*n* = 39, 23.5%), *RTEL1* (*n* = 34, 20.5%), and *DKC1* (*n* = 13, 7.8%). Of these variants, 30% were considered to be of uncertain significance. Twenty-two patients (13.3%) had no germline variants identified in TBD-associated genes.

**Figure 2:**
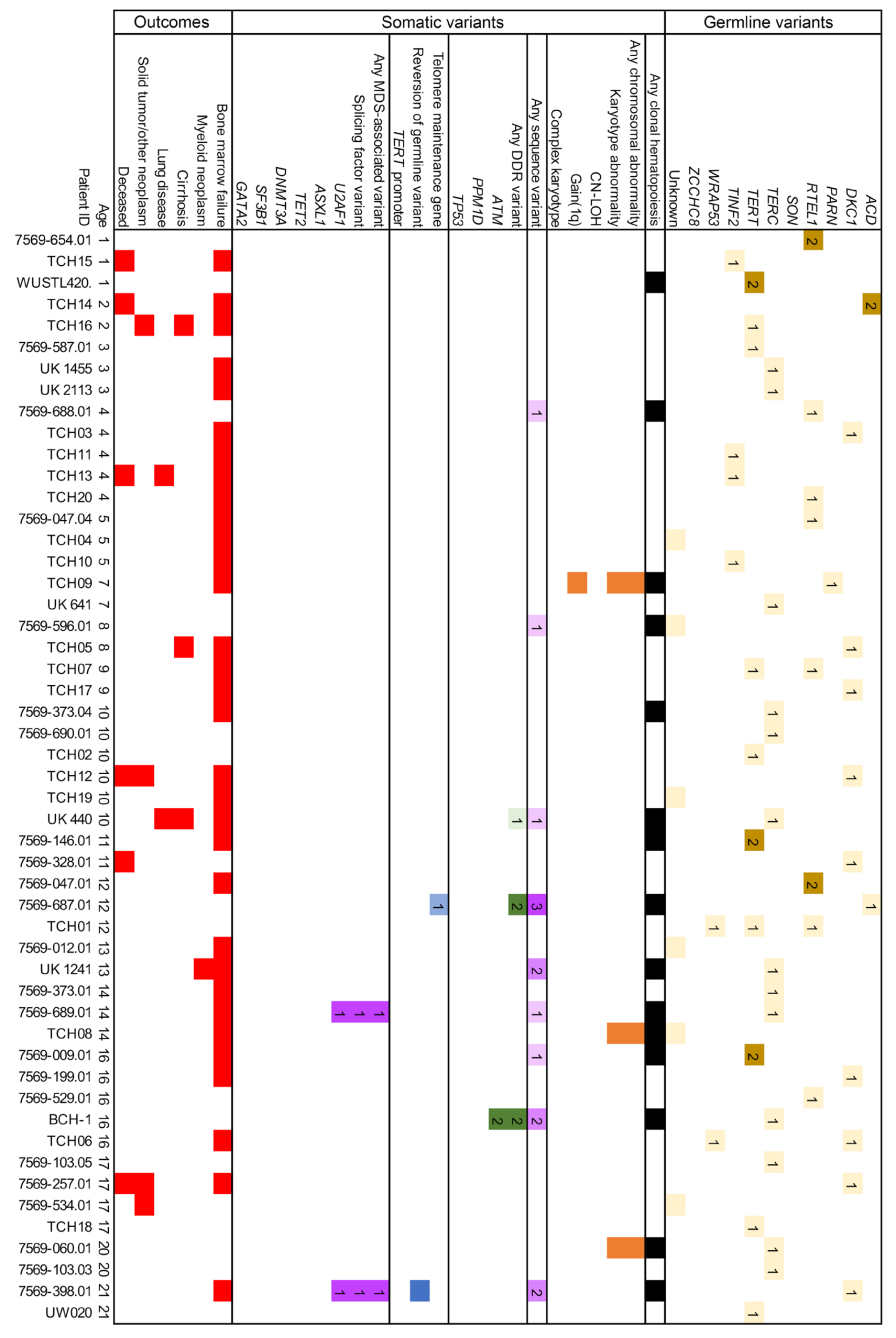
Summary of CH findings in pediatric TBD patients. Each column represents an individual patient, arranged horizontally by age (years) at the time of testing. Germline variants are detailed in the top section, CH findings in the middle section, and clinical complications in the bottom section. In each cell, the numbers indicate the numbers of unique variants in each subcategory, reflected by color shading of the following categories: germline TBD variants (yellow); somatic telomere-associated gene variants (blue); cytogenetic variants (orange); DDR-related sequence variants (green); and MDS/AML-related sequence variants (purple); all somatic variants (black); and major TBD complications (red).

**Figure 3:**
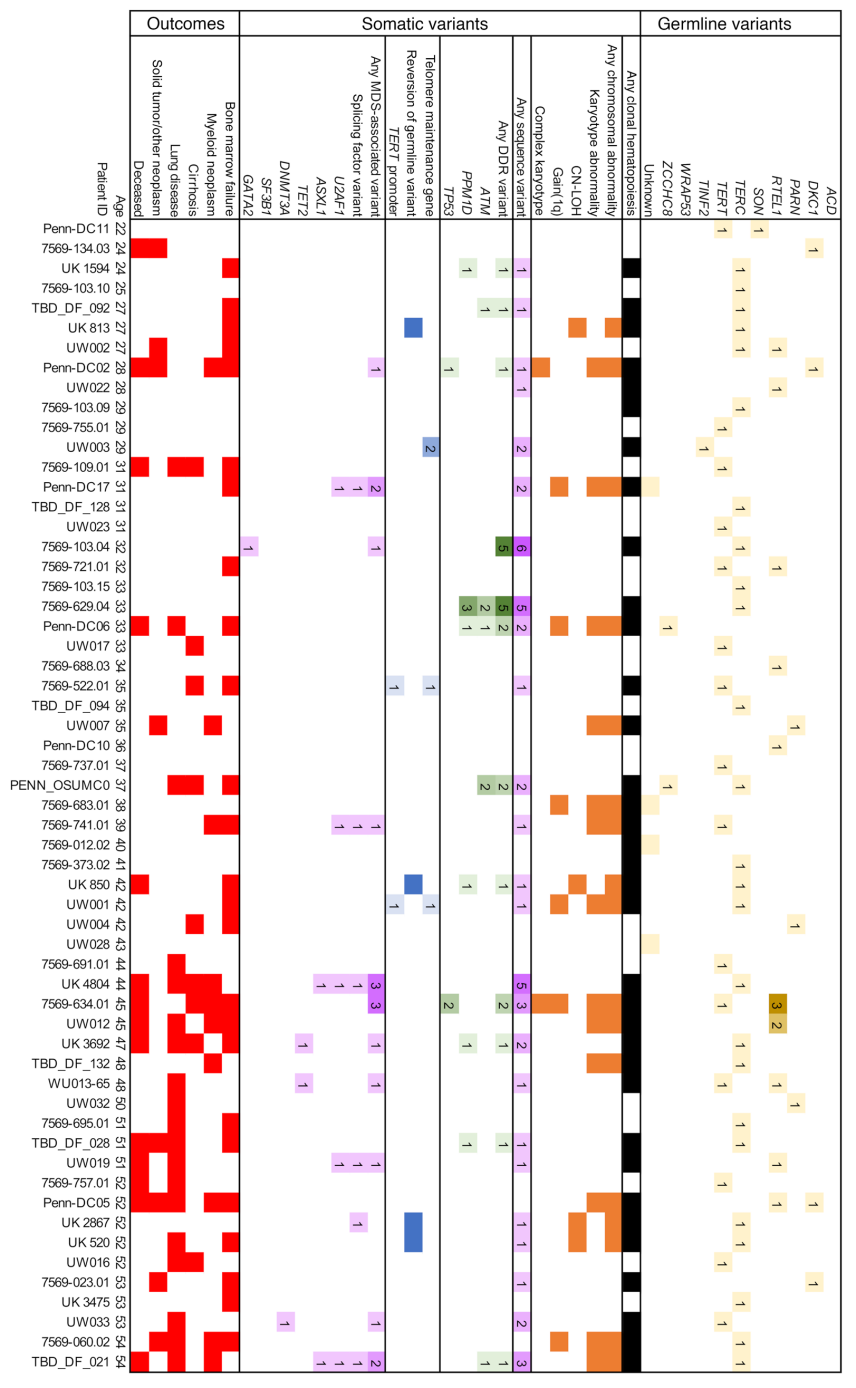
Summary of CH findings in adult TBD patients, ages 22–54. Each column represents an individual patient, arranged horizontally by age (years) at the time of testing. Germline variants are detailed in the top section, CH findings in the middle section, and clinical complications in the bottom section. In each cell, the numbers indicate the numbers of unique variants in each subcategory reflected by color shading of the following categories: germline TBD variants (yellow); somatic telomere-associated gene variants (blue); cytogenetic variants (orange); DDR-related sequence variants (green); and MDS/AML-related sequence variants (purple); all somatic variants (black); and major TBD complications (red).

**Figure 4:**
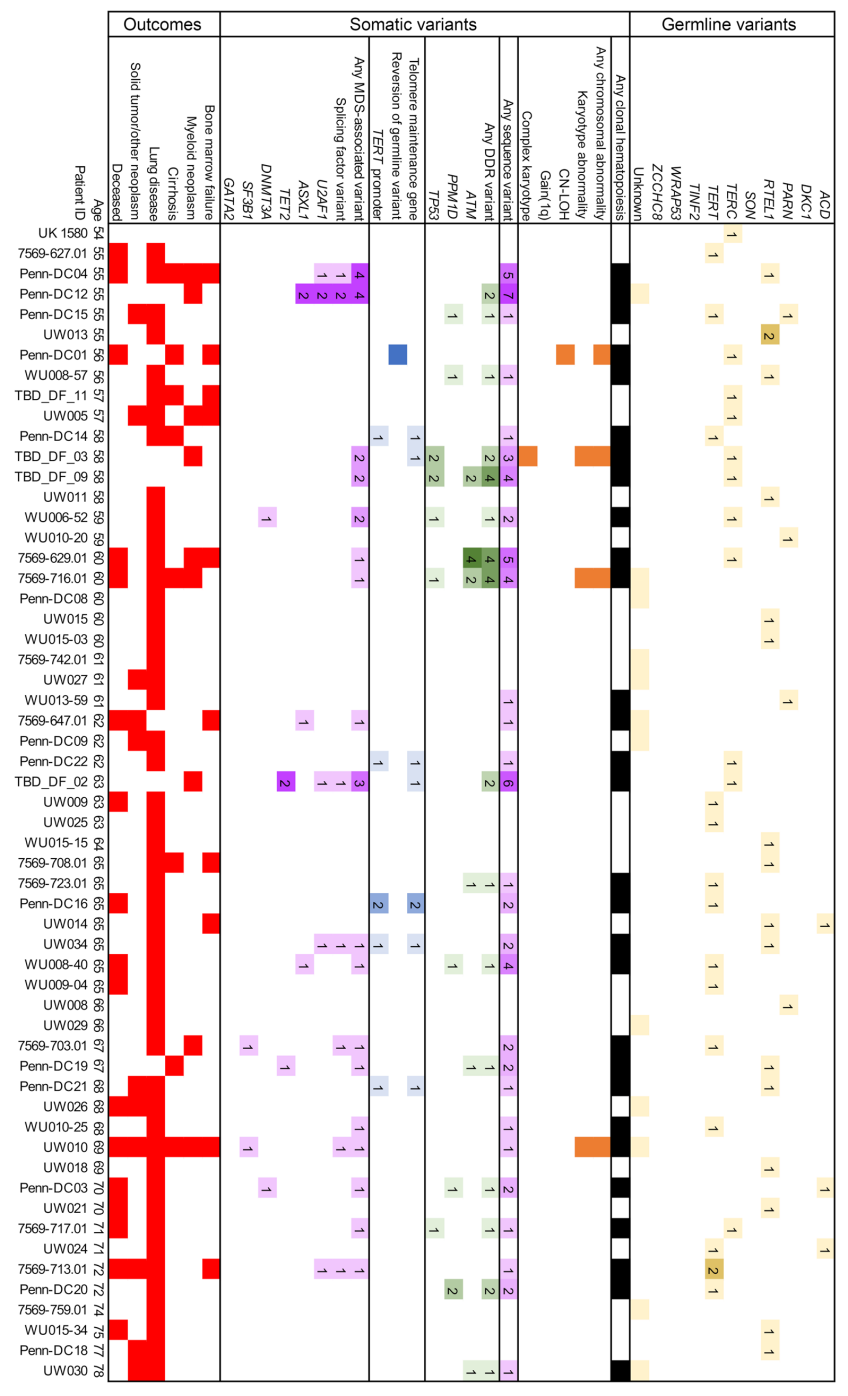
Summary of CH findings in adult TBD patients, ages 54–78. Each column represents an individual patient, arranged horizontally by age (years) at the time of testing. Germline variants are detailed in the top section, CH findings in the middle section, and clinical complications in the bottom section. In each cell, the numbers indicate the numbers of unique variants in each subcategory, reflected by colored shading of the following categories: germline TBD variants (yellow); somatic telomere-associated gene variants (blue); cytogenetic variants (orange); DDR-related sequence variants (green); and MDS/AML-related sequence variants (purple); all somatic variants (black); and major TBD complications (red).

**Figure 5:**
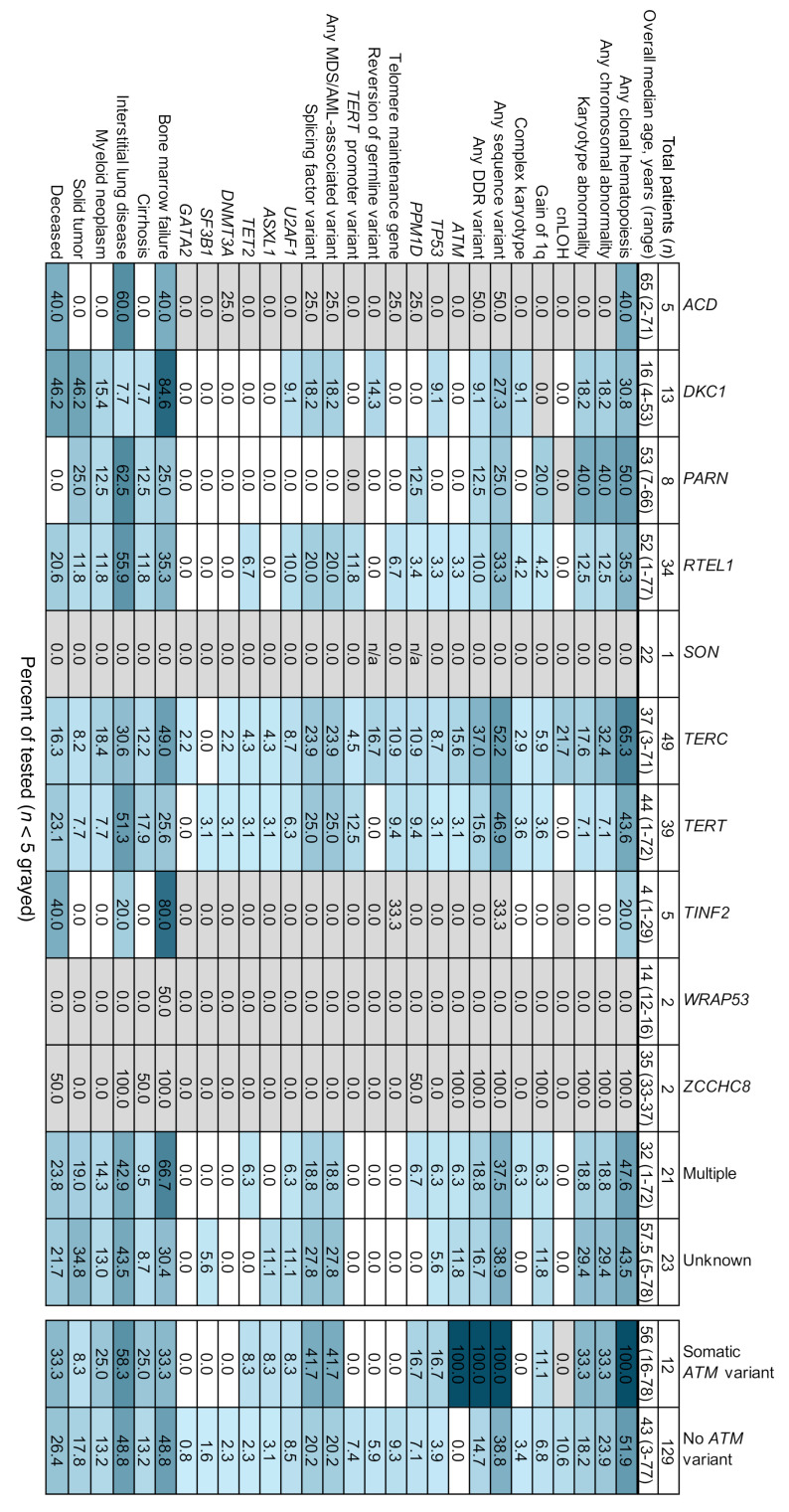
Cooccurrence of genetic and clinical outcomes by genotype. A cooccurrence plot with heat map indicating the percentage of tested patients of each genotype demonstrating the specified somatic abnormality or adverse TBD outcome. Patients with multiple germline variants were included both in the individual gene groups and in the multiple variant group. Entries where fewer than 5 total patients were evaluated have been shaded in light gray. A comparison of patients with and without somatic *ATM* variants is present in the final 2 columns. Because all patients with somatic *ATM* variants had CH and DDR variants, these categories are shown in dark blue. The corresponding numbers of patients with indicated findings (the numerator) and total number of patients evaluated per category (the denominator) are provided in Supplemental Figure 3.

A family history of either a TBD diagnosis or conditions attributable to TBD was seen in 66.3% of patients, and 74.7% of patients had major TBD-related end-organ complications (e.g., BMF, ILD, cirrhosis) (Table 1 and Supplemental Tables 1 and 2; supplemental material available online with this article; https://doi.org/10.1172/JCI181659DS1). Clinical manifestations correlated with TBD genetic subtype and age (Figure 2, Figure 3, Figure 4, and Figure 5). In younger patients, most of whom had *DKC1* and *TERC* variants in our cohort, complications were typically gastrointestinal or genitourinary tract strictures and BMF. Older patients, many of whom had *TERT* and *RTEL1* variants, were frequently affected by ILD or cirrhosis. Solid tumors occurred in 12.7% of our cohort, including patients with cutaneous squamous and/or basal cell carcinomas (*n* = 5); melanoma (*n* = 1); oral (*n* = 5), cervical (*n* = 1), anal (*n* = 1), and pulmonary (*n* = 1) squamous cell carcinomas; pulmonary small cell carcinoma (*n* = 1), gastrointestinal adenocarcinomas (*n* = 5), hepatic angiosarcomas (*n* = 2), and astrocytoma (*n* = 1) (Supplemental Table 1). Of the 21 patients with solid tumors, 6 (28.6%) developed these tumors after solid organ or bone marrow transplant. Solid tumor rates were similar between sexes, both when excluding 12 *DKC1*-mutated males with X-linked TBD (8 of 102 males [7.8%] versus 7 of 52 females [13.5%], *P* = 0.27) and when evaluating the complete cohort (14 of 114 males [12.3%], *P* = 0.83). One patient developed posttransplant lymphoproliferative disorder after a lung transplant, and monoclonal gammopathy of undetermined significance was diagnosed in 1 patient after a lung transplant. Twenty patients (12.0%) with a median age 54 years (ages 13–69) were diagnosed with myeloid neoplasms (including 1 with chronic myeloid leukemia, 1 with acute promyelocytic leukemia, and 3 others with confirmed AML progression).

### CH is common in TBD at all ages.

**Figure 6:**
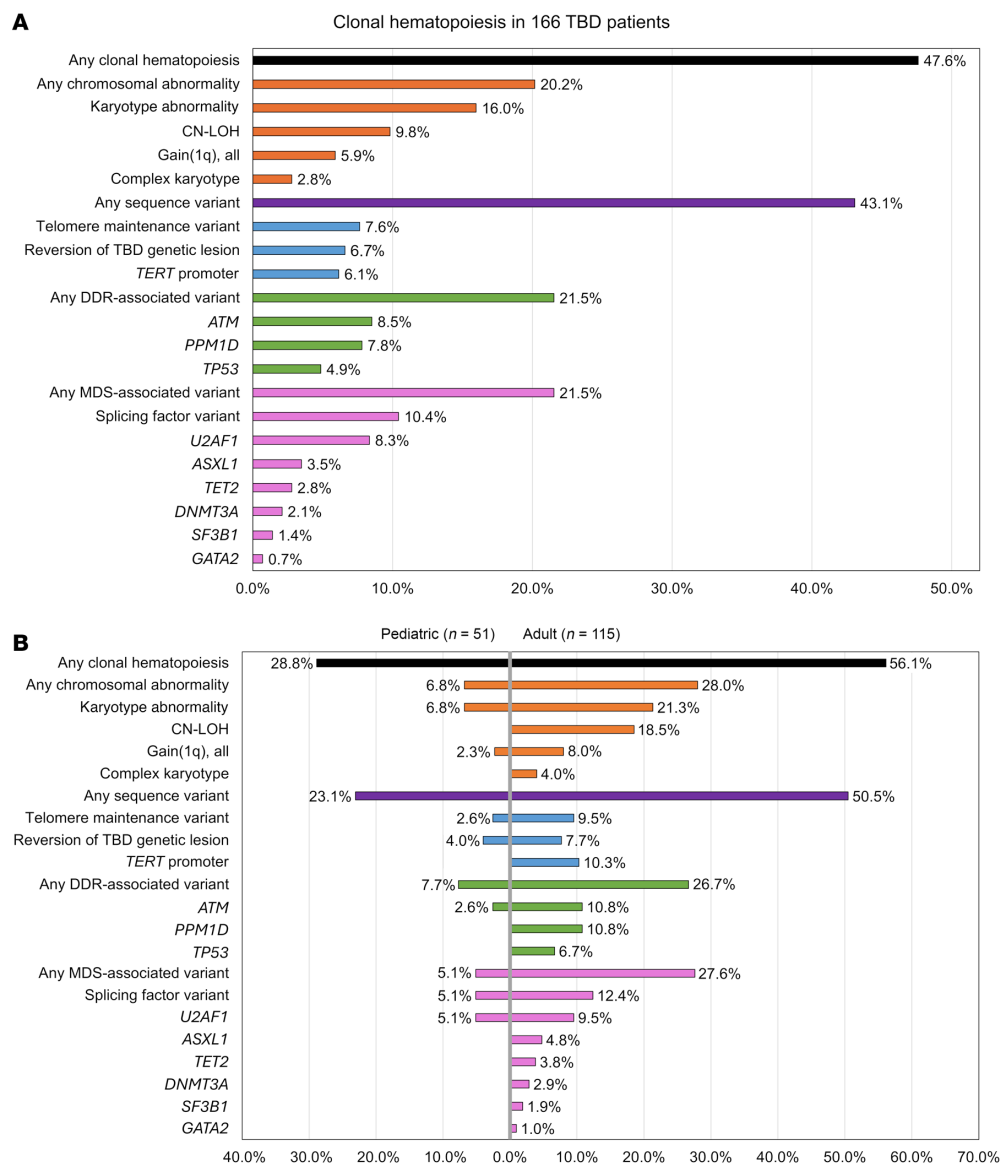
Summary of CH findings by category and compared by age. (**A**) Bar graph showing summary-level data for somatic findings with percentages including only those patients tested for the specific abnormality. (**B**) Comparative statistics for pediatric and adult patients. Pediatric patients include those 21 years of age and younger.

CH, defined as somatic genetic alterations or skewed X chromosomal inactivation in hematopoietic cells, as measured by any available modality, was identified in 79 of 166 (47.6%) patients (Figure 6, A and B, and Supplemental Table 3). Somatic variants included structural chromosomal abnormalities in 19 of 119 (16.0%) and copy neutral loss of heterozygosity (CN-LOH) in 5 of 51 (9.8%) cytogenomic arrays. NGS-identified sequence variants were detected in 62 of 144 (43.1%) patients. Clonality measured by skewed X chromosomal inactivation was identified in 8 of 9 (88.9%) patients analyzed by HUMARA assays. In pediatric patients, CH was less frequent than in adults (15 of 52 [28.8%] pediatric versus 64 of 114 [56.1%] in adults, *P* = 0.001), with fewer chromosomal abnormalities (3 of 44 [6.8%] versus 21 of 75 [28.0%], *P* = 0.005) and sequence variants (9 of 39 [23.1%] versus 53 of 105 [50.5%], *P* = 0.003) (Figure 6B).

Among cytogenetic variants, 7 of 119 (5.9%) patients had gain of chromosome arm 1q, of whom 6 experienced BMF. The gain of 1q was observed as a translocation in 6 of 7 cases (with partners including 7p, 16q, 19p, 21p, 21q, and Yq) and in 1 case was observed transiently as a component of a complex karyotype. Two of these patients had MDS, one of whom died from AML. Complex karyotype developed in 3 of 108 patients and only in the setting of *TP53*-mutated myeloid neoplasia (including the patient with gain of 1q). No other recurrent cytogenetic variants were identified, although several disease-defining cytogenetic abnormalities (26) were seen: del(5q), t(9;22), and t(15;17).

### Somatic variants in telomere maintenance genes.

Somatic reversion of germline TBD variants or somatic variants in telomere maintenance genes were identified in 17 patients. Six patients (6.7%, ages 21–60) had evidence of somatic reversion of their germline lesion (1 by point mutation in *DKC1*[7] and 5 by chromosome arm 3q CN-LOH containing the germline *TERC* variants). Somatic variants in telomere maintenance genes were identified in 7.6% of patients (11 of 144 evaluable patients, ages 13–70). Seven patients had acquired gain-of-function variants in the *TERT* promoter, of whom 3 had germline *TERT*, 2 germline *TERC*, and 2 germline *RTEL1* variants. One patient with a germline *TINF2* variant had 2 somatic missense variants in the OB1 domain of *POT1*. Three patients had somatic variants in other genes associated with telomere maintenance, including *RTEL1* and *CTCF* (Supplemental Table 3 and Supplemental Figure 1). Of the 17 patients with reversions or telomere maintenance variants, only the 2 with *CTCF* variants (ages 58 and 70) had a myeloid neoplasm at the time of evaluation. The remaining 15 had no evidence of a myeloid neoplasm at the time of evaluation (median age 52 years, range 12–68), 2 of whom with somatic reversion were followed longitudinally (1 from age 21 to 33, the other from age 54 to 60) with no adverse evolution to date.

### DDR and hematologic malignancy-associated gene variants.

Somatic variants in DDR pathway genes were seen in 21.5% (31 of 144) of evaluable patients, most commonly disrupting *ATM* (20 variants in 12 of 141 evaluable patients, 8.5%), which was also the most commonly mutated gene in our cohort (Figure 6). *PPM1D* variants were identified in 7.8% (13 variants in 11 of 141 evaluable patients), were exclusively seen at low levels (≤6% variant allele fraction [VAF]), and were not observed in the setting of a myeloid neoplasm. In contrast, *TP53* variants were detected in 4.9% of patients (10 variants in 7 of 144 evaluable patients), and these variants were seen in the setting of myeloid neoplasia in 4 of 7 patients, with 3 of those 4 developing a complex karyotype. A custom NGS panel of genes involved in DDR, senescence, and cell cycle identified additional variants, including *KAT6B*, *PALB2*, *TELO2*, and *TOP1* genes in individual patients (Supplemental Figure 1).

*U2AF1* variants involving the hotspot residue p.S34 were present in 12 (8.3%) patients with a median age of 52.5 (range 14–72). Half of these patients had a concurrent myeloid neoplasm, always cooccurring with at least 1 additional cytogenetic or sequence variant. In contrast, several patients had persistent *U2AF1* variants without progression (Supplemental Figure 2), including a young man followed from age 21 to 32 with a transient 2% VAF variant detected at ages 30 and 31, a teenage patient showing progressive expansion of *U2AF1* p.S34F from 3% to 17% from age 14 to 15.5, and a young woman followed from age 31 to 36 with a persistent *EZH2* variant of uncertain significance at 10% or less VAF, who then developed a *U2AF1* p.S34F variant alongside a transient clone with gain of 1q. All 3 of these patients had persistent cytopenias with marrow hypocellularity but without morphologic evidence of dysplasia, and the youngest ultimately underwent bone marrow transplant.

In contrast, variants in genes most common in age-related CH, *DNMT3A* and *TET2*, were relatively infrequent (3 of 144, 2.1%, and 4 of 144, 2.8%, respectively) and occurred at median age of 59 years (range 47–70), generally in line with their occurrence in the general population (27–29) (Figure 6 and Supplemental Figure 3). Of other hematologic malignancy-associated variants, *ASXL1* was mutated in 5 of 144 (3.5%) and *SF3B1* in 2 of 144 (1.4%) patients. *SF3B1* variants were MDS defining in both affected patients (30). Myeloid malignancies were diagnosed in 3 of 5 patients with *ASXL1* variants, 1 of 4 with *TET2* variants, and 0 of 3 patients with *DNMT3A* variants. Several MDS/AML-associated variants (e.g., *ETV6*, *FLT3*, and *NPM1*, identified in one patient each) were only found in patients who transformed to MDS/AML. Hematologic malignancy–associated variants were much less common in pediatric patients (5.1% versus 27.6% in adults; *P* = 0.004, Figure 6B).

### ATM-dependent DDR and senescence in TBD.

**Figure 7:**
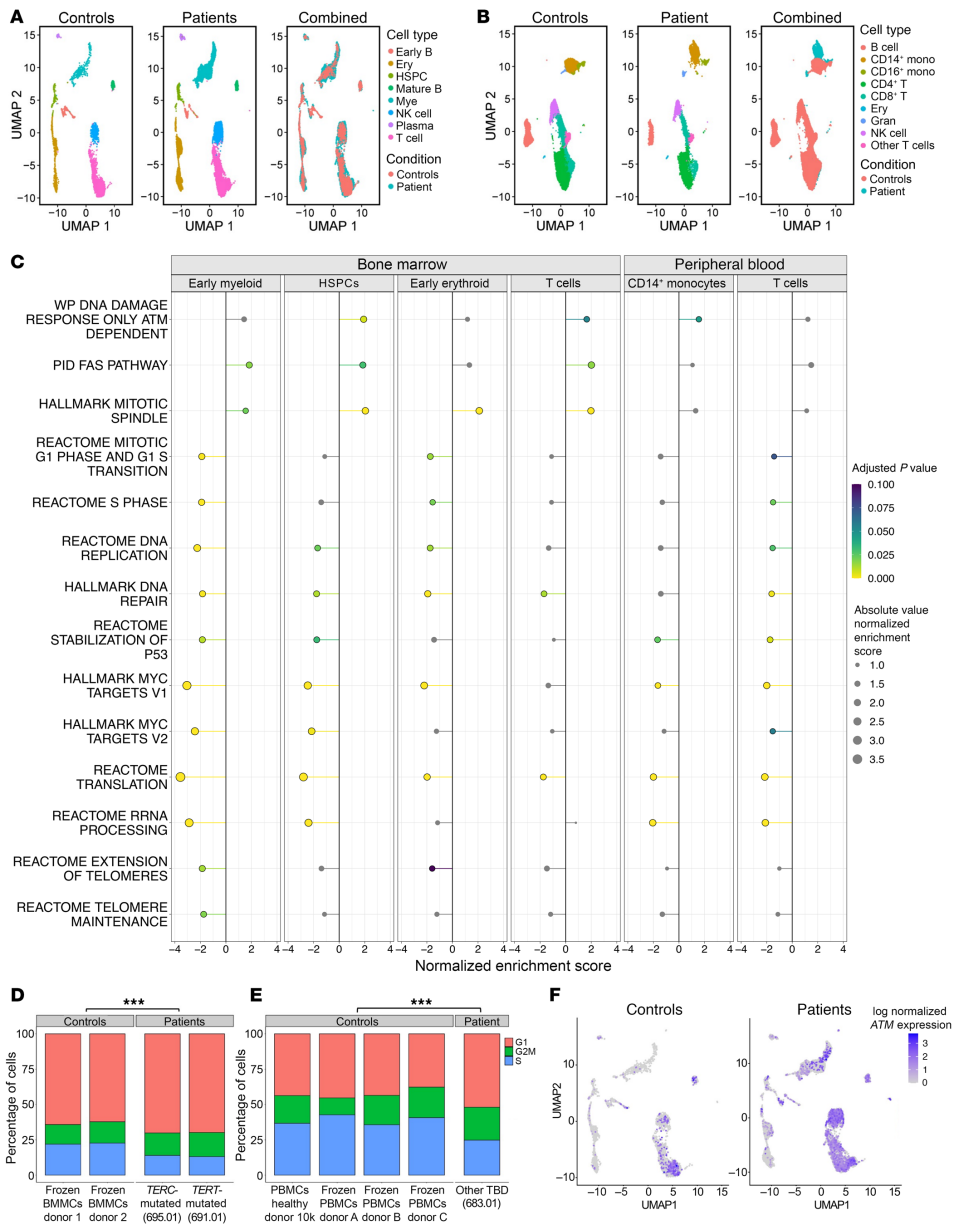
Single-cell transcriptome analysis of hematopoietic cells in TBD patients compared with healthy controls. (**A**) UMAP plot of bone marrow mononuclear cells (BMMCs) from 2 healthy controls and 2 patients with TBD. (**B**) UMAP plot of peripheral blood mononuclear cells (PBMCs) from 4 healthy controls and 1 patient with TBD. (**C**) A lollipop plot of gene set enrichment analysis (GSEA) of single-cell transcriptomes in BMMCs from 2 patients with TBD compared with 2 healthy controls (left side of the panel) and in PBMCs from 1 patient with TBD compared with 4 healthy controls (right side of panel). Shown are the results demonstrating significantly dysregulated pathways (listed on the *y* axis) across analyzed cell clusters (shown on the *x* axis in BMMCs: early myeloid, hematopoietic stem cells [HPSCs], early erythroid, T cells, and in PBMCs: Monocytes, T cells). Adjusted *P* value is indicated by a color scale, from yellow (*P* = 0.000) to teal (*P* = 0.050) to blue (*P* = 0.1), with gray representing adjusted *P* value >0.1. The absolute enrichment score is indicated by the size of the circle, and normalized enrichment score is indicated by the direction and length of the lollipop stem. ATM-dependent DDR, Fas, and mitotic spindle pathways were upregulated across subsets, with the corresponding downregulation of pathways in S phase, DNA replication, Myc targets, and translation. (**D** and **E**) Histograms showing significant differences with a reduction in S phase and increase in G2_M and G1 phases of the cell cycle in TBD patients compared with controls in BMMCs (**D**) and PBMCs (**E**). ***P < 0.001. (**F**) UMAP plot of BMMCs showing expression of *ATM* in patients versus controls.

Using single-cell transcriptomics, we explored gene-expression changes associated with CH emergence in TBD. We analyzed single-cell RNA from marrow or peripheral blood mononuclear cells from 3 patients with TBD due to different genetic causes and compared these to transcriptomes of healthy individuals (Figure 7, A–F). TBD patient cells displayed a striking downregulation of pathways associated with cell proliferation, with a reduction in transcripts associated with G1-S transition, DNA synthesis, protein translation, Myc targets, and an increased fraction of cells in G1 phase, indicating cell-cycle arrest (Figure 7, C–E). Several senescence-associated genes were significantly upregulated in TBD patients compared with controls (Supplemental Figure 4). The ATM-dependent DDR pathway was significantly upregulated in hematopoietic stem and progenitor cells (Figure 7, C and F) with the same trend observed across all analyzed cell subsets (Figure 7C).

**Figure 8:**
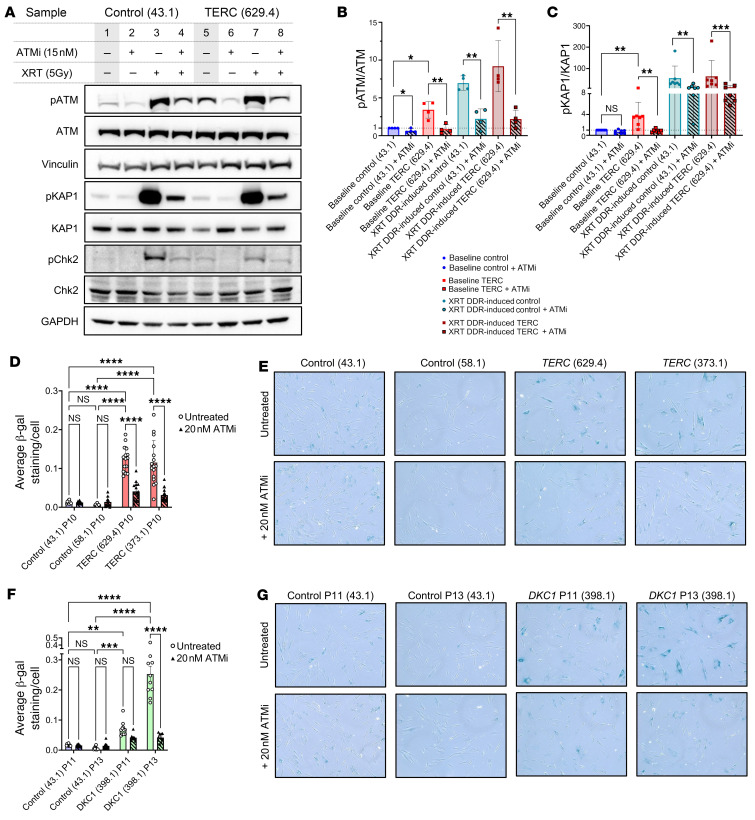
Increased ATM target phosphorylation in *TERC*-mutated compared with control fibroblasts. (**A**) A representative Western blot image showing phosphorylation of ATM target proteins in *TERC*-mutated compared with control fibroblasts. Experimental condition (+/- ATMi) and experimental induction of DDR using x-ray radiation (XRT) are shown at the top of each lane. Vinculin was used as a loading control for ATM, and GAPDH was used as a loading control for KAP1 and Chk2. (**B** and **C**) Image densitometry analysis of replicate Western blot experiments was performed in ImageJ and summarized in **B** for pATM/ATM, *n* = 4 and (**C**) for pKAP1/KAP1, *n* = 7. Quantified densitometry values for each protein were normalized to their respective loading control. The ratio of phosphorylated compared with total protein levels in each experiment, along with the summary statistics (mean and SD) are shown in bar plots. Statistical analysis was performed using 2-tailed paired *t* tests. (**D**) Quantitative analysis of average senescence-associated β-galactosidase expression, normalized per cell number in passage 10 primary skin fibroblasts from 2 non-TBD controls and 2 TBD patients bearing different *TERC* variants grown with or without 20 nM ATMi; (**E**) Shown are representative β-galactosidase staining images. (**F**) Quantitative analysis of average senescence-associated β-galactosidase expression, normalized per cell number, in primary skin fibroblasts from non-TBD control and a TBD patient with DKC1 mutation, alongside representative images (**G**), demonstrating the progressive increase in senescence-associated β-galactosidase expression with successive passages in TBD patients’ cells that is significantly alleviated by 20 nM ATMi. In **D**–**G**, *n* = 10–16 wide-field images at 10× magnification per each condition, quantified in Fiji. Statistical analysis was performed in GraphPad Prism using 2-way ANOVA. *P < 0.05; **P < 0.01; ***P < 0.001; ****P < 0.0001.

**Figure 9:**
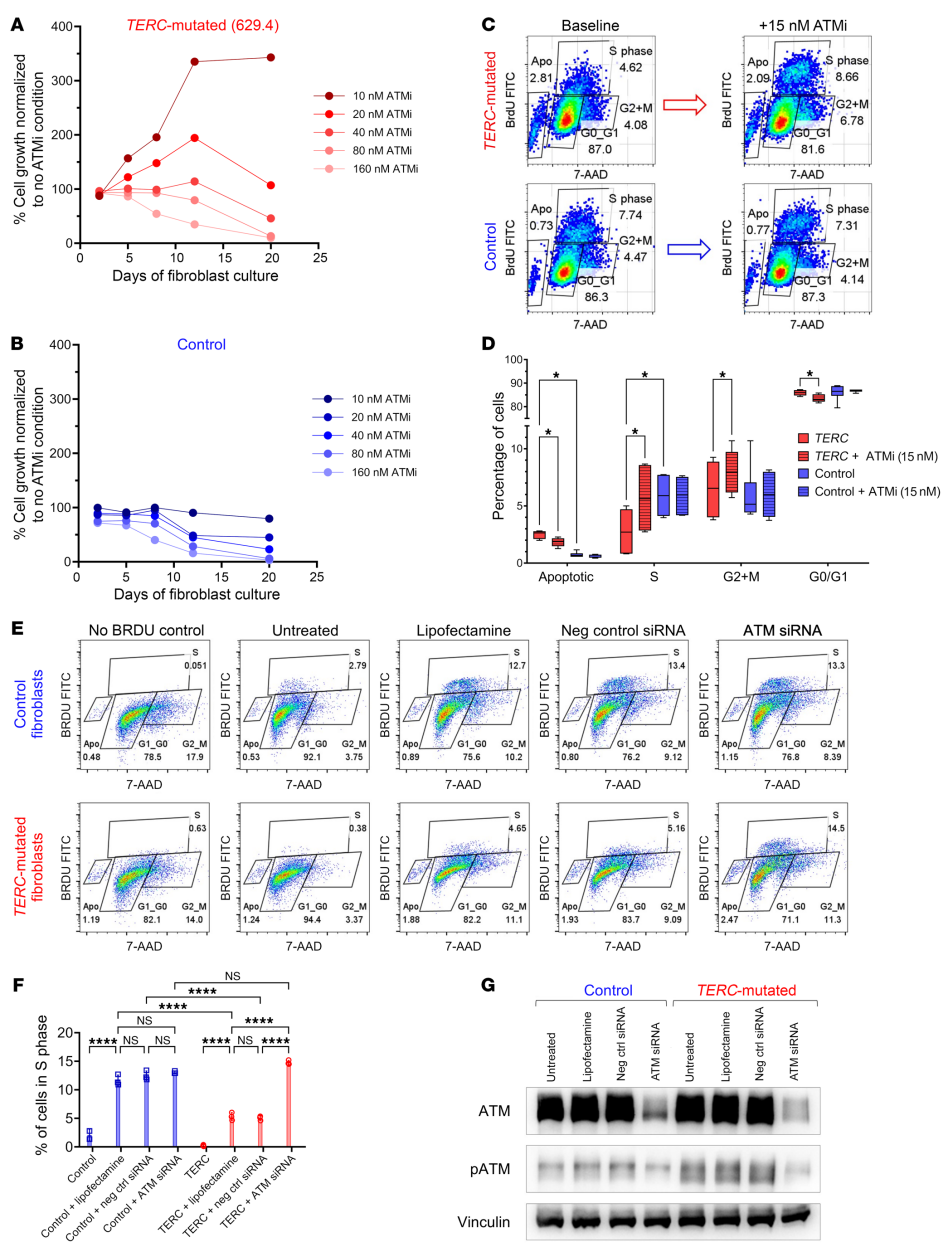
Low-dose ATM inhibition and siRNA knockdown selectively improve growth of TBD fibroblasts. (**A** and **B**) Results of a representative growth assay, in which low-passage primary skin fibroblasts from a *TERC*-mutated patient (**A**) or a control (**B**) were grown in log phase with or without ATM inhibitor (ATMi) at a range of doses from 10 to 160 nM. ATMi at low doses of 10–20 nM significantly increased growth of *TERC*-mutant (**A**) but not control fibroblasts (**B**). At higher doses, ATMi demonstrated dose-dependent toxicity in long-term growth assays (*n* = 2 independent dose-titration replicate experiments performed, each with 5 ATMi doses). (**C**) Representative flow cytometry results of a BrdU incorporation cycle analysis in *TERC*-mutated and control fibroblasts growth without ATMi (“baseline”) and with 15 nM ATMi. (**D**) Summary statistics of BrdU incorporation cell-cycle experiments demonstrating a significant increase in *TERC*-mutated fibroblasts entering S- and G2+M cell cycle phases with concomitant reductions in G1 and apoptotic (Apo) cells with ATMi (*n* = 6). Statistical analysis was performed using 2-tailed paired *t* tests, with multiple comparisons adjustment performed using the FDR, calculated using the 2-stage step-up (Benjamini, Krieger, and Yekutieli) method. *q < 0.05. (**E**) Representative flow cytometry results of a BrdU incorporation cycle analysis in *TERC*-mutated and control fibroblasts treated with siRNAs targeting ATM, nontargeting control siRNA, vehicle (lipofectamine) alone, and untreated cells. (**F**) Summary statistics for cell cycle analysis with ATM knockdown demonstrating a significant increase in cells entering S-phase of the cell cycle in *TERC*-mutated fibroblasts but not control fibroblasts treated with siRNA-targeting ATM (*n* = 3). Analysis done with 1-way ANOVA with the Šidák’s multiple-comparisons test. ****adjusted *P* < 0.0001. (**G**) Western blot showing successful partial knockdown of ATM with ATM-targeting siRNA but preserved levels of ATM in cells treated with nontargeting control siRNA or lipofectamine alone.

We tested the phosphorylation of ATM targets in TBD patient fibroblasts to confirm our findings of ATM-dependent DDR activation. Consistent with RNA-Seq data, *TERC*-mutated fibroblasts showed significantly higher ATM activity, with higher autophosphorylated ATM and phospho-KAP1 levels compared with control fibroblasts (Figure 8, A–C). Compared with control fibroblasts, TBD fibroblasts grew more slowly approaching senescence (Supplemental Figures 5–7), had higher expression of senescence-associated β-galactosidase (Figure 8, D–G), and had more apoptotic cells and fewer S-phase cells, consistent with the induction of ATM-dependent DDR and cell-senescence pathways (Figure 9).

### Loss-of-function somatic ATM variants in TBD.

**Figure 10:**
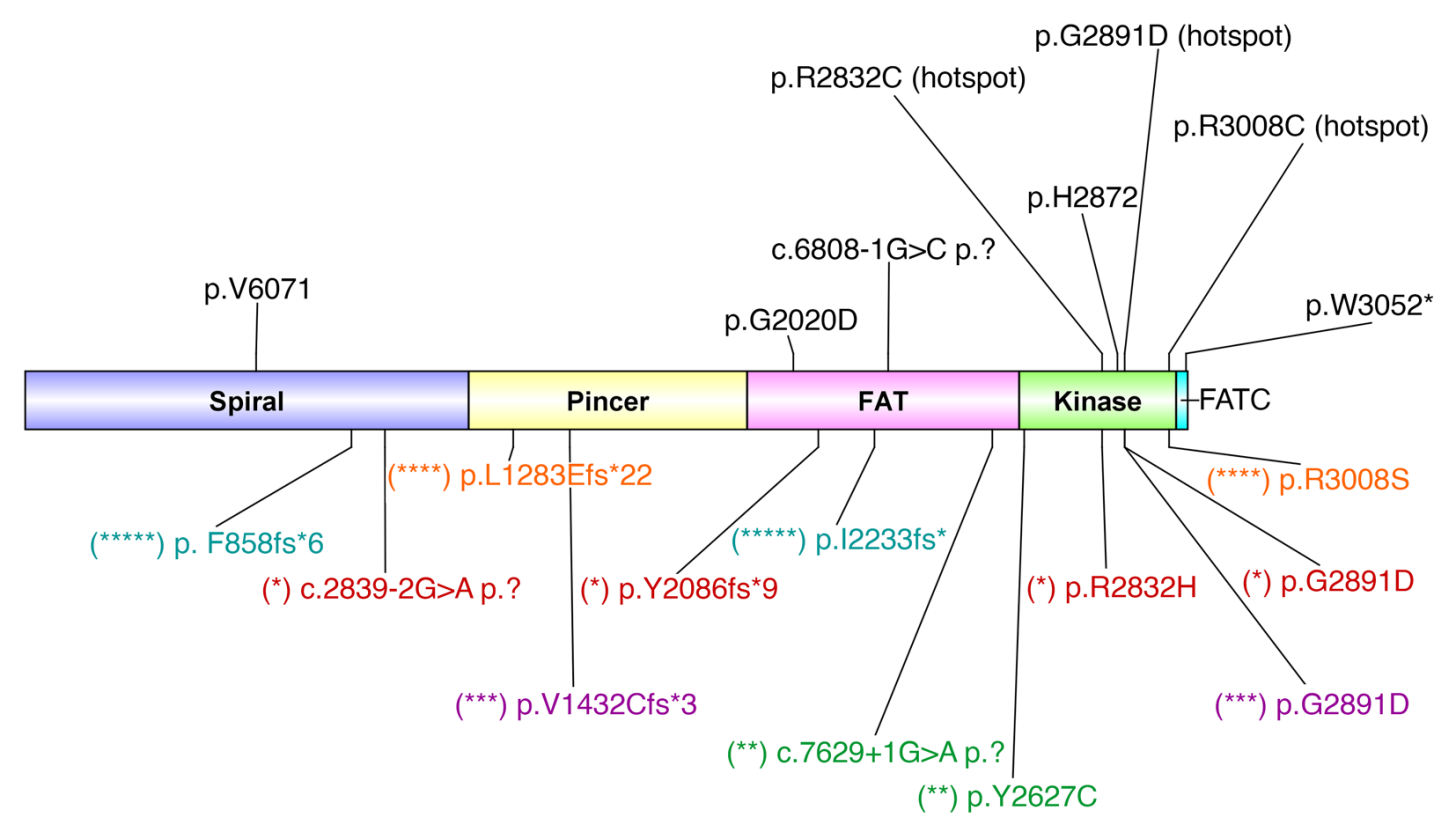
ATM protein structure with 20 somatic variants identified in TBD patients. A diagram of ATM protein structure, showing the defined ATM domains and the 20 identified somatic variants. Variants that were identified in the same patient are shown underneath the protein structure in different colors and designated by different numbers of asterisks (*, **, ***, ****, *****), corresponding to different patients. ATM residues disrupted by more than one variant are labeled with a “hotspot” designation. The diagram was created with DOG version 2.0.1 software (79).

We hypothesized that somatic *ATM* variants allow TBD mutant cells to overcome hyperactive ATM-dependent DDR by reducing ATM function. As predicted, 9 of the *ATM* variants were presumed loss of function due to nonsense, frameshift, or splice site variants, while 11 were amino acid substitutions, 9 of which were in the kinase domain (Figure 10). Of the missense variants, all were previously reported as either likely pathogenic (*n* = 3) or of uncertain significance (*n* = 4) (Supplemental Table 4). Seven variants involved 1 of 3 hotspot kinase domain residues (p.R2832 [*n* = 2], p.G2891 [*n* = 3], and p.R3008 [*n* = 2]). The 20 somatic *ATM* variants were seen in 12 patients, with a median of 1.5 variants per patient (range 1–4). Three patients had exposure to immunosuppressants for lung disease prior to the identification of their *ATM* variants, while the remaining 9 had no prior immunosuppression (Supplemental Table 5).

Patients with somatic *ATM* variants had a high rate of systemic TBD manifestations. Seven of 12 *ATM*-mutant patients (58%) had ILD, 3 of 12 (25%) had cirrhosis, and 4 (33.3%) died of complications of TBD. While the rates of these complications trended higher in patients with *ATM* variants compared with those without (Figure 5), patients with *ATM* variants were older (56 years [16–78 years] versus 43 years [3–77 years]) and our study was underpowered, precluding multivariate analysis stratifying by age and TBD genetic subtype. Germline *TERC* variants were found in 7 of 12 (58.3%), 5 of whom developed multiple somatic variants in *ATM* (4 patients had 2 variants and 1 patient had 4 variants). One patient with a germline *TERC* variant had a second germline variant in *ZCCHC8*, and in the other 5 patients, germline variants were in *ZCCHC8* (in 1 patient), *TERT* (in 1), and *RTEL1* (in 1), and 2 patients had no established genetic diagnosis.

**Figure 11:**
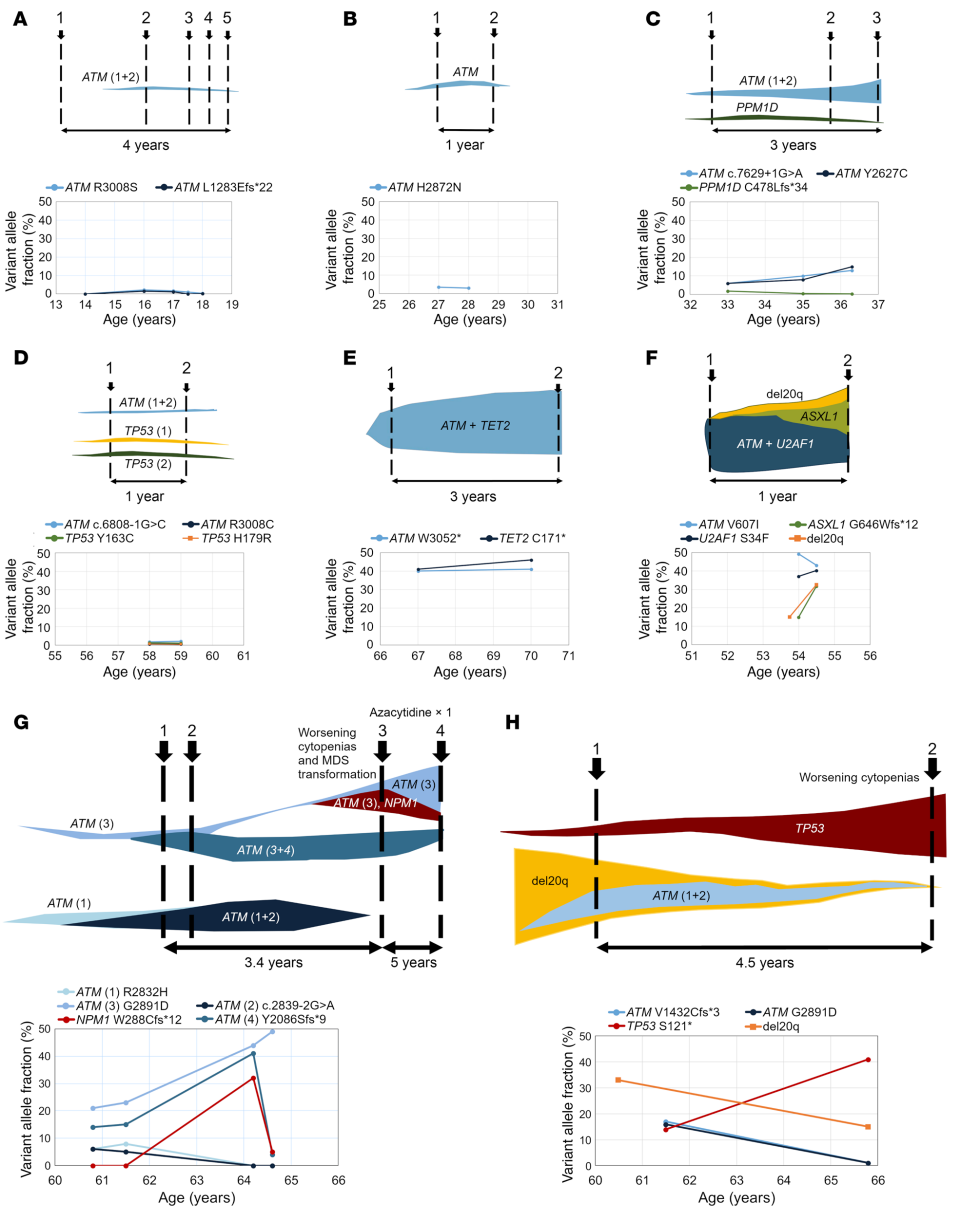
Longitudinal follow-up and clonal evolution in 8 patients with somatic *ATM* variants. (**A**–**H**) VAF graphs showing the VAF of the identified variants at each analysis time point, indicated by patient age. For patients with chromosomal abnormalities, the VAF was estimated as 0.5 times the number of cells carrying a cytogenetic abnormality. Above each of the VAF plots shown are the corresponding stylized drawings depicting the inferred or experimentally confirmed clonal structure. (**A**–**E**) Shown are the longitudinal follow-up analyses for 5 patients with stable hematologic parameters and no malignant transformation. Of these, clonal structures for the patients in **C** and **G** were experimentally confirmed, and for others, the clonal structure was inferred based on variant VAFs and VAF dynamics over time. Results of single-cell DNA and protein sequencing for the patient in **C** are shown in Figure 9. (**F**–**H**) Shown are longitudinal clonal dynamics for 3 patients with somatic *ATM* variants who progressed to MDS. (**F**) A 54-year-old with cytopenias and MDS-MLD. (**G**) A 60-year-old who, following a 3.4-year period of stable blood counts, developed worsening cytopenias and transformation to MDS. Clonal architecture analysis after MDS progression revealed a new subclonal acquisition of an *NPM1* variant in cells with *ATM* p.G2891D. (**H**) A 60-year-old with stable blood counts for 4.5 years, after which developed worsening cytopenias and MDS progression.

**Figure 12:**
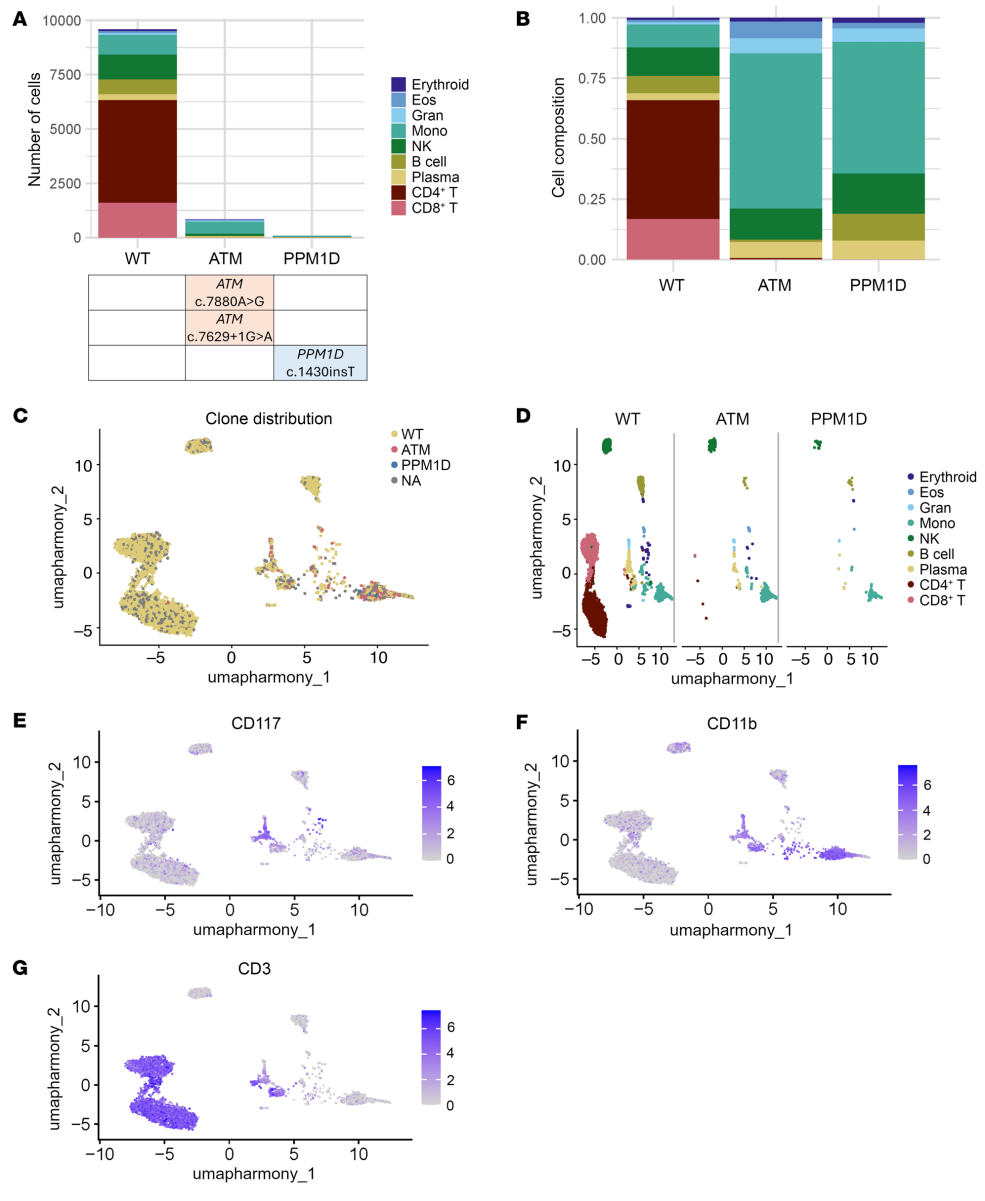
Clonal architecture and hematopoietic contributions of cells with and without somatic *ATM* variants in peripheral blood of a *TERC*-mutated TBD patient analyzed using single-cell DNA and protein sequencing on the Tapestri platform. (**A**) Bar graph showing the numbers of cells with the genotypes indicated on the *x* axis, and the cell types identified by surface protein staining on the *y* axis. Both of the somatic *ATM* variants were identified within the same clone. (**B**) Shown are the relative hematopoietic cell distributions of WT, *ATM*-mutant, and *PPM1D*-mutant clones, showing that *ATM*-mutant and *PPM1D*-mutant clones were highly skewed to myeloid cells, as compared with cells without either *ATM* or *PPM1D* variants. (**C**–**G**) UMAP plots depicting the clone distribution of the WT, *ATM*-, and *PPM1D*-mutant cells, overlaid in **C** and shown separately with color coding indicating the corresponding hematopoietic cell subsets. (**E**–**G**) Distribution of CD117 (immature myeloid and mast cell marker c-kit), myeloid cell marker CD11b, and T-lymphocyte cell marker CD3 expression, respectively.

Two of the *TERC*-mutant patients with somatic *ATM* variants were a father and his daughter, and both developed somatic *ATM* variants (4 different somatic variants in the parent and 2 distinct variants in the daughter). Clonal architecture analysis of the parent demonstrated that his 4 somatic variants were grouped in 2 clones with heterozygous ATM inactivation, each with a subclone with 2 *ATM* variants (presumed in *trans*) (Figure 11 and Supplemental Figures 8 and 9). Clonal architecture analysis of the child, performed using single-cell DNA and protein sequencing, revealed that both of her *ATM* variants were within the same clone, which exhibited a highly skewed hematopoietic differentiation toward myelopoiesis compared with unmutated cells (Figure 12).

### ATM variants alone are not sufficient for malignant transformation.

**Figure 13:**
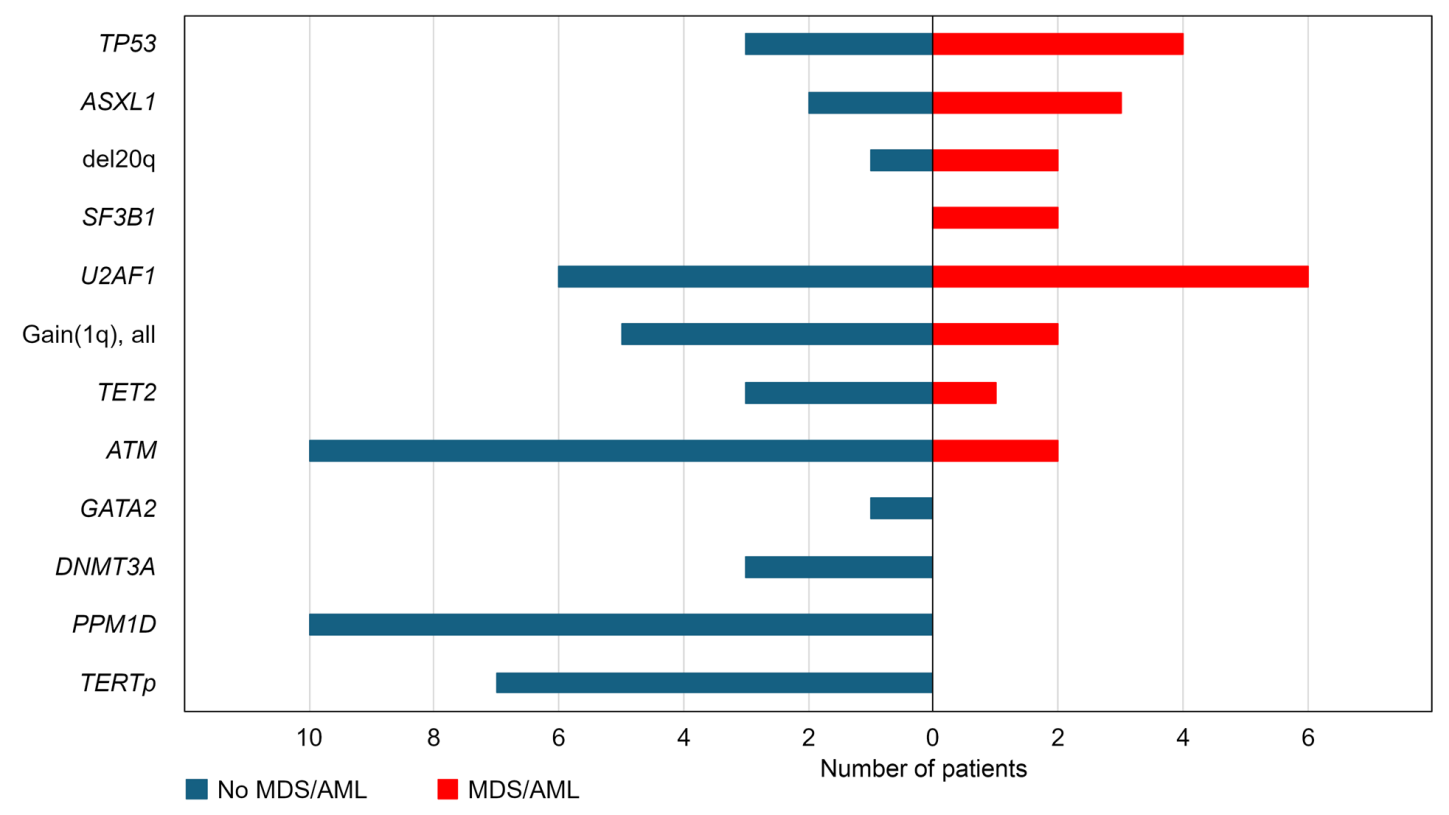
Overall statistics for patients with and without MDS progression. A bar plot illustrating the number of patients carrying somatic alterations shown on the *y* axis in TBD patients without hematologic malignancies (left, in blue) and corresponding number of patients where specific alterations were identified at the time of MDS/AML (right, in red). For the patient in Figure 11H, where the somatic *ATM* variant was not within the MDS-initiating clone, *ATM* was counted as non-MDS/AML for this bar plot.

Of the 12 patients with *ATM* variants, 8 were followed longitudinally. Of them, 5 had stable blood counts without malignant progression (Figure 11, A–E). However, 3 patients with *ATM*-mutant clones developed MDS (Figure 13). One presented with MDS and had multiple MDS-associated alterations: del(20)q, *U2AF1* p.S34F, and *ASXL1* variants, along with an *ATM* variant at high VAF (confirmed to be somatic by testing of constitutional tissue) (Figure 11F). The second patient had 4 somatic *ATM* variants as described above; following 3.4 years of stable blood counts, he developed worsening cytopenias with bone marrow examination confirming MDS transformation. Clonal architecture analysis revealed acquisition of an *NPM1* variant in cells with the hotspot *ATM* p.G2891D variant (Figure 11G and Supplemental Figures 8 and 9). In the third patient with MDS, the malignant clone did not arise from *ATM*-mutant cells; their progression to MDS was accompanied by the expansion of a *TP53*-mutated clone, while the VAFs of both somatic *ATM* variants diminished (Figure 11H). In the latter 2 patients who were followed prospectively and developed MDS during the study, somatic *ATM* variants were identified incidentally after lung transplant in their 60s on their routine screening bone marrow biopsies. Both patients later died of complications of pulmonary disease and MDS.

Five of the 12 patients with somatic *ATM* variants were identified as adolescents/young adults (ages 15–37). Two of these patients had end-stage lung disease: both were incidentally found to have somatic *ATM* variants in the process of lung transplant evaluation, and 1 died from pulmonary complications before receiving a lung transplant. The three other young patients are well with stable cytopenias without further clonal evolution with more than 2 years of follow-up.

### ATM inhibition overcomes telomere dysfunction-induced DDR and replicative senescence.

To test the hypothesis that ATM inhibition can allow TBD cells to overcome DDR-mediated senescence, we evaluated the effects of ATM inhibitor AZD0156 (ATMi) on fibroblasts from TBD patients. Without ATMi, TBD patient fibroblasts exhibited increased phosphorylation of ATM targets (Figure 8). ATMi at low doses of 10–20 nM inhibited ATM target phosphorylation (at baseline and in a positive control condition when ATM-dependent DDR was experimentally induced by radiation) and dramatically improved the growth of *TERC*- and *DKC1*-mutant fibroblasts without affecting control fibroblasts (Figure 8, Figure 9, A and B, and Supplemental Figures 5–7). Growth assays with higher ATMi doses (40–160 nM) demonstrated dose-dependent toxicity in control and TBD cells. Low-dose ATMi increased the number of TBD fibroblasts entering S and G2/M cell cycle phases with concomitant reductions in G1 and apoptotic cells (Figure 9, C and D) with reduced β-galactosidase (Figure 8, D–G), indicating that ATM inhibition allows TBD cells to partially overcome telomere dysfunction–associated replicative senescence. Partial siRNA knockdown of ATM in TBD fibroblasts replicated the results obtained with ATMi, showing significantly increased S phase entry in TBD but not in control fibroblasts (Figure 9, E–G). Cytogenomic arrays showed no chromosomal instability after approximately 21 days of ATMi treatment in culture (median 7.7 population doublings for *TERC*-mutated and 3.3 for *DKC1*-mutated lines), with no acquired copy number changes or CN-LOH to account for improved growth.

**Figure 14:**
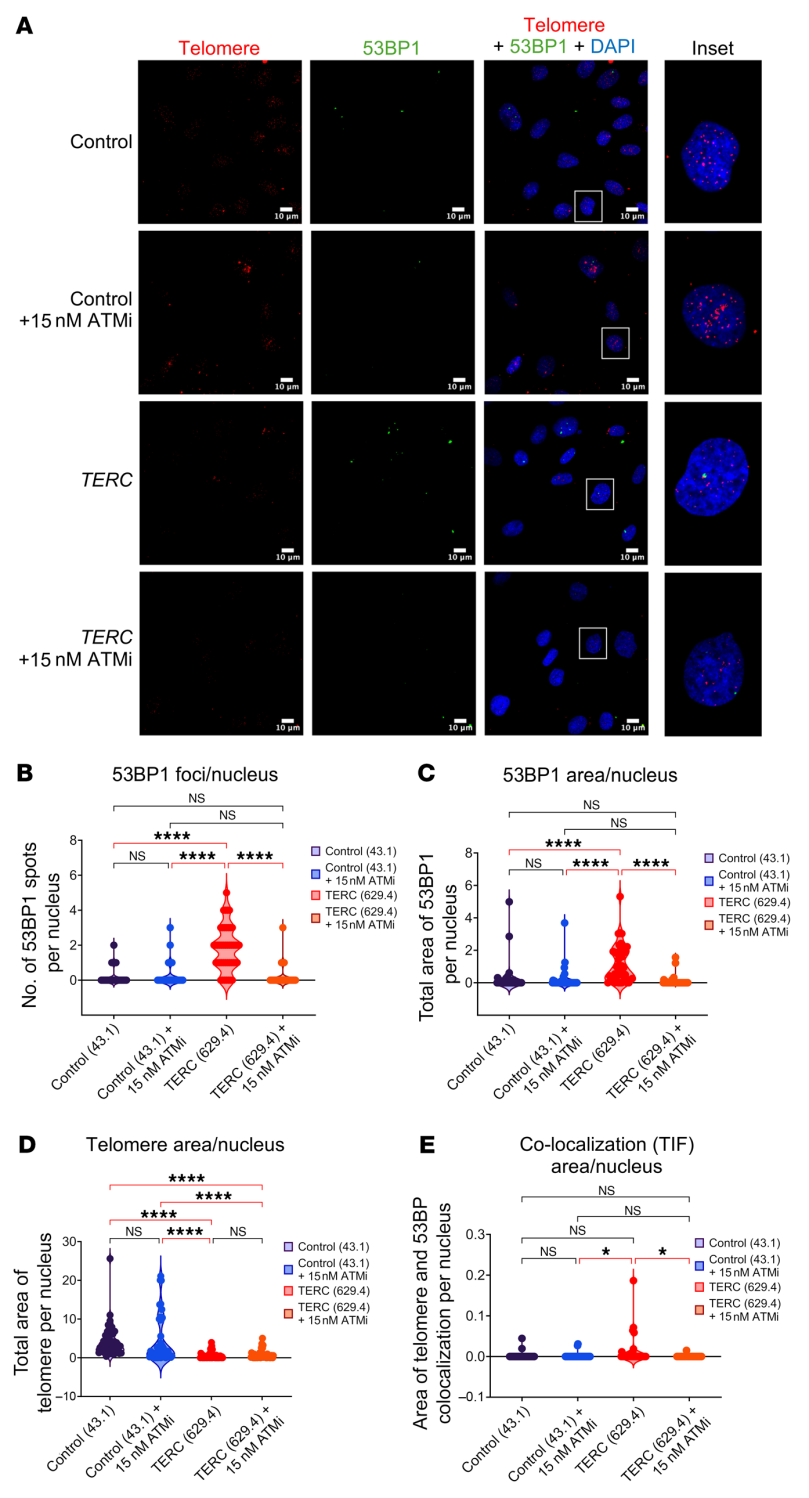
Induction of DDR response in response to dysfunctional telomeres in TBD. (**A**) Immunofluorescence staining of low-passage control versus *TERC*-mutated fibroblasts grown in log phase with and without ATMi. Cells were stained with telomere peptide nucleic acid probe (red), anti-53BP1 antibody (green), or DAPI (blue). Quantitative analysis was performed in Fiji, for 45 nuclei for each condition, with summary statistics of nuclear 53BP1 foci (**B** and **C**), telomere (**D**), and colocalized 53BP1 and telomere foci (**E**). Statistical analysis was performed using ANOVA. *P < 0.05; ****P < 0.0001.

Dysfunctional telomeres can be recognized as DNA breaks by DDR factors, such as 53BP1. Thus, we examined 53BP1 deposition in *TERC*-mutated and control patient fibroblasts with and without ATMi (Figure 14). As expected, *TERC*-mutated fibroblasts had reduced telomere fluorescent in situ hybridization staining; furthermore, their 53BP1 staining was significantly increased compared with control fibroblasts (Figure 14, A–D). Both the 53BP1 foci and the areas of 53BP1 colocalization with telomeres (telomere dysfunction-induced foci, aka TIFs) were significantly reduced by ATMi (Figure 14, B, C, and E).

Our results suggest that telomere dysfunction-induced ATM-dependent DDR is one of the major causes of impaired cell fitness in TBD and can be alleviated by low-dose inhibition of ATM.

## Discussion

Through our analysis of CH in samples from 166 pediatric and adult patients with TBD, we identified downregulation of the ATM-dependent DDR as a mechanism of somatic genetic rescue of accelerated cell senescence in patients with telomere dysfunction. Loss-of-function variants in *ATM* and other DDR genes were identified in 21.5% of patients, accounting for 39.2% of our CH findings. Partial pharmacologic ATM inhibition and ATM knockdown, modeling the effects of somatic *ATM* variants in TBD patients, improved the fitness of TBD patient cells by allowing them to bypass DDR-mediated replicative senescence. Together with single-cell transcriptomics in patient hematopoietic cells and assays of ATM activation, TIFs, cell growth, and senescence, our results highlight the central role of ATM-dependent DDR in TBD cell biology and suggest dampening hyperactive ATM-dependent DDR as a potential therapeutic intervention in TBD.

Our study contributes to the growing body of knowledge of CH in TBD. Previously, 3 classes of recurrent somatic alterations in TBD were defined, and these were similarly identified in our cohort: (a) variants in telomere maintenance genes present in 7.6% of evaluable patients; (b) spliceosome variants in 10.4% of patients, mainly *U2AF1* p.S34; and (c) chromosomal abnormalities in 20.2% of patients, interestingly, with 5.9% of patients having gain of chromosome arm 1q (reminiscent of 1q duplication involving *MDM4* in Fanconi anemia; ref. 31) observed both as a solitary alteration without malignant progression and within complex karyotype after malignant transformation. We now report variants in the DDR pathway, and particularly ATM inactivation, as a fourth major class. Interestingly, we identified a somatic mutation in *TELO2*, which may similarly allow TBD cells to bypass senescence by partial loss of ATM, as *TELO2*-encoded protein TEL2 was previously shown to be required for ATM stability in mammalian cells (32). Patients with somatic DDR pathway mutations were severely affected by TBD, with most adults with somatic *ATM* variants carrying a diagnosis of ILD and/or BMF. Thus, somatic *ATM* and other somatic DDR gene variants may represent de facto biomarkers of DDR activation in TBD patients and a biological readout of the severity of telomere dysfunction. These data further suggest that DDR likely contributes not only to BMF but also to ILD and organ dysfunction in TBD, a concept that should be explored in future studies. Notably, while our manuscript was under review, a preprint by Stuart and de Lange reported that healthy human primary lung fibroblasts grown to the Hayflick limit and having reached their replicative senescence can also be induced to undergo apparently normal cell divisions by inhibiting ATM (33).

While individual TBD patients with somatic *ATM* variants have been noted in previous studies (5), recurrent *ATM* variants in TBD have not been reported. This may be explained by the exclusion of *ATM* and most non-*TP53* DDR genes from hematologic malignancy panels traditionally used to evaluate CH in the clinic, genetic heterogeneity of TBD patients included across different studies, and patient age differences across studies. In our cohort, *ATM* variants were most common in adults and in *TERC*-mutated TBD. Interestingly, CH involving DDR genes was previously reported in a study of lung transplant recipients, a subset of which had genetically confirmed TBD (34). However, telomere length testing was not performed for those patients, and we speculate that a subset, particularly those with ILD, may have had an underlying TBD that could not be confirmed genetically. The different results across published CH studies in TBD underscore the challenges of studying a disease that spans the full age spectrum and is clinically and genetically heterogeneous. Our findings highlight the importance of comprehensive evaluation and monitoring of germline and somatic genetics, the use of multimodal approaches, and the inclusion of TBD- and DDR-associated genes in clinical somatic NGS panels.

The effects of ATM loss in the context of critically short telomeres are likely multifold. Our findings, which show a competitive advantage for *ATM*-mutant cells in TBD, may seem unexpected given the earlier report by Lee and colleagues that ATM is necessary for de novo telomere elongation in telomerase-expressing mouse fibroblasts and human cell lines in vitro (35). However, several factors may help reconcile our results with these previous in vitro studies. While Lee et al. demonstrated that ATM is required for telomere elongation in mouse telomerase-expressing fibroblasts (35), their earlier work also suggested that ATM is not essential for rescuing the shortest telomeres (36). This could be due to compensatory roles played by overactive ATR or other mechanisms. ATM-independent telomere maintenance has also been observed in immortalized cell lines from ataxia-telangiectasia patients, which lack ATM protein but maintain telomere homeostasis through alternative pathways (37). Furthermore, studies of individuals with heterozygous constitutional *ATM* variants revealed that, although lymphocytes from ataxia-telangiectasia patients exhibit accelerated telomere attrition with age, obligate carriers of pathogenic *ATM* variants showed no significant differences in telomere length compared with healthy controls (38). Similar findings were confirmed in a recent study, which found no evidence of increased telomere attrition in *ATM* haploinsufficient individuals (39). Collectively, these data suggest that partial loss of ATM is unlikely to have a detrimental effect on telomere length in TBD independent of other factors (for example, its effect on increased proliferative capacity).

Currently, TBD management focuses on treatment of organ dysfunction, cancer screening, and supportive care, considering organ transplantation if end-stage complications develop (40, 41). However, even after solid organ or bone marrow transplant, patients may continue to suffer from dysfunction in other organs, and some patients cannot receive a transplant due to malignancy or multiorgan dysfunction (42–49). Systemic therapeutics that modify the clinical course in TBD (arguably, the greatest need for this patient population) are lacking. While androgens are frequently used to treat cytopenias, whether they have a disease-modifying effect on telomere length remains unclear (40, 50–55). Recent preclinical studies identified several new and promising pharmacologic strategies aimed at telomere elongation by inhibiting poly(A) polymerase PAPD5 (56) or modulating cellular thymidine levels (57). However, the success of these approaches requires *TERT* expression and active telomerase activity. This would require sufficient telomere elongation within *TERT*-expressing stem cells to overcome DDR and for the effects to be sustained in the mature cell progeny long enough to improve organ function. Our results suggest a complementary strategy to improve cell fitness in TBD in a manner that does not require telomerase expression and uses partial ATM inhibition to bypass ATM-dependent DDR and senescence pathways. A similar approach targeting DDR via ATM inhibitors or telomeric noncoding RNAs has shown promise in other syndromes of premature aging, such as Hutchinson-Gilford progeria and Werner syndromes (58–60).

Would ATM inhibition promote carcinogenesis? Ataxia-telangiectasia patients and Atm-deficient (*Atm*^–/–^) mice are predisposed to cancer and are infertile due to distorted meiotic telomere clustering leading to spermatogenesis arrest (61, 62). Interestingly, while late-generation mice doubly null for *Atm* and *Terc* (*Atm*^–/–^ *Terc*^–/–^) have chromosomal instability, cachexia, and reduced survival, they are also tumor resistant with suppression of thymic lymphomas compared with early-generation (G0) double-mutant *Atm*^–/–^ *Terc*^–/–^ or single-mutant *Atm*^–/–^ mice (63–65). The tumor-resistant phenotype of *Atm*^–/–^ *Terc*^–/–^ mice contrasts with the increased rate of epithelial tumors in *tp53*-deficient *Terc*^–/–^ mice compared with *Terc*^–/–^ mice (66, 67). The differing effects of Atm versus tp53 deficiency in *Terc*-null mice have been attributed to the retention of an Atm-independent p53 checkpoint response in mice deficient for *Atm* (68). Importantly, unlike *Atm*-null mice, TBD patients with somatic *ATM* variants have only partial ATM loss, and these variants were not associated with detectable chromosomal instability and did not cause malignancy in isolation. Somatic *ATM* variants in our cohort usually occurred as single *ATM* variants, and, even in patients with multiple somatic *ATM* variants, these variants were predicted to preserve partial ATM function. It is thus possible that the benefits of partial pharmacologic ATM inhibition would outweigh any increased risk for cancer. Given that *ATM* variants are relatively common, future studies of genetic interactions and possible disease-modifying effects of germline *ATM* variants in TBD could be informative.

Our study has limitations. Although we have accrued a large and genetically diverse, multi-institutional cohort of TBD patients, each genetic TBD subtype consisted of a limited number of patients. Our genetic testing used a combination of NGS techniques, some of which had limited analytical sensitivity, only detecting clones over 4% VAF. Our analytical sensitivity is in line with most clinically relevant methods, but we acknowledge that our study may underestimate the prevalence of smaller clones. Furthermore, not all patients were evaluated with all testing modalities due to the evolution of sequencing panels and methods combined with limited sample availability. Limited longitudinal follow-up and the largely cross-sectional nature of our cohort mean that the percentages of clinical features and clonal findings should be considered conservative estimates. Similar to other retrospective cross-sectional TBD cohorts, our analysis of the risk of MDS transformation is confounded by the potential ascertainment and referral biases, whereby TBD patients with MDS progression are more likely to come to medical attention. These limitations are inherent to TBD itself, particularly as more patients are recognized late in life in the setting of end-stage ILD, while others may have been treated with bone marrow transplant earlier in life, leading to gaps in sample collection along the age spectrum. Future studies must address these gaps through prospective, longitudinal, comprehensive genetic monitoring and correlation with clinical disease manifestations through collaborative studies.

In conclusion, our study revealed several canonical pathways of premalignant clonal evolution in TBD. We identified recurrent somatic *ATM* variants as a mechanism of somatic genetic rescue in TBD patients, allowing the cells to overcome telomere dysfunction–induced ATM-dependent DDR activation. Our results support future studies of pharmacologic downmodulation of ATM-dependent DDR as a new therapeutic strategy in TBD.

## Methods

### Sex as a biological variable.

Our study examined both male and female TBD patients, with the exception of X-linked recessive TBD caused by *DKC1* variants, for which only male patients were enrolled.

### Cohort recruitment.

TBD was diagnosed according to standard guidelines, including documentation of low median lymphocyte telomere lengths for age by flow-FISH in conjunction with characteristic clinical findings (e.g., BMF, ILD) (Figure 1, Table 1, and Supplemental Tables 1 and 2) (1, 2).

### NGS.

As available, peripheral blood or bone marrow DNA was tested for somatic variants using panel-based NGS or WES (Supplemental Tables 6 and 7). Somatic variants identified via 305-gene research panel were confirmed by Sanger sequencing of paired hematopoietic and constitutional DNA (Supplemental Figure 1).

### Single-cell RNA-Seq.

Peripheral blood or bone marrow mononuclear cells from 3 patients with genetically different TBD (autosomal-dominant *TERC*, *TERT*, and unknown genetic defect) were tested with single-cell RNA-Seq (scRNA-Seq) using the 10X Genomics platform. Patient data were harmonized with unaffected control datasets (69) (Supplemental Table 8) using Harmony (70), clustered, and annotated with cell type. Differential expression and enrichment analyses were performed using Seurat V4 (71) (https://www.github.com/satijalab/seurat), DESeq2 (72), and fGSEA (73) workflows.

### Single-cell DNA and protein sequencing.

Flow cytometrically sorted CD45^+^ peripheral blood mononuclear cells from a *TERC*-mutant patient with 2 *ATM* and 1 *PPM1D* variants underwent single-cell DNA and protein sequencing on the Tapestri platform (Mission Bio). Patient data were analyzed using the Tapestri (https://portal.missionbio.com/), version 3.4, DNA + Protein pipeline, scDNA (https://github.com/bowmanr/scDNA) and Harmony R packages (https://portals.broadinstitute.org/harmony/), and Seurat.

### Cell culture, ATM inhibition, and ATM knockdown.

Primary skin fibroblasts from 6 patients with TBD (3 with *TERC* and 3 with *DKC1* variants) and 4 non-TBD controls were grown under standard conditions in the presence or absence of ATM inhibitor ATMi (Selleck Chemicals) at 10–160 nM concentrations. ATM pathway activation and the effect of ATMi were measured by Western blot of phosphorylated targets of ATM. Irradiated cells served as a positive control for ATM induction. The effects of ATMi on cell growth were analyzed using growth curves in log phase. At the end of culture, cells were analyzed for genomic instability: conventional karyotype analysis failed due to a lack of cell proliferation, and chromosome analysis was done using cytogenomic arrays. Cell cycle was analyzed with BrdU incorporation assay (Phase-Flow FITC BrdU Kit, catalog 370704; BioLegend). ATM knockdown was performed using 10 nM ON-TARGETplus-SMARTpool siRNAs to ATM or the Non-Targeting Control-Pool (Dharmacon) to transfect TBD patient and control skin fibroblasts, with cells collected 72 hours after transfection for Western blotting and cell-cycle analysis.

### Senescence β-galactosidase cell staining.

Primary skin fibroblasts from TBD and non-TBD control patients were grown in 35 mm cell culture dishes to 60%–75% confluence under standard conditions, with and without 20 nM ATMi, followed by DAPI and β-galactosidase staining using the Senescence β-Galactosidase Cell Staining Kit (Cell Signaling Technology, catalog 9860), according to the manufacturer’s instructions. Imaging was performed on a Zeiss Widefield Microscope at ×10 magnification. Quantitative analysis of β-galactosidase staining was carried out in Fiji/ImageJ, version 1.54 (NIH), by automated quantification of β-galactosidase staining signa, normalized by the number of cells quantified by DAPI staining, similarly to what was previously described (74) using the custom Fiji macros (see Supplemental Methods).

### Telomere dysfunction-induced foci analysis.

Early passage patient and control fibroblasts grown in log phase with or without ATMi were stained with peptide nucleic acid (PNA) probe to the telomere repeat TTAGGG (Panagene Inc), anti-53BP1 antibodies (Novus, NB100-304), and DAPI, as described (75, 76). Images were acquired with a Zeiss LSM 980 Confocal microscope. Quantitative analysis of 53BP1, telomeres, and their colocalization were done with Fiji/ImageJ, version 1.54h (77).

### Statistics.

Cell growth, immunoblotting, and microscopy experiments were analyzed using paired 2-tailed *t* tests, corrected for multiple comparisons where appropriate, using the false discovery rate (FDR) method, with *q* value of less than 0.05 considered statistically significant, or using ANOVA with the Šidák’s multiple-comparisons test, with adjusted *P* < 0.05 considered significant. Analysis of single-cell transcriptome analysis is detailed in Supplemental Methods; briefly, clusters were annotated with cell type and pseudobulk analysis was performed by aggregating expression values of desired cell types and performing differential expression analysis with DESeq2 (72), followed by pathway enrichment analysis using fGSEA, version 1.24.0 (73), to identify significant differences in Hallmark, C2.CP, and C8 pathways (78–80) with *P* value adjusted for FDR using Benjamini Hochberg correction considered significant at *P* < 0.05.

### Study approval.

The study was performed with approval by institutional review boards of Boston Children’s Hospital, Children’s Hospital of Philadelphia, Dana-Farber Cancer Institute, Hospital of the University of Pennsylvania, Queen Mary University of London, Texas Children’s Hospital, University of Wisconsin–Madison, and Washington University in St. Louis. Patients were recruited with institutional review board approval of participating institutions. Written informed consent was received from patients or their parents prior to participation. A subset of subjects were enrolled as a part IRB-approved retrospective chart review study with a waiver of consent, in accordance with Health and Human Services regulations.

### Data availability.

Values for all data points in graphs are reported in the Supporting Data Values file. The detailed methods and additional datasets and code utilized for this study may be found in the Supplemental Methods. Clinical sequencing datasets are not able to be publicly released due to privacy restrictions. Other questions and data requests can be directed to the corresponding authors.

## Author contributions

CMS performed IGV variant review for the custom NGS panel, clinical NGS review, clinical data collection, clinical and genetic data integration and analysis, single-cell transcriptome analysis; wrote the first manuscript draft; and acquired study funding. SC performed custom NGS panel design, IGV variant review for the custom NGS panel, somatic/germline Sanger sequencing validation, and contributed to the first manuscript draft. DVM performed scRNA-Seq bioinformatics analysis and contributed to the manuscript draft. PL performed somatic/germline Sanger validation and sequencing of Penn/CHOP cohort, TERC genotyping of Washington University patient samples, and in vitro cell growth assays, ATM target phosphorylation analysis, and β-galactosidase and TIF microscopy assays with and without ATMi. DMA and JMC performed a quantitative analysis of TIF microscopy data and contributed to the manuscript draft. TG performed patient hematopoietic colony assays, and ATM knockdown experiments in TBD fibroblasts. RJD, CF, and MZ performed in vitro growth assays and ATM target phosphorylation analysis with and without ATMi. RJD also contributed to the manuscript draft. SK performed the BrdU cell-cycle analysis assay and contributed to the manuscript draft. MB and RLB performed bioinformatic analysis of single-cell protein and DNA genotyping. SZ and BM performed IGV variant review for the custom NGS panel and somatic/germline Sanger validation. QC performed TIF microscopy. NLS performed clinical chart review and clonal architecture analysis using colony assay and Sanger sequencing. KG performed clonal architecture analysis using colony assay and Sanger sequencing. AL and AAB performed clinical chart review and somatic and germline data analysis for Baylor College of Medicine patients. RH performed bioinformatic processing and analysis of the custom NGS panel. DMT performed scRNA-Seq bioinformatics analysis. JC, JJDM, DBL, and GY performed patient sample testing and Clinical Laboratory Improvement Amendments–certified (CLIA-certified) ennSeq NGS panel analysis for somatic variants of the Penn and UK cohorts with germline variant confirmation. OS and SG provided crucial expertise and assisted with the design of single-cell DNA and protein sequencing experiments for clonal architecture, provided reagents and the MissionBio Tapestri instrument, and performed experimental work to prepare single-cell DNA and protein libraries from patient samples. JZ performed statistical review of experimental data analysis required for the manuscript revision. KSS, LGB, KNS, EFR, MP, and JEC performed clinical chart review and somatic and germline data analysis, including WES, for patients at the University of Wisconsin-Madison. PN contributed to patient regulatory clearance, patient enrollment, and management of samples for Penn/CHOP patients. AJW and ID performed clinical history review and provided samples and germline data for patients from the UK Dyskeratosis Congenita Registry. JPR performed clinical chart review and somatic and germline data analysis for the OSU patient. KAO, DCL, AEG, and LKT performed clinical chart review, sample collection and testing, and somatic and germline variant analysis for patients from Washington University. CRR, RD, and RCL performed clinical chart review, sample collection and testing, and somatic and germline variant analysis for *TERC*-mutant patients from the Dana-Farber Cancer Institute. KJB and SA performed clinical chart review and somatic and germline data analysis for the Boston Children’s patient. FBJ contributed to the experimental design of ATMi growth assays and TIF microscopy assay and their interpretation and contributed to study funding. TSO enrolled patients and oversaw patient regulatory aspects of the study at CHOP and Penn, contributed to clinical data acquisition for CHOP and Penn patients, and contributed to study funding. DVB conceptualized the study, oversaw and contributed to all aspects of the study and data analysis, enrolled patients and oversaw patient regulatory aspects of the study at Penn, wrote the first manuscript draft, and acquired study funding. All authors contributed to data analysis, participated in critical manuscript revisions, and agree with the final version of the manuscript.

## Supplementary Material

Supplemental data

Unedited blot and gel images

Supplemental table 4

Supporting data values

## Figures and Tables

**Table 1:**
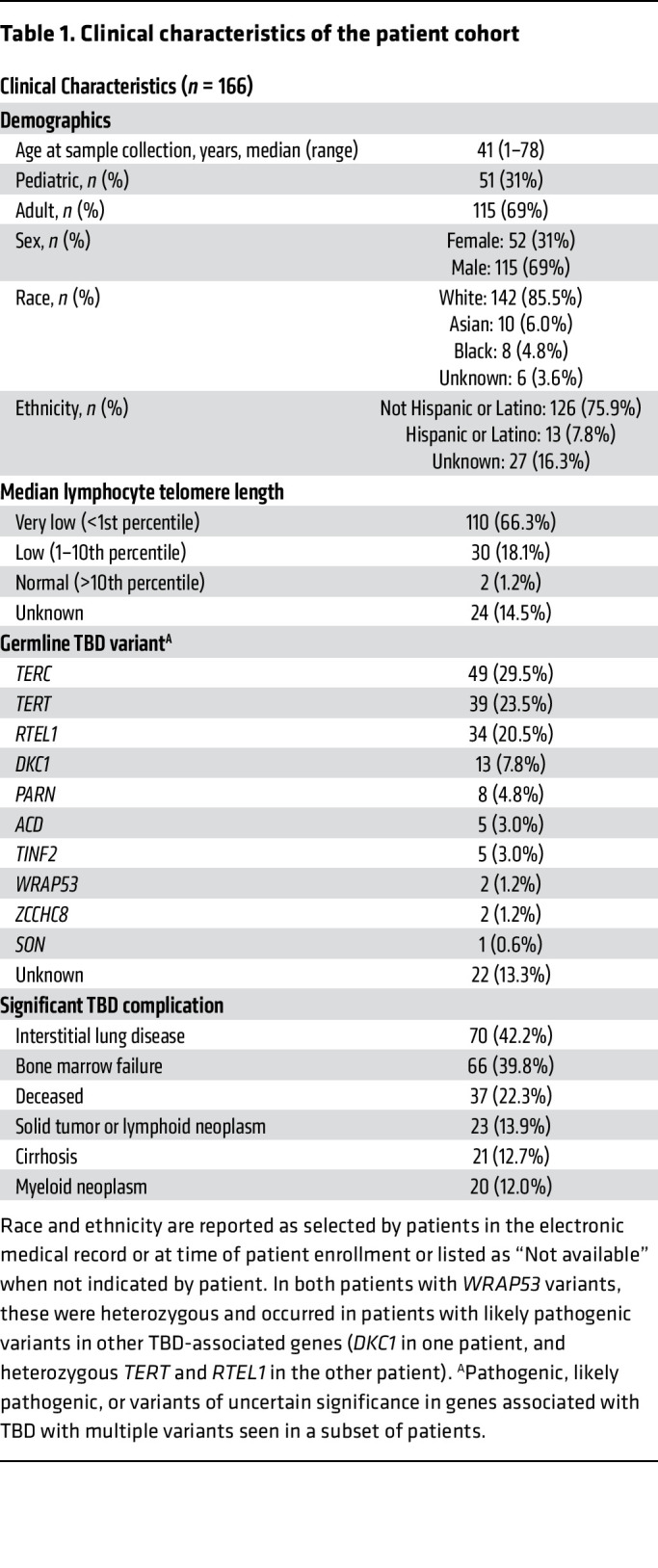
Clinical characteristics of the patient cohort

## Version 1 04/03/2025

In-Press Preview

## Version 2 04/15/2025

Electronic publication
