# Supplementary material for: ATM-dependent DNA damage response constrains cell growth and drives clonal hematopoiesis in telomere biology disorders
Source: J Clin Invest. 2025 Apr 3;135(8):e181659. doi: 10.1172/JCI181659 (PMC11996883; doi:10.1172/JCI181659)
Supplement: Supplemental data [file jci-135-181659-s295.pdf]

SUPPLEMENTAL APPENDIX to:

ATM-dependent DNA Damage Response Constrains Cell Growth and Drives Clonal Hematopoiesis in Telomere Biology Disorders

Authors: Christopher M. Sande[1-3], Stone Chen[4], Dana V. Mitchell[5], Ping Lin[4,6], Diana M. Abraham[4], Jessie Cheng[4], Talia Gebhard[4,6], Rujul J. Deolikar[4], Colby Freeman[4], Mary Zhou[4], Sushant Kumar[4], Michael Bowman[7], Robert L. Bowman[7], Shannon Zheng[4], Bolormaa Munkhbileg[4,8], Qijun Chen[1], Natasha L. Stanley[4,9], Kathy Guo[4], Ajibike Lapite[10], Ryan Hausler[4], Deanne M. Taylor[5,11], James Corines[1], Jennifer J.D. Morrissette[1], David B. Lieberman[1], Guang Yang[1], Olga Shestova[4], Saar Gill[4], Jiayin Zheng[12], Kelcy Smith-Simmer[13], Lauren G. Banaszak[13], Kyle Shoger[13], Erica F. Reinig[14], Madilynn Peterson[13], Peter Nicholas[6,15], Amanda J. Walne[16], Inderjeet Dokal[16], Justin P. Rosenheck[17], Karolyn A. Oetjen[18], Daniel C. Link[18,19], Andrew E. Gelman[18,20], Christopher R. Reilly[21], Ritika Dutta[21], R. Coleman Lindsley[21], Karyn Brundige[22], Suneet Agarwal[22], Alison A. Bertuch[10,23], Jane E. Churpek[13], Laneshia K. Tague[24], F. Brad Johnson[1], Timothy S. Olson[6,15], and Daria V. Babushok[4,8]

[1]Department of Pathology and Laboratory Medicine, Hospital of the University of Pennsylvania, Philadelphia, PA, USA

[2]Department of Laboratories, Seattle Children's Hospital, Seattle, WA, USA

[3]Department of Laboratory Medicine and Pathology, University of Washington, Seattle, WA, USA

[4]Division of Hematology-Oncology, Department of Medicine, Hospital of the University of Pennsylvania, Philadelphia, PA, USA

[5]Department of Biomedical and Health Informatics, Children's Hospital of Philadelphia, Philadelphia, PA, USA

[6]Drexel University College of Medicine, Drexel University, Philadelphia, PA, USA

[7]Department of Cancer Biology, University of Pennsylvania, Philadelphia, PA, USA

[8]Comprehensive Bone Marrow Failure Center, Children's Hospital of Philadelphia, Philadelphia, PA, USA

[9]Department of Pediatrics, Children's Hospital of Pennsylvania, Philadelphia, PA, USA

[10]Department of Pediatrics, Division of Hematology/Oncology, Baylor College of Medicine, Houston, TX, USA

[11]Department of Pediatrics, Hospital of the University of Pennsylvania, Philadelphia, PA, USA

[12] Department of Biostatistics, Epidemiology, and Informatics, University of Pennsylvania Perelman School of Medicine, Philadelphia, PA, USA and Children's Hospital of Philadelphia, Philadelphia, PA, USA

[13]Division of Hematology, Medical Oncology, and Palliative Care, Department of Medicine, University of Wisconsin-Madison, Madison, WI, USA

[14]Department of Pathology and Laboratory Medicine, University of Wisconsin-Madison, Madison, WI, USA

[15]Division of Oncology, Department of Pediatrics, Children's Hospital of Philadelphia, Philadelphia, PA, USA

[16]Blizard Institute Faculty of Medicine and Dentistry, Queen Mary University of London, London, United Kingdom

[17]Division of Pulmonary, Allergy, Critical Care and Sleep Medicine, Department of Medicine, The Ohio State University, Columbus, OH, USA

[18]Division of Oncology, Section of Stem Cell Biology, Department of Medicine, Washington University in St. Louis, St. Louis, MO, USA

[19]Department of Pathology & Immunology, Washington University in St. Louis, St. Louis, MO, USA

[20]Department of Surgery, Division of Cardiothoracic Surgery, Washington University in St. Louis, St. Louis, MO, USA

[21]Division of Hematological Malignancies, Department of Medical Oncology, Dana-Farber Cancer Institute, Boston, MA, USA

[22]Division of Hematology/Oncology, Boston Children's Hospital, Pediatric Oncology, Dana-Farber Cancer Institute, Department of Pediatrics, Harvard Medical School, Boston, MA, USA

[23]Texas Children's Cancer and Hematology Centers, Houston, TX, USA

[24]Division of Pulmonary & Critical Care Medicine, Department of Medicine, Washington University in St. Louis, St. Louis, MO, USA

Correspondence:

Christopher M. Sande, MD
Department of Laboratories, FB.4
Seattle Children's Hospital
4800 Sand Point Way NE
Seattle, WA 98105
Email: christopher.sande@seattlechildrens.org
Phone: 206-987-2103, Fax: 206-987-3840

Daria V. Babushok, MD, PhD
Division of Hematology-Oncology, Department of Medicine,
University of Pennsylvania, Philadelphia, PA
Room 808 BRB II/III, 421 Curie Blvd
Philadelphia, PA 19104
Email: daria.babushok@pennmedicine.upenn.edu
Phone: 215-573-0667, Fax: 215-615-5888

Table of Contents

Supplemental Methods 5

Telomere biology disorder (TBD) patients 5

University of Pennsylvania (Penn)/Children's Hospital of Philadelphia (CHOP) TBD cohort 5

305-gene custom next-generation sequencing panel targeting DDR, senescence, TBD and MDS/AML genes 5

Whole exome and genome sequencing (WES and WGS) 6

NGS of hematologic malignancy-associated genes 7

Sanger sequencing 7

Clonal architecture analysis 8

Telomere length measurement 8

Cytogenetic analysis and cytogenomic arrays 9

Single-cell transcriptome RNA-sequencing and analysis 10

Tapestri single cell DNA and protein sequencing 11

Chemicals 11

Cell culture, ATM inhibition and western blot 12

siRNA knockdown of ATM 12

Cell cycle analysis using 5-bromo-2'-deoxyuridine (BrdU) incorporation assay 13

Telomere dysfunction-induced foci analysis using immunohistochemistry 13

Supplemental Figures 15

Supplemental Figure S1: Sanger sequencing validation of somatic variants from custom targeted NGS. 15

Supplemental Figure S2: Longitudinal follow-up of patients with *U2AF1* and those with dup(1q) as an isolated cytogenetic abnormality. 18

Supplemental Figure S3: Co-occurrence tables indicating somatic alterations and adverse clinical outcomes by genotype 19

Supplemental Figure S4: Senescence-associated transcriptional changes in hematopoietic cells of TBD patients 20

Supplemental Figure S5: Low dose ATM inhibition selectively improves cell fitness of TBD fibroblasts. 21

Supplemental Figure S6: Low dose ATM inhibition selectively improves cell fitness of TBD fibroblasts 22

Supplemental Figure S7: Number of population doublings over the course of fibroblast culture 23

Supplemental Figure S8: Clonal architecture analysis of a patient with 4 somatic *ATM* variants (time point 1). 24

Supplemental Figure S9: Clonal architecture analysis of a patient with 4 somatic *ATM* variants after progression to MDS (time point 3) 25

Supplemental Tables 26

Supplemental Table S1: Clinical characteristics of TBD patients included in this study. 26

Supplemental Table S2: Summary of clinical findings used to establish a TBD diagnosis in subjects without a genetic confirmation of TBD or subjects for whom results of telomere length testing were unavailable. 33

Supplemental Table S3: Clonal hematopoiesis findings 38

Supplemental Table S4: Somatic *ATM* variants 52

Supplemental Table S5: *ATM* variants, somatic status confirmation, and immunosuppressive and cytotoxic therapy exposures preceding identification 54

Supplemental Table S6: Custom panel including DNA damage response, senescence, and cell cycle genes (NGS-TBD) 55

Supplemental Table S7: Clinical NGS panels 56

Supplemental Table S7a: PennSeq hematologic malignancies panel (NGS-PS) 56

Supplemental Table S7b: Penn original hematologic malignancy panel (NGS-HMP) 57

Supplemental Table S7c: CHOP comprehensive hematological cancer panel (NGS-CHOP)57

Supplemental Table S7d: Texas Children's Hospital heme DNA mutation panel, version 259

Supplemental Table S7e: Dana Farber/BWH Rapid Heme Panel 60

Supplemental Table S7f: The Ohio State James Comprehensive Hematology Genomic Panel 61

Supplemental Table S7g: Washington University error corrected NGS panel 62

Supplemental Table S8: Published 10X Genomics single cell RNA datasets of unaffected controls 63

Supplemental Table S9: Skin fibroblast cell lines used in cell growth experiments 64

Supplemental Table S10: Antibodies used for western blotting in ATM inhibition experiments 64

Supplemental Script 1: Fiji macro to analyze nuclear 53BP1 and telomere foci 65

Supplemental Script 2: Fiji macro to analyze β-galactosidase staining 67

Supplemental Script 2a: Fiji macro to count the number of nuclei based on DAPI-stained image.... 67

Supplemental Script 2b: Fiji macro to convert β-galactosidase-stained color tiff image into binary mask based on pre-specified Hue, Saturation and Brightness thresholds. 68

Supplemental Script 2c: Fiji macro to quantify staining based on converted binary mask files of color threshold-adjusted β-galactosidase-stained images. 69

Supplemental references 71

Supplemental Methods

Telomere biology disorder (TBD) patients

Patients were recruited as a part of research protocols in participating institutions with institutional review board approval. TBD was diagnosed according to standard guidelines, including documentation of low median lymphocyte telomere lengths (TL) for age in conjunction with characteristic clinical findings (e.g., bone marrow failure (BMF), pulmonary fibrosis, mucocutaneous findings) or carrying a pathogenic TBD variant (1, 2). Clinical and laboratory data were collected from the patient's medical or registry records, including basic demographics, family history, physical exam findings, and prior diagnoses and treatments. Key clinical test results for each patient included TL testing by flow-FISH, germline genetic testing, bone marrow morphology, and any somatic genetic testing on peripheral blood or bone marrow performed as part of routine clinical care, including conventional cytogenetics, cytogenomic microarray, and next-generation sequencing (NGS) studies for genes associated with hematologic malignancies.

University of Pennsylvania (Penn)/Children's Hospital of Philadelphia (CHOP) TBD cohort

The Penn/CHOP BMF cohort is a part of an a prospective/retrospective cohort for study of BMF syndromes, approved by the Institutional Review Boards (IRBs) of Penn and CHOP. Informed consent was obtained from patients or legal guardians prior to enrollment. Additional adult patients were enrolled as a part of a separate retrospective analysis at Penn, approved by the Penn IRB, where TBD patients were identified through the search of the electronic medical records (EMR) at the Hospital of the University of Pennsylvania. The search of electronic medical records was conducted by the Penn Data Analytics Center using ICD-9 and ICD-10 diagnosis codes that were associated with short telomere length syndrome; all records were then manually reviewed to confirm TBD diagnosis.

305-gene custom next-generation sequencing panel targeting DDR, senescence, TBD and MDS/AML genes.

Forty-two samples were analyzed using a 305-gene custom NGS panel targeting genes involved in DDR, cell senescence, and TBD- and MDS/AML-associated genes. A 305-gene custom NGS panel was developed

based on a review of the literature that identified genes with microRNA or protein products involved in DNA-damage response or cell senescence pathways. The panel also includes the known genes associated with TBDs as well as genes that contain known myelodysplastic syndrome (MDS)/acute myeloid leukemia (AML) somatic variants (**Supplemental Table S6**) and was designed to cover 5' untranslated regions as well as exons with 10 bp regions surrounding splice site junctions. The synthesized panel (Twist Bioscience, San Francisco, California) was performed on patient hematopoietic cell DNA, sequenced to 10,000X depth on an Illumina Hiseq2500 SBS v4 at the CHOP Center for Applied Genomics.

Sequencing data was aligned to human reference genome hg38, and variants were called using the Genome Analysis Toolkit (GATK4) Mutect2(3) and annotated with according to the Annovar and OncoKB databases(4). The results were then filtered to remove synonymous, intergenic, and deep intronic variants; variants with a population allele frequency >0.1%; variants with splicing predictors <0.5; variants with a variant allele frequency (VAF) <4%; and variants that could not be convincingly visualized in Integrative Genomics Viewer (IGV). Variants with a VAF of 0.4-0.6 were presumed to be germline. Candidate variants were prioritized for further validation based on sequence quality and manual curation, their likely germline or somatic status, and recurrence of the same variant in multiple patients.

Whole exome and genome sequencing (WES and WGS)

Comparative WES analysis of bone marrow or peripheral blood and paired constitutional DNA from 16 patients from the Penn/CHOP registry was previously described(5) and included in this study for completeness. WES analysis for 25 patients from the University of Wisconsin-Madison was performed on DNAs from peripheral blood mononuclear cells to average 125x depth of coverage at PreventionGenetics (Marshfield, WI) using their PGxome platform (https://www.preventiongenetics.com/pgxome). FASTQ files were then aligned to human reference genome hg19 using BWA (version 0.7.17-r1188), aligned BAM files were merged, read groups updated, and duplicate reads marked using picard (version 2.26.2), and base quality score recalibration performed using the GATK Toolkit (version 4.1.4.1). Variants were called using Mutect2(3) and HaplotypeCaller. Variants were annotated and filtered using VarSeq (version 2.4.0, Golden Helix, Inc, Bozeman, MT) software and a custom pipeline. In brief, filters were applied to remove variants with: a

population minor allele frequency >1%, <10 total or <5 variant reads, location in an ENCODE Blacklist region(6), location in noncoding, intergenic or deep intronic regions with the exception of specific regions known to be involved in TBDs, hereditary cancer risk, or clonal hematopoiesis (CH) (e.g., *TERT* promoter region). Variants were prioritized and interpretated based on visualization in IGV and manual curation. Variants were interpreted as somatic based on: a VAF cutoff of ≤0.3, absence of the variant in clinical or research-based testing of a germline tissue (e.g., skin fibroblasts), and/or VAF >0.3 in the expected context (e.g., *SF3B1* p.K700E at VAF 46% at the time of MDS diagnosis). Samples sequenced with WES did not have good quality coverage of the TERT promoter region.

Hematopoietic DNA for one patient was analyzed by WGS at the Center for Applied Genomics for translocation characterization. WGS library preparation used Twist Biosciences library prep kit with enzymatic fragmentation v 2.0. Sample was sequenced on NovaSeq 6000 at 30X depth on an S4 300 cycle flowcell. Translocation breakpoint was mapped by manual inspection of reads spanning the involved chromosomes in IGV, with the breakpoint mapping to LINC02226-SNX30 t(5;9)(p15.3;q32).

NGS of hematologic malignancy-associated genes

Targeted sequencing of hematologic malignancy-associated genes was performed as a part of routine clinical care or on a research basis. The CLIA-approved panel gene content used for sequencing was determined by the availability and clinical standard at the performing institution at the time of testing. Eleven genetically confirmed TBD patients from the Washington University lung transplant cohort were analyzed for CH using error-corrected sequencing with a custom targeted gene panel. Gene content of all targeted NGS panels is shown in **Supplemental Table S7**. We estimated a sensitivity of 4% for the custom NGS panel based on its performance characteristics and our requirement for subsequent Sanger validation. However, for other panels, like the error-corrected sequencing used by the Washington University group, or the sequencing performed in CLIA-certified labs on a clinical basis, the assays had higher sensitivity detailed below in **Supplemental Table S7**.

Sanger sequencing

All putative somatic variants identified by 305-gene custom targeted NGS were validated with Sanger sequencing. For all cases where paired constitutional tissue (skin or bone marrow fibroblasts), somatic versus germline status was directly confirmed by Sanger sequencing constitutional DNA. In patients where fibroblasts were not available, somatic versus germline status was verified using lymphocyte DNA from immortalized lymphoblastoid cell lines or sorted $CD3^+$ T lymphocytes.

Clonal architecture analysis

Peripheral blood mononuclear cells were cultured in Methocult H4434 Classic Medium (Stem Cell Technologies, Vancouver, Canada) for 14 days at 37°C and 5% CO2. Individual colonies were picked into 100 µL of sterile water, boiled at 99°C for 10 minutes, and crude lysate DNA was used to genotype individual colonies for presence of variants. Hematopoietic colonies for the patient 629.1, was analyzed at timepoint 1 before malignant transformation by genotyping 48 colonies for the 4 *ATM* variants using the following primers: *ATM* c.2839-1G>A, forward primer: GCACCCGGCCTATGTTTAT, reverse primer: CGATAGTGGTTTCAGAAGTTCAA; *ATM* c.6219_6256dup, forward primer: TCTCTGGTTTTCTGTTGATATCTTT, reverse primer: CATGCTGCTTGGTAATGAAGTT; *ATM* c.8495G>A, forward primer: TCTCTATTTAAAGGAGGTGCAAAAA, reverse primer: ACTGCGCGTATAAGCCAATC; *ATM* c.8672G>A, forward primer: TTGCCTTTGTAAAGTTCACATTCT, reverse primer: CCCATGCCATCCACAATATC. After malignant transformation, the same patient was analyzed by colony genotyping of 40 colonies for the 4 *ATM* variants and a newly acquired *NPM1* variant using the following primers: forward: AACTCTCTGGTGGTAGAATGAAA, reverse: TGGCAATAGAACCTGGACAA. Another patient, 629.4 was genotyped for 2 *ATM* variants in 30 colonies *ATM* c.7880A>G, forward primer: TGAAAGGCACCTAAGTCATTGAC, reverse primer: ACAGAGAGTAACACAGCAAGAA; *ATM* c.7629+1G>A, forward primer: ATGGTAGAGAGACGGAATGA, reverse primer: TCCATTTCTTAGAGGGAATGGT (colony genotyping data not shown; this is the same patient included in Figure 9).

Telomere length measurement

Telomere length measurements were performed by flow fluorescence in situ hybridization (flow FISH) as a part of clinical care by one of two CLIA TL testing centers (2-panel test at Johns Hopkins University, Baltimore, MD, or 2-panel or 6-panel tests at Repeat Diagnostics, Inc., North Vancouver, Canada). Telomere length plots were generated in R, by plotting the telomere lengths in granulocytes and lymphocytes for patients with available telomere length numerical data against the telomere lengths of healthy individuals from the previously published studies(7, 8). 1st, 10th, 50th, 90th, and 99th percentile curves were generated based on the published data for the telomere lengths in healthy controls of different ages (7, 8), by first binning the telomere length measurements in increments of 5 years, and then using a polynomial regression model of 2nd degree in R using the poly(average_bin_age, 2) function. Once the models were fitted, predictions were made for the full range of ages, generating the predicted percentile curves for each point in the sequence.

Cytogenetic analysis and cytogenomic arrays

Metaphase karyotype analysis and FISH were performed as a part of routine clinical care according to standard methods. Cytogenomic array analysis of patients' bone marrow or peripheral blood DNA, and (for experiments using ATM kinase inhibition) —patient's skin fibroblast DNA, was performed using Illumina Infinium Global Screening Arrays at the CHOP Center for Applied Genomics according to the manufacturer's protocol. Arrays were analyzed in GenomeStudio (Illumina, Inc., San Diego, CA), which allows direct visualization of B-allele frequency (BAF) and log R ratio. Chromosomal abnormalities were classified as acquired if they were clonal within the patient sample abased on the evaluation of B-allele frequency (BAF). Other abnormalities, including long tracts of homozygosity with the BAF of 1 or 0 were conservatively presumed to be constitutional. Cytogenetic abnormalities were classified as acquired when they were clonal within the patient's sample, when they newly appeared during the patient's treatment course and/or when they represented recurrent cytogenetic abnormalities (e.g., monosomy 7 or trisomy 8) in a patient with known malignancy. In cases where acquired/constitutional status of cytogenetic abnormalities was not clear, when possible, additional confirmation using phytohemagglutinin (PHA)-stimulated cytogenetic cultures was sought clinically.

Single-cell transcriptome RNA-sequencing and analysis

Peripheral blood or bone marrow mononuclear cells from three patients with genetically different TBD (autosomal dominant *TERC*, *TERT*, and familial TBD with unknown genetic defect) were tested with single-cell RNA-sequencing (scRNA-Seq), using 10X Genomics platform. For each sample, 20,000 mononuclear cells were added to the reaction to recover around 10,000 cells after gel bead in emulsion (GEM) generation. The 10,000 cells were used for cDNA and library prep using Chromium Next GEM Single Cell Gene Expression Kits v3 (10XGenomics). Sequencing was performed using Novaseq 6000 with S1 v1.5 flow cells and 100 cycle kits for a total of 20,000 reads/cell.

Sequencing data was demultiplexed, aligned, and quantified using 10x Genomics Cell Ranger 3.1.0. Data were then filtered to remove ambient mRNA contamination using SoupX(9), doublets using DoubletFinder(10), cells with features/gene counts fewer than 200 or greater than 3000, and cells with a mitochondrial gene percentage greater than 20%. Patient data was harmonized with previously published unaffected control datasets(11) **(Supplemental Table S8)** that were also processed according to the methods described above using Harmony(12). Harmonized data were then clustered at a resolution of 0.8 and using Seurat V4(13). Cell identification for each cluster was performed manually by comparing top differentially expressed genes calculated with Seurat V4(13) to defined cell clusters in Azimuth, HuBMAP Consortium Data Portal(13, 14) and CELLxGENE. After clusters were annotated with cell type, pseudobulk analysis was performed by aggregating expression values of desired cell types and performing differential expression analysis with DESeq2(15), followed by pathway enrichment analysis using fGSEA version 1.24.0(16) to identify significant differences in Hallmark, C2.CP, and C8 pathways(17-19) with the one-sided p-value, adjusted for false discovery rate using Benjamini Hochberg correction, under 0.05 considered significant. Cell cycle phase assignment was done using the CellCycleScoring() function in the R package Seurat, which classifies cells into G2M or S phase based on modular gene expression. Cells remaining without designation were manually classified as G1 phase, with the numbers of cells in different cell cycle phases for patients and controls compared with Chi-squared test, with $p<0.05$ considered significant.

Tapestri single cell DNA and protein sequencing

Clonal architecture and *ATM*-mutant contribution to hematopoietic cell lineages in a TERC-mutant patient with two *ATM* and 1 *PPM1D* variants were analyzed with single cell DNA and protein sequencing on the Tapestri platform (Mission Bio, San Francisco, CA). Flow cytometrically sorted CD45+ peripheral blood mononuclear cells were labeled using TotalSeq™-D Human Heme Oncology Cocktail, V1.0 (BioLegend, San Diego CA), and Tapestri DNA and protein sequencing libraries were prepared using Single-Cell DNA + Protein Reagent kit v3 according to the manufacturer's protocol (Mission Bio, San Francisco, CA) and sequenced at the Center of Applied Genomics at the Children's Hospital of Philadelphia. FASTQ files were analyzed using the Tapestri V3.4 DNA + Protein pipeline on the Mission Bio server. H5 files were downloaded and processed using the scDNA package in R. Samples were demultiplexed using germline SNPs(20), and merged across runs. *ATM* and *PPM1D* variants identified from bulk sequencing were selected, additional variants were identified and filtered out if they were not genotyped in less than 10% of cells or possessed an initial variant allele frequency <0.01%. Non-synonymous protein encoding variants were retained with a depth (DP) cutoff of 10, gene quality (GQ) cutoff of 20, and allele frequency cutoff of <20% for wildtype, 20-80% for heterozygous mutant and >80% for homozygous mutant calls. Cells in which GATK calls from the Tapestri pipeline passed these filters were identified, and were summarize into clones. Protein data was generated from the Tapestri pipeline as raw counts, and imported into R as a Seurat object. The protein data was logNormalized, centered and scaled on a per sample basis, and Harmony package was used to integrate and align samples. Afterwards, all principal components (45) were used as input in the KNN neighbor identification. Subsequent community identification was performed with the 'FindClusters' function with a resolution variable of 0.4. The sample of interest was then extracted from these identified clusters. Cell types were identified using 'FindAllMarkers' and manual inspection of data. The clone information was applied as metadata, and all subsequent analysis occurred in Seurat, including generation of ridgeplots, feature heatmaps, and UMAPs.

Chemicals

The ATM inhibitor (ATMi) AZD0156 (Selleck Chemicals, Houston, TX, Cat. No. S8375), dissolved in methanol, was used at a working stock of 100 µM, diluted to a final concentration of 10-160 nM in Dulbecco's phosphate-buffered saline (DPBS) (Corning, Corning, NY).

Cell culture, ATM inhibition and western blot

Primary skin fibroblasts from six patients with TBD (3 with *TERC* variants and 2 with *DKC1* variants) and four non-TBD controls **(Supplemental Table S9)** were grown in α-Minimum Essential Medium (α-MEM) (Gibco, Billings, MT) supplemented with 10% FBS, 1% Pen/Strep and 1% L-Glutamine under standard conditions in the presence or absence of ATMi AZD0156 (Selleck Chemicals, Houston, TX) at doses ranging from 10nM to 160nM. All starting cell lines were of similar passage (approximately passage 3) and were grown in log-phase and passaged when reaching ~80-85% confluence to the same cell number. At each cell passage, live cell count was obtained by counting bright cells after staining with trypan blue viability dye (Invitrogen, Waltham, MA) using hemocytometer; cell counts were done in triplicate and an average was used to generate a growth curves. All cell growth experiments were performed at least in triplicate, with data shown in **Supplemental Figures S5-S7**.

ATM pathway activation and the effect of ATM inhibition was measured by western blot of phosphorylated ATM targets (p-Chk2, pKAP1, and ATM autophosphorylation at S1981), which were compared to the levels of total Chk2, KAP1 and ATM protein, and housekeeping proteins vinculin or GAPDH as loading control (antibodies are shown in **Supplemental Table S10**). In all experiments, irradiated cells were used as a positive control for ATM induction. Protein bands were normalized to the level of a constitutively expressed housekeeping proteins to account for potential differences in gel loading and quantified using FIJI. A minimum of three independent replicate experiments were performed.

siRNA knockdown of ATM

Transient knockdown of ATM protein was performed using 10nM ON-TARGETplus-SMARTpool siRNAs to ATM (catalog #L-003201-00-0005) or the Non-Targeting Control-Pool (catalog #D-001810-10-05), both from Dharmacon (Lafayette, CO), to transfect TBD patient and control skin fibroblasts (both at passage 7) grown in log phase using Lipofectamine per manufacturer’s instructions (Invitrogen, Carlsbad, CA). Cells were collected

72 hours after transfection for western blotting and cell cycle analysis. Knockdown efficiency was determined by immunoblotting for ATM in i) untransfected cells, ii) cells treated with vehicle (lipofectamine) alone, iii) cells transfected with Non-Targeting Control-Pool siRNA, and iv) cells transfected with ATM siRNA. ATM protein band intensity was normalized to vinculin, a housekeeping protein, to account for potential differences in loading. The effect of ATM loss on cell cycle was assessed by measuring BRDU incorporation and DNA content (with 7-AAD) to determine the proportions of cells entering S, G0/G1, and G2/M phases.

Cell cycle analysis using 5-bromo-2'-deoxyuridine (BrdU) incorporation assay

Cell cycle analysis was performed using BrdU/ 7-amino-actinomycin D (7-AAD) analysis with the Phase-Flow™ FITC BrdU Kit (Cat no. 370704; BioLegend, San Diego, CA, USA) as per manufacturer's instructions. BrdU is a thymidine analog incorporated into cells during the S-phase of mitosis, while 7-AAD is a fluorescent derivative of actinomycin D that binds to GC regions of DNA. BrdU incorporation identifies cells actively replicating DNA, with 7-AAD allowing to further distinguish cells based on DNA content.

To evaluate the cell cycle profiles of TERC-mutated and control fibroblasts at baseline and after ATM kinase inhibition, control and *TERC*-mutated primary fibroblasts were grown in presence or absence of 15nM ATM inhibitor AZD0156 (Selleck Chemicals, Houston, TX, Cat. No. S8375). Cells were grown for 4-5 passages, after which they were labeled with BrdU labeling agent (5µg/ml) for 4 h. Cells were trypsinized, washed with 1x phosphate buffer saline (PBS), fixed and permeabilized. After DNase treatment for 1 h at 37°C, fixed cells were stained with FITC labelled anti-BrdU antibody for 20 minutes at room temperature. After washing, cells were resuspended in staining buffer (2% fetal bovine serum in PBS) containing 7-AAD (1 µg/sample) and incubated for 10 minutes at room temperature in the dark. BrdU labeling and 7-AAD staining were detected using BD Accuri™ C6 plus flow cytometer. Flow cytometry data were analyzed using FlowJo_v10.9.0. The experiment was performed twice, each time with three technical replicates.

Telomere dysfunction-induced foci analysis using immunohistochemistry

Early passage fibroblasts grown at log-phase at ~70% confluence with and without ATMi were replated onto glass slides and grown overnight. Cells were then stained using a combination of 50µg/ml Cy3-conjugated

peptide nucleic acid (PNA) probe to the telomere repeat TTAGGG (Panagene Inc, Korea), anti-53BP1 antibodies (Novus, NB100-304), and DAPI, as previously described(21, 22).

Confocal images were obtained with a Zeiss LSM 980 confocal inverted microscope. Images were acquired at 40x magnification. All lasers were kept at constant intensity to capture all images. Quantitative image analysis was performed in Fiji(23) (see **Supplemental Script 1** below for macro code). The DAPI images were used to define the nuclear area in which to measure the number of telomere spots (data not shown) and 53BP1 spots and their area. Images were first deconvoluted, and nuclear outlines of interest were drawn and applied as a mask onto the 53BP1 and telomere-stained images. Statistical analyses of differences in number and area of 53BP1 and telomere area between samples were performed using ANOVA. Co-localization between 53BP1 and telomere staining was quantified on each nucleus using the “AND” function in Fiji. Nuclei with and without co-localization were compared between samples as a binary outcome using Fisher’s exact tests, with one-sided p-value <0.05 considered significant.

Supplemental Figures

Supplemental Figure S1: Sanger sequencing validation of somatic variants from custom targeted NGS.

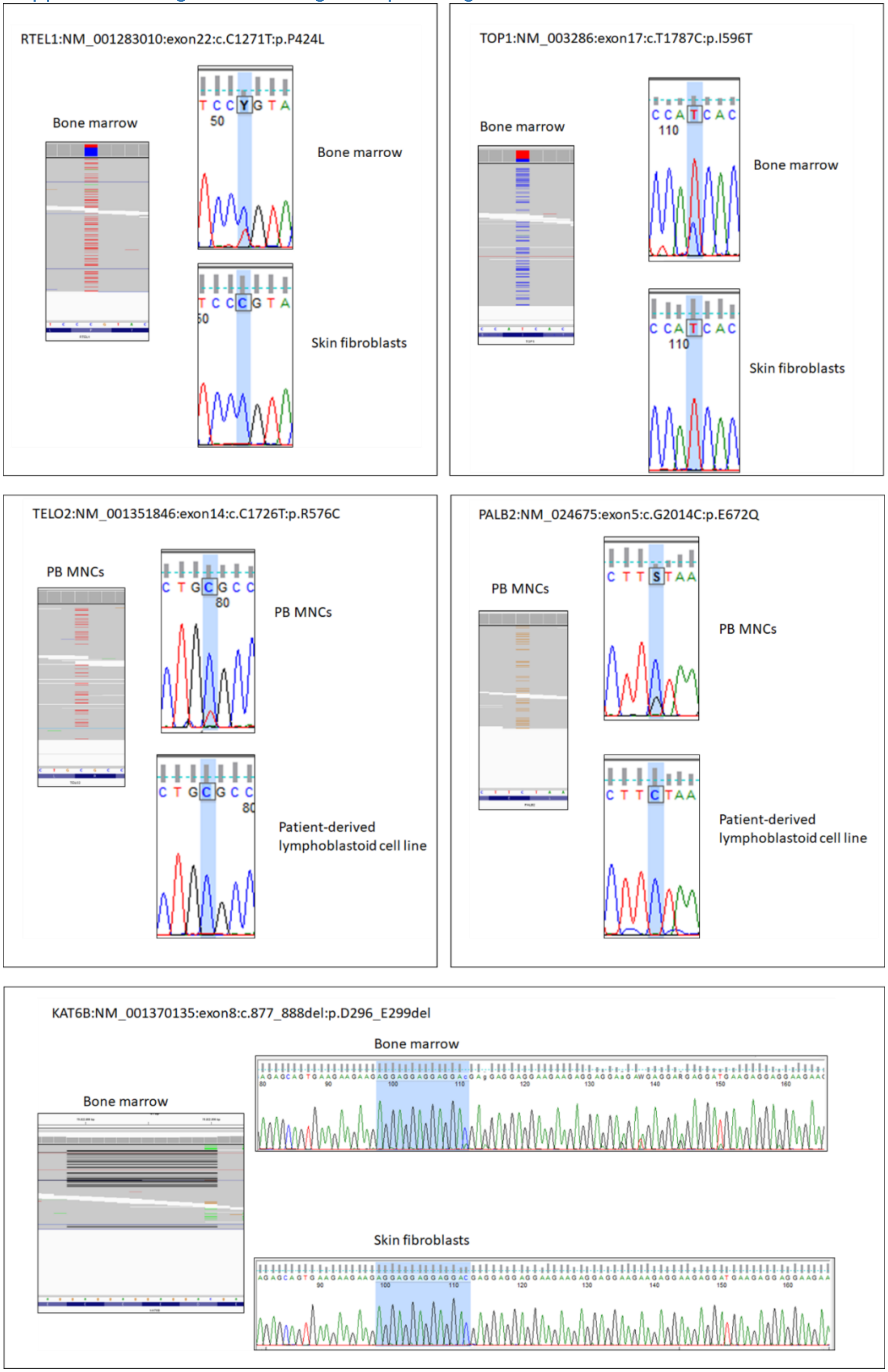

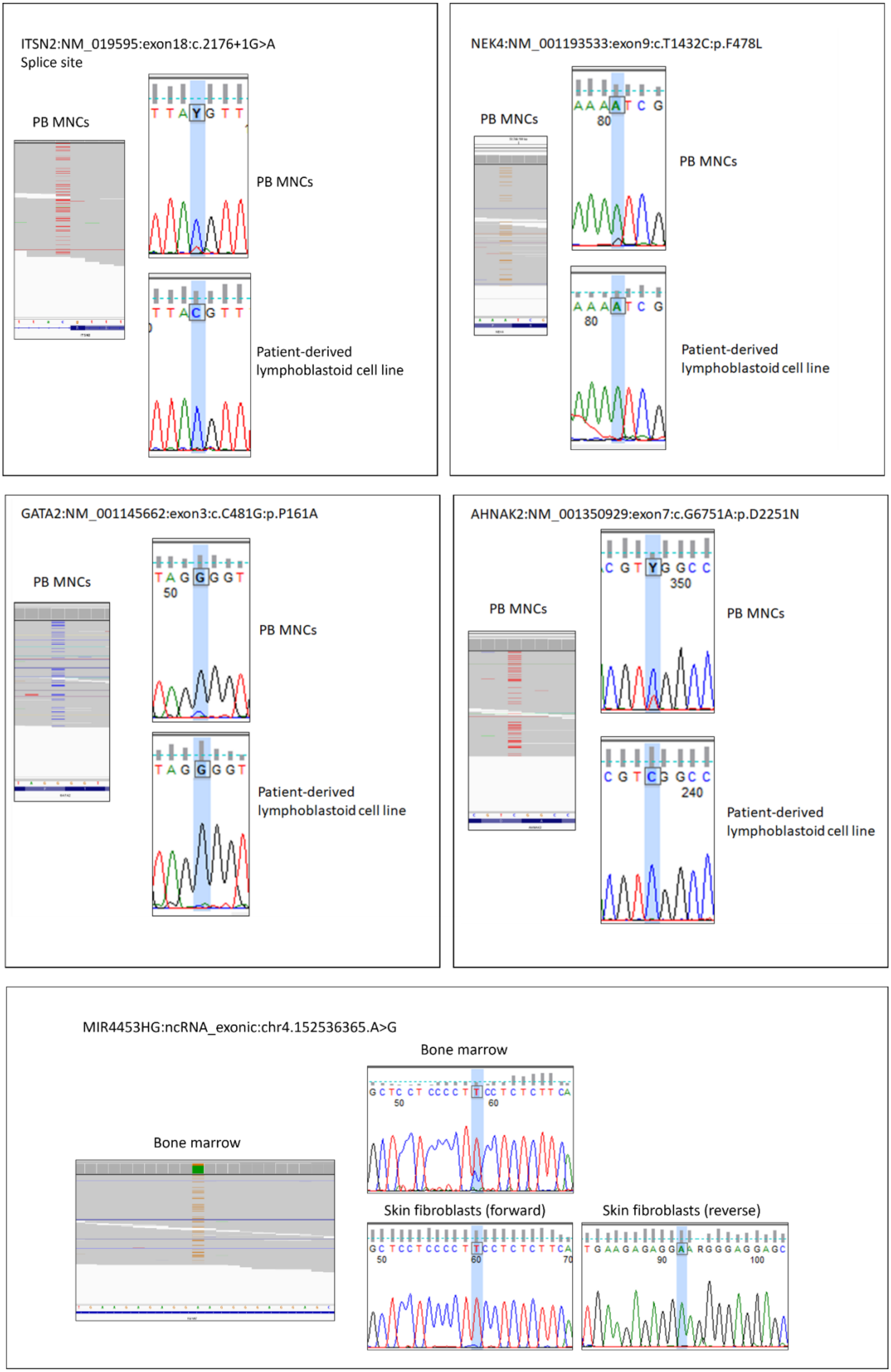
ITSN2:NM_019595:exon18:c.2176+1G>A
Splice site
PB MNCs
PB MNCs
Patient-derived lymphoblastoid cell line
NEK4:NM_001193533:exon9:c.T1432C:p.F478L
PB MNCs
PB MNCs
Patient-derived lymphoblastoid cell line
GATA2:NM_001145662:exon3:c.C481G:p.P161A
PB MNCs
PB MNCs
Patient-derived lymphoblastoid cell line
AHNAK2:NM_001350929:exon7:c.G6751A:p.D2251N
PB MNCs
PB MNCs
Patient-derived lymphoblastoid cell line
MIR4453HG:ncRNA_exonic:chr4.152536365.A>G
Bone marrow
Bone marrow
Skin fibroblasts (forward)
Skin fibroblasts (reverse)

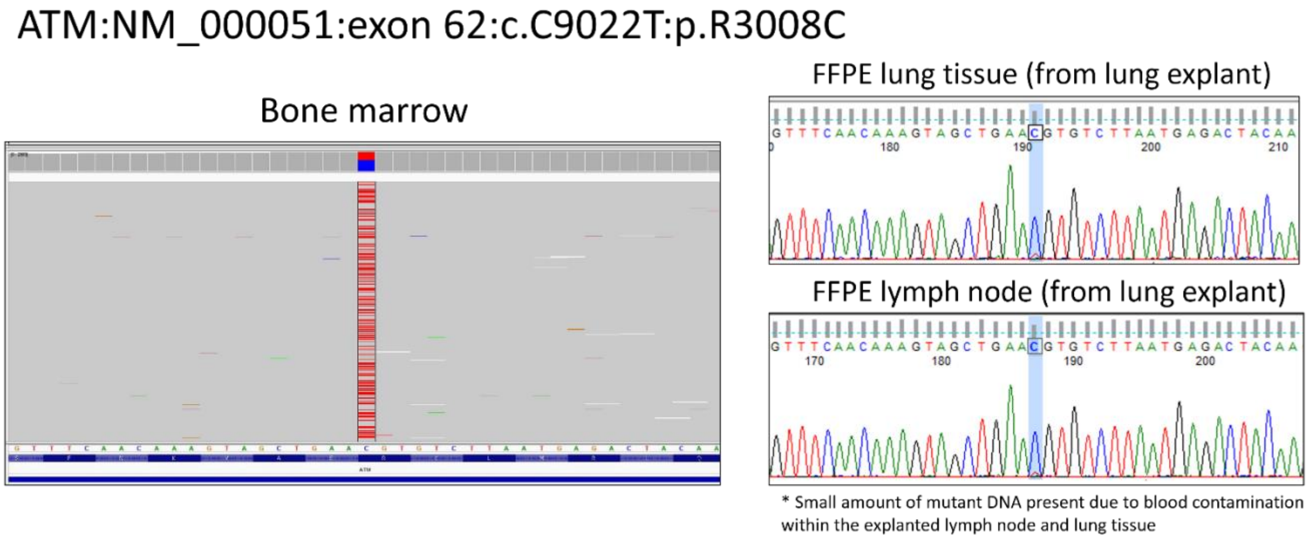

Validation of putative somatic variants detected by custom targeted NGS. For each variant, the left-hand panel show an IGV screenshot of NGS of bone marrow or peripheral blood mononuclear cells (PB MNCs) showing a somatic variant. On the right-hand side of the panel, shown are the Sanger sequencing chromatographs demonstrating the variant in hematopoietic DNA but absent in the paired constitutional tissue DNA. The bottom variant (*ATM* p.R3008C) was identified at high variant allele fraction in the bone marrow DNA and was subsequently confirmed to be somatic using the formalin-fixed, paraffin-embedded (FFPE) lung explant tissue, with the peak corresponding to the mutant residue explained by blood contamination present within the explant tissue. Abbreviations in the chromatographs: Y = C or T (pyrimidine); S = G or C.

Supplemental Figure S2: Longitudinal follow-up of patients with *U2AF1* and those with dup(1q) as an isolated cytogenetic abnormality.

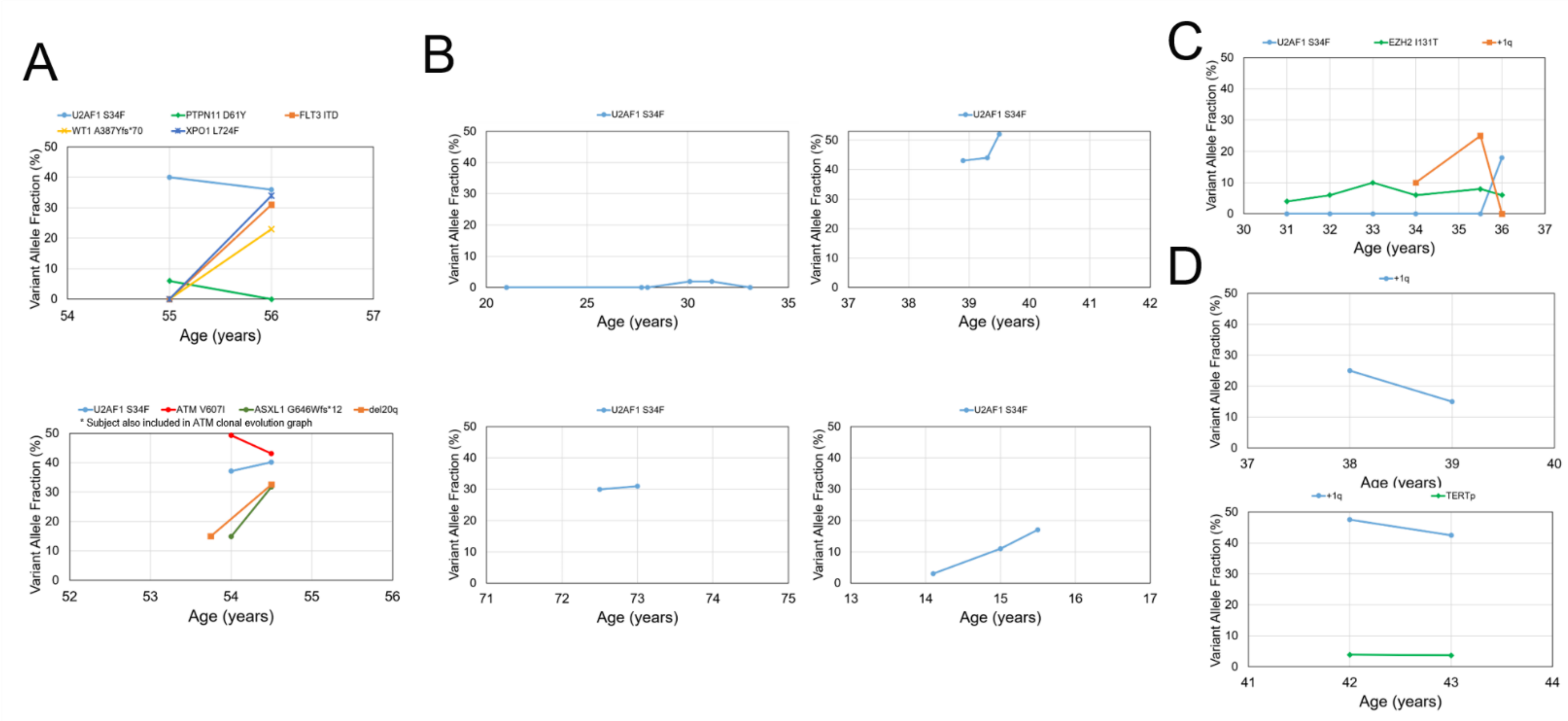

A) Trends of variant allele fractions (VAFs) for 2 TBD patients with the *U2AF1* p.S34F variant who were diagnosed with myeloid malignancies. Both patients had multiple variants associated with myeloid neoplasms. Patient in the bottom left graphic also has an *ATM* variant and is included in Figure 8 in the main manuscript. B and C) Show VAF trends for 5 TBD patients with the *U2AF1* p.S34F variant without malignant transformation. The patient in C) also had a transient cytogenetic abnormality dup(1q). Depicted in D) are the longitudinal follow-up VAF trends for patients with dup(1q) as their sole cytogenetic abnormality without malignant transformation. For plotting VAF for cytogenetic abnormalities, autosomal cytogenetic frequencies were halved to be comparable to the VAF for somatic variants.

Supplemental Figure S3: Co-occurrence tables indicating somatic alterations and adverse clinical outcomes by genotype.

A

	ACD	*DKC1*	*PARN*	*RTEL1*	*SON*	*TERC*	*TERT*	*TINF2*	*WRAP53*	*ZCCHC8*	Multiple	Unknown	Somatic *ATM* variant	No *ATM* variant
Any clonal hematopoiesis	2	4	4	12	0	32	17	1	0	2	10	10	12	67
Any chromosomal abnormality	0	2	2	3	0	11	2	0	0	1	3	5	3	21
Karyotype abnormality	0	2	2	3	0	6	2	0	0	1	3	5	3	16
cnLOH	0	0	0	0	0	5	0	0	0	0	0	0	0	5
Gain of 1q	0	0	1	1	0	2	1	0	0	1	1	2	1	6
Complex karyotype	0	1	0	1	0	1	1	0	0	0	1	0	0	3
Any sequence variant	2	3	2	10	0	24	15	1	0	2	6	7	12	50
Any DDR variant	2	1	1	3	0	17	5	0	0	2	3	3	12	19
ATM	0	0	0	1	0	7	1	0	0	2	1	2	12	0
TP53	0	1	0	1	0	4	1	0	0	0	1	1	2	5
PPM1D	1	0	1	1	0	5	3	0	0	1	1	0	2	9
Telomere maintenance gene	1	0	0	2	0	4	3	1	0	0	0	0	0	11
Reversion of germline variant	0	1	0	0	0	5	0	0	0	0	0	0	0	6
TERT promoter variant	0	0	0	2	0	2	3	0	0	0	0	0	0	7
Any MDS/AML-associated variant	1	2	0	6	0	11	8	0	0	0	3	5	5	26
Splicing factor variant	0	1	0	3	0	5	3	0	0	0	1	3	1	14
U2AF1	0	1	0	3	0	4	2	0	0	0	1	2	1	11
ASXL1	0	0	0	0	0	2	1	0	0	0	0	2	1	4
TET2	0	0	0	2	0	2	1	0	0	0	1	0	1	3
DNMT3A	1	0	0	0	0	1	1	0	0	0	0	0	0	3
SF3B1	0	0	0	0	0	0	1	0	0	0	0	1	0	2
GATA2	0	0	0	0	0	1	0	0	0	0	0	0	0	1
Bone marrow failure	2	11	2	12	0	24	10	4	1	2	14	7	4	63
Cirrhosis	0	1	1	4	0	6	7	0	0	1	2	2	3	17
Interstitial lung disease	3	1	5	19	0	15	20	1	0	2	9	10	7	63
Myeloid neoplasm	0	2	1	4	0	9	3	0	0	0	3	3	3	17
Solid tumor	0	6	2	4	0	4	3	0	0	0	4	7	1	22
Deceased	2	6	0	7	0	8	9	2	0	1	5	4	4	33

Number of patients with the indicated findings

B

	ACD	*DKC1*	*PARN*	*RTEL1*	*SON*	*TERC*	*TERT*	*TINF2*	*WRAP53*	*ZCCHC8*	Multiple	Unknown	Somatic *ATM* variant	No *ATM* variant
Any clonal hematopoiesis	5	13	8	34	1	49	39	5	2	2	21	22	12	129
Any chromosomal abnormality	3	11	5	24	1	34	28	5	2	1	16	16	9	88
Karyotype abnormality	0	11	5	24	1	34	28	5	2	1	16	16	9	88
cnLOH	1	5	1	5	1	23	10	1	1	1	5	6	3	47
Gain of 1q	3	3	20	24	1	34	28	5	2	1	16	16	9	88
Complex karyotype	3	11	5	24	1	34	28	5	2	1	16	16	9	88
Any sequence variant	4	11	8	30	1	46	32	3	2	2	16	18	12	129
Any DDR variant	4	11	8	30	1	46	32	3	2	2	16	18	12	129
ATM	4	11	7	30	1	45	32	3	2	2	16	17	12	129
TP53	4	11	8	30	1	46	32	3	2	2	16	18	12	129
PPM1D	4	10	8	29	n/a	46	32	3	2	2	15	16	12	127
Telomere maintenance gene	4	11	8	30	1	46	32	3	2	2	16	19	12	129
Reversion of germline variant	3	7	6	21	n/a	30	30	1	1	1	12	21	6	102
TERT promoter variant	3	11	2	17	1	44	24	4	2	2	12	13	10	94
Any MDS/AML-associated variant	4	11	8	30	1	46	32	3	2	2	16	18	12	129
Splicing factor variant	4	11	8	30	1	46	32	3	2	2	16	18	12	129
U2AF1	4	11	8	30	1	46	32	3	2	2	16	18	12	129
ASXL1	4	11	8	30	1	46	32	3	2	2	16	18	12	129
TET2	4	11	8	30	1	46	32	3	2	2	16	18	12	129
DNMT3A	4	11	8	30	1	46	32	3	2	2	16	18	12	129
SF3B1	4	11	8	30	1	46	32	3	2	2	16	18	12	129
GATA2	4	11	8	30	1	46	32	3	2	2	16	18	12	129
Bone marrow failure	5	13	8	34	1	49	39	5	2	2	21	22	12	129
Cirrhosis	5	13	8	34	1	49	39	5	2	2	21	22	12	129
Interstitial lung disease	5	13	8	34	1	49	39	5	2	2	21	22	12	129
Myeloid neoplasm	5	13	8	34	1	49	39	5	2	2	21	22	12	129
Solid tumor	5	13	8	34	1	49	39	5	2	2	21	22	12	129
Deceased	5	13	8	34	1	49	39	5	2	2	21	22	12	129

Evaluable patients per category (n<5 shown in gray)

The table of somatic variant and clinical feature co-occurrence includes all patients in this study. Patients with multiple germline variants were included in both the individual gene groups and in the multiple mutation group. The number of patients with a specific feature co-occurrence (e.g. somatic mutation occurring in patients with germline *ACD*) are shown in (A), with the number of patients evaluated per each category (i.e. the denominator) is shown in (B). Gray shading in (B) indicates categories where the number of analyzed samples was less than 5.

Supplemental Figure S4: Senescence-associated transcriptional changes in hematopoietic cells of TBD patients

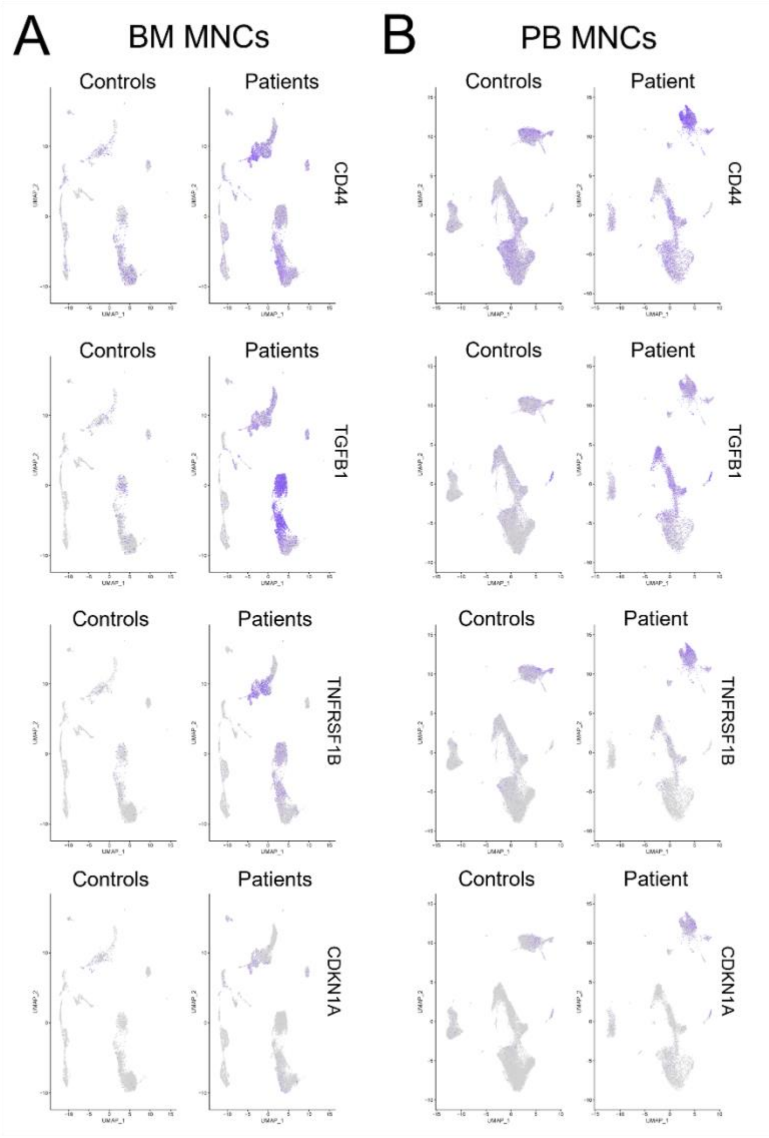

Single-cell transcriptome analysis of hematopoietic cells in TBD patients compared to healthy controls. (A) UMAP plot of bone marrow mononuclear cells (BM MNCs) from 2 healthy controls and 2 patients with TBD, and (B) peripheral blood mononuclear cells (PB MNCs) from 4 healthy controls and 1 patient with TBD, showing significant upregulation of senescence-associated transcripts in TBD patients. In BM MNCs, senescence associated transcript *CD44* was significantly upregulated within in T cells, mature and precursor B cells, monocytes, erythroid precursors; *TGFB1* was significantly upregulated in T cells, mature and precursor B cells, NK cells, and plasmacytoid dendritic cells; *TNFRSF1B* was significantly upregulated in T cells, granulocytes, monocytes, NK cells; and *CDKN1A* was significantly upregulated in HSPCs and T cells. In PB MNCs, *CD44* was significantly upregulated in T cells, and monocytes; *TGFB1* was significantly upregulated in T cells, B cells, NK cells, dendritic cells, and monocytes; *TNFRSF1B* was significantly upregulated in monocytes, and *CDK1A* was significantly upregulated in monocytes. In all cases, p-value adjusted for multiple comparisons using the false discovery rate method with Benjamini Hochberg correction was <0.05.

Supplemental Figure S5: Low dose ATM inhibition selectively improves cell fitness of TBD fibroblasts.

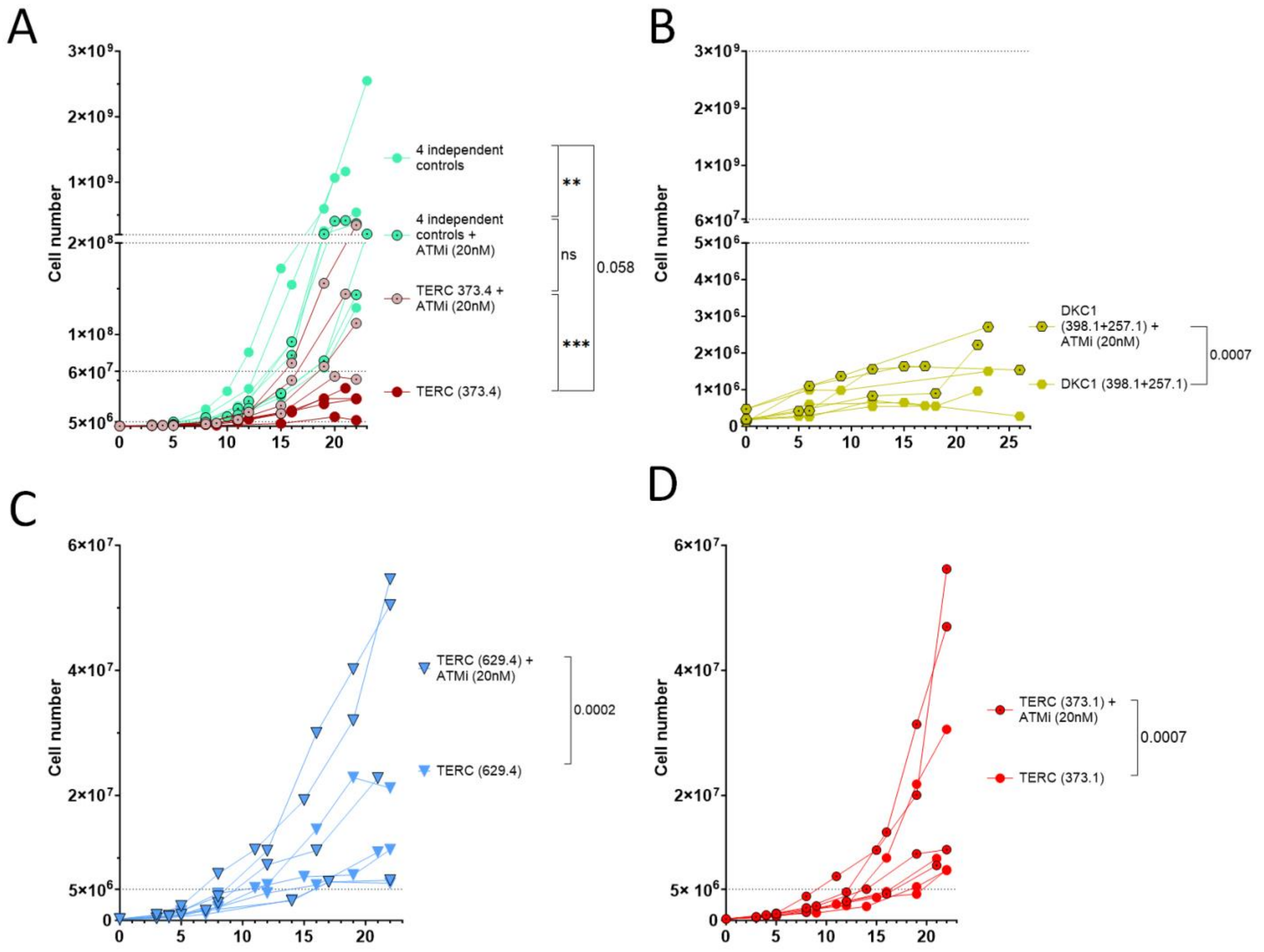

Primary skin fibroblasts from patients with TBD (3 with *TERC* variants and 2 with *DKC1* variants) and four non-TBD controls were grown in log phase under standard conditions in the presence or absence of 20nM ATMi AZD0156. Shown are growth curves from replicate experiments. ATMi reduced growth of control cells (A), but significantly improved growth of TERC-mutated fibroblasts (A, C, D) and DKC1-mutated fibroblasts (B).

Supplemental Figure S6: Low dose ATM inhibition selectively improves cell fitness of TBD fibroblasts

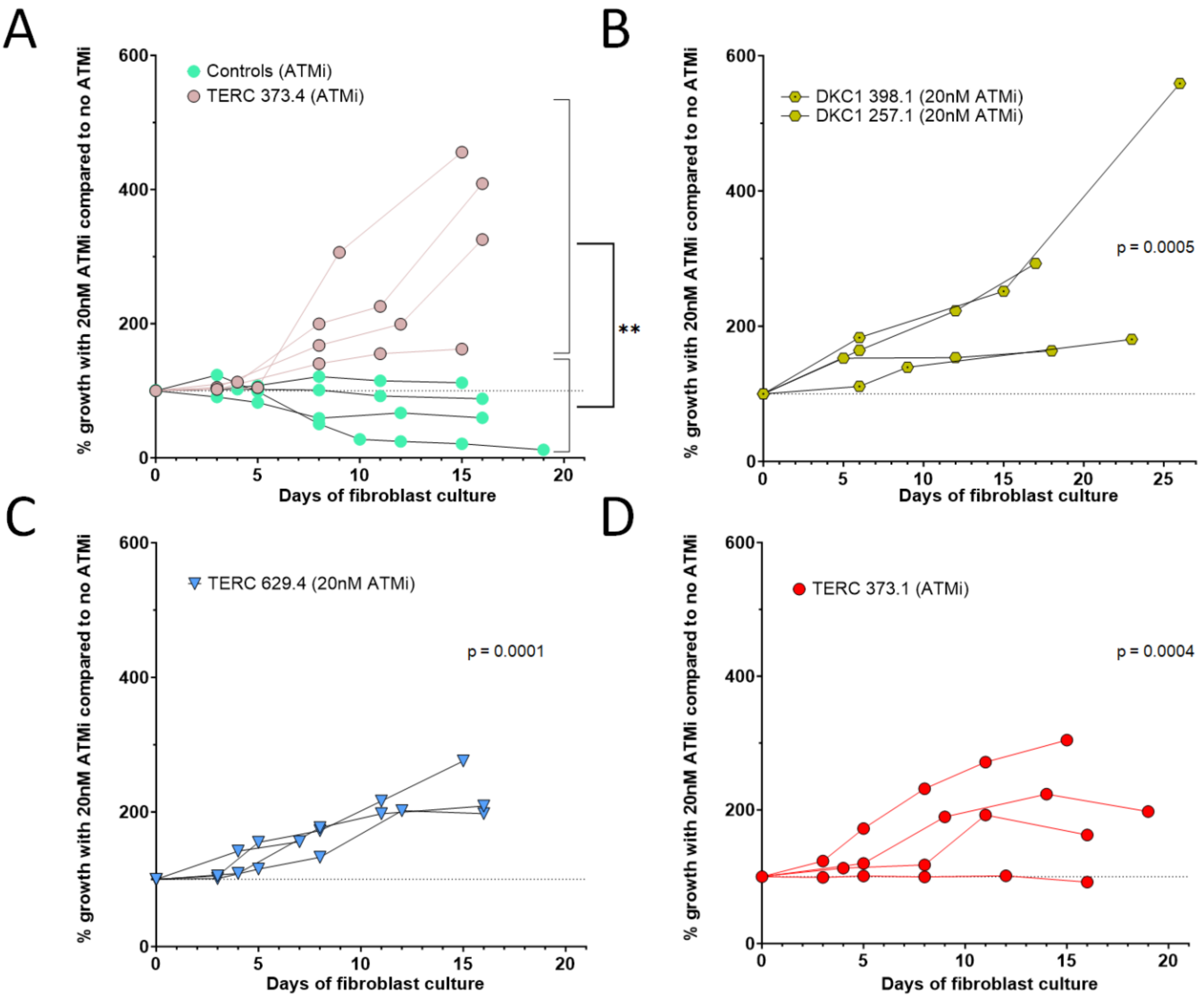

Shown are the data from Supplemental Fig S4, now showing growth rate with ATMi normalized as a % of growth without ATMi. While 20nM ATMi reduced growth of control cells (A), growth of all TBD cell lines was significantly improved by ATMi. In each sub-plot, different lines represent different biological replicates (n=4).

Supplemental Figure S7: Number of population doublings over the course of fibroblast culture

Non-TBD controls	Control (43.1)		Control (58.1)		Control (370.1)		Control (489.1)	
	Passage at start (P3)		Passage at start (P3)		Passage at start (P3)		Passage at start (P3)	
	Media only	20nM ATMi	Media only	20nM ATMi	Media only	20nM ATMi	Media only	20nM ATMi
Cells at start of culture	249750	249750	249700	249700	250000	250000	250000	250000
Cumulative cells at end of culture	540320105	371000000	129234774	143499582	1165312379	416226403	2549836310	211221122
Population doublings	11.1	10.5	9.0	9.2	12.2	10.7	13.3	9.7
Difference in doublings (with-without AMTi)		↓ -0.5		→ 0.2		↓ -1.5		↓ -3.6
Days in culture	22	22	22	22	21	21	23	23

TERC-mutated TBD	Replicate 1 (373.1)		Replicate 2 (373.1)		Replicate 3 (373.1)		Replicate 4 (373.1)	
	Passage at start (P3)		Passage at start (P3)		Passage at start (P3)		Passage at start (P3)	
	Media only	20nM ATMi	Media only	20nM ATMi	Media only	20nM ATMi	Media only	20nM ATMi
Cells at start of culture	250000	250000	249275	249275	250100	250100	250000	250000
Cumulative cells at end of culture	9766840	8381300	8122643	56217322	30583933	46998019	8014079	11335462
Population doublings	5.3	5.1	5.0	7.8	6.9	7.6	5.0	5.5
Difference in doublings (with-without AMTi)		→ -0.2		↑ 2.8		↑ 0.6		↑ 0.5
Days in culture	22	22	22	22	22	22	22	22

TERC-mutated TBD	Replicate 1 (373.4)		Replicate 2 (373.4)		Replicate 3 (373.4)		Replicate 4 (373.4)	
	Passage at start (P3)		Passage at start (P3)		Passage at start (P3)		Passage at start (P3)	
	Media only	20nM ATMi	Media only	20nM ATMi	Media only	20nM ATMi	Media only	20nM ATMi
Cells at start of culture	250000	250000	250250	250250	250750	250750	250000	250000
Cumulative cells at end of culture	29970817	153483224	29661111	112422841	30385974	345000000	6670575	51209279
Population doublings	6.9	9.3	6.9	8.8	6.9	10.4	4.7	7.7
Difference in doublings (with-without AMTi)		↑ 2.4		↑ 1.9		↑ 3.5		↑ 2.9
Days in culture	22	22	22	22	22	22	22	22

TERC-mutated TBD	Replicate 1 (629.4)		Replicate 2 (629.4)		Replicate 3 (629.4)		Replicate 4 (629.4)	
	Passage at start (P2-P3)		Passage at start (P2-P3)		Passage at start (P2-P3)		Passage at start (P2-P3)	
	Media only	20nM ATMi	Media only	20nM ATMi	Media only	20nM ATMi	Media only	20nM ATMi
Cells at start of culture	220000	220000	249600	249600	252000	252000	250000	250000
Cumulative cells at end of culture	8327532	16701451	11331261	54582265	21186025	50450965	5978484	6430260
Population doublings	5.2	6.2	5.5	7.8	6.4	7.6	4.6	4.7
Difference in doublings (with-without AMTi)		↑ 1.0		↑ 2.3		↑ 1.3		→ 0.1
Days in culture	22	22	22	22	22	22	22	22

DKC1-mutated TBD	Replicate 1 (398.1)		Replicate 2 (398.1)		Replicate 1 (257.1)		Replicate 2 (257.1)	
	Passage at start (P3)		Passage at start (P3)		Passage at start (P3)		Passage at start (P3)	
	Media only	20nM ATMi	Media only	20nM ATMi	Media only	20nM ATMi	Media only	20nM ATMi
Cells at start of culture	472500	472500	123500	123500	180000	195000	180000	195000
Cumulative cells at end of culture	1500000	2710027	1022281	6890632	560000	1640000	960000	2220000
Population doublings	1.7	2.5	3.0	5.8	1.6	3.1	2.4	3.5
Difference in doublings (with - without AMTi)		↑ 0.9		↑ 2.8		↑ 1.4		↑ 1.1
Days in culture	23	23	33	33	17	17	22	22

Low passage (approximately passage 3 after the fibroblast line establishment) primary skin fibroblasts from patients with TBD and four non-TBD controls were grown in log phase under standard conditions in the presence or absence of 20nM ATMi AZD0156. Control fibroblasts were growing normally and were not senescent, while TBD fibroblasts were already approaching senescence at the start of culture. Shown are the corresponding data on cell population doublings over the course of culture with and without 20nM ATMi (shaded from yellow to progressively darker green with increasing doubling number). The differences in doubling number between the corresponding ATMi and no ATMi conditions for each growth experiment are indicated visually with an upward green arrow for >0.25 more population doublings with ATMi, and with a downward red arrow for a >0.25 fewer cell population doublings with ATMi.

Supplemental Figure S8: Clonal architecture analysis of a patient with 4 somatic *ATM* variants (time point 1).

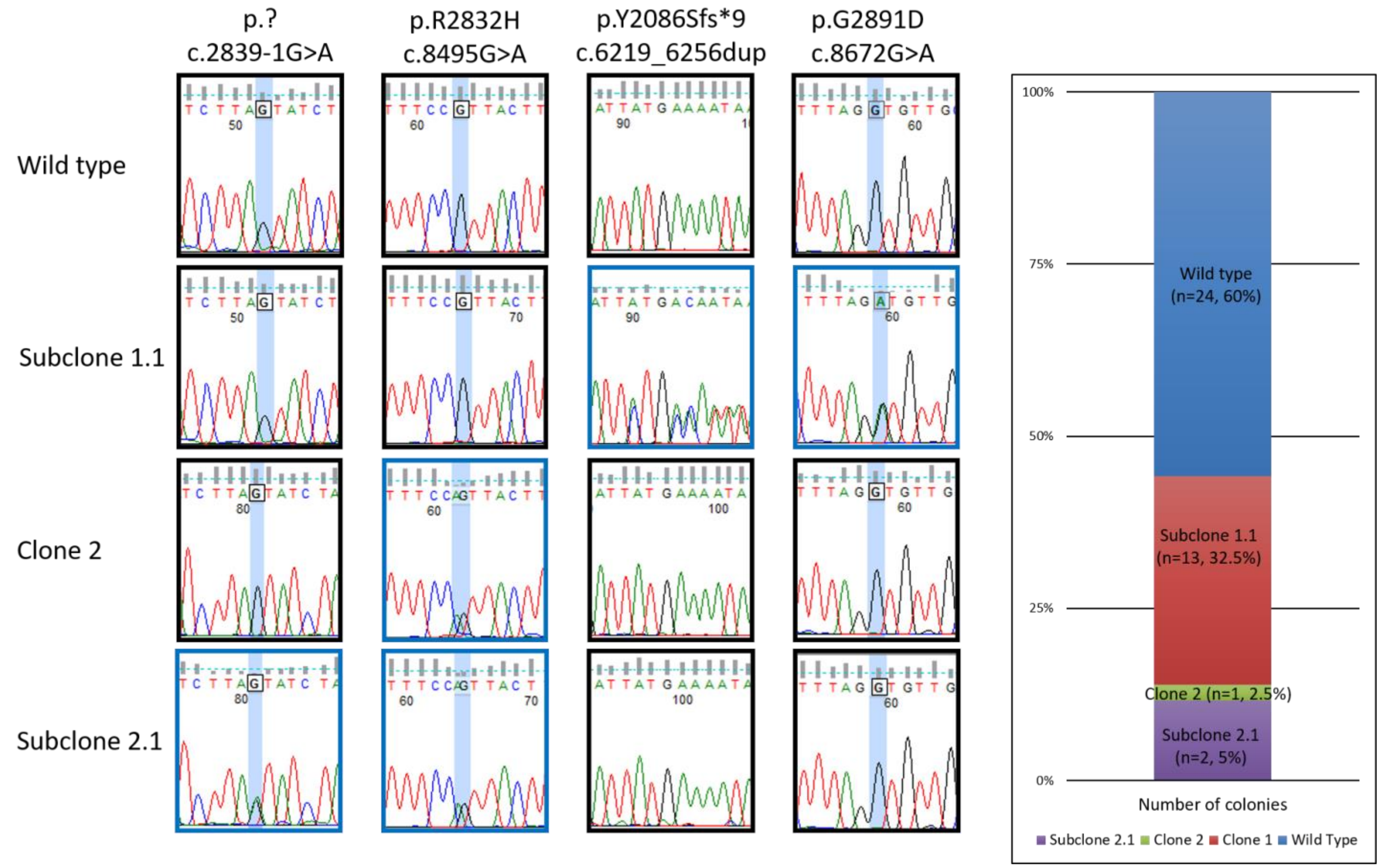

Shown is the clonal architecture analysis of a patient with four loss-of-function somatic *ATM* variants. Single cell colony genotyping using Sanger sequencing showed that *ATM* variants occurred initially as heterozygous variants in 2 independent clones, subsequently acquiring two subclonal *ATM* variants (these are presumed to be in trans). On the left of the figure, shown are the Sanger sequencing chromatographs corresponding to each of the clone types (Wild type, Subclone 1.1, Clone 2, Subclone 2.1), with the corresponding genotyping results for the 4 *ATM* variants shown horizontally across the row. Blue outline is used to highlight the presence of the variant. On the right of the figure, the relative abundance of each clone is shown using a stacked bar plot.

Supplemental Figure S9: Clonal architecture analysis of a patient with 4 somatic *ATM* variants after progression to MDS (time point 3)

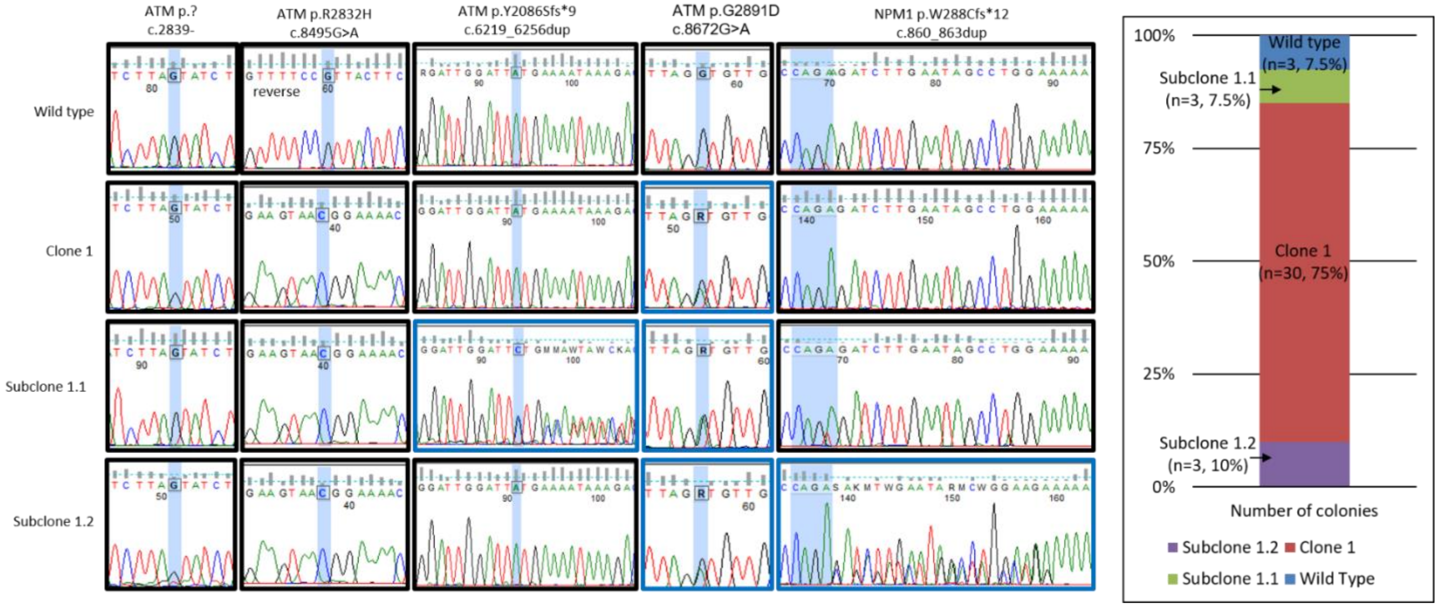

Single cell colony genotyping of 4 *ATM* variants and an *NPM1* variant using Sanger sequencing after MDS progression showed that *NPM1* variant occurred in a subclone 1.2, combining a heterozygous *ATM* p.G2891D variant with NPM1 p.W288Cfs*12 variant. On the left of the figure, shown are the Sanger sequencing chromatographs corresponding to each of the clone types, with the corresponding genotyping results for the 4 *ATM* and *NPM1* variants shown horizontally across the row. Blue outline is used to highlight the presence of the variant. On the right of the figure, the relative abundance of each clone is shown using a stacked bar plot.

Supplemental Tables

Supplemental Table S1: Clinical characteristics of TBD patients included in this study.

Patient ID	Age	Sex	Race/ Ethnicity	Mucocutanteous Features	Early Graying/Gray Forelock	Luminal Webbing or Stenosis	Joint Dysplasia or Avascular Necrosis	Osteopenia, Osteoporosis	Immunodeficiency	Endocrine Dysfunction	Cytopenias	Bone Marrow Failure	Cirrhosis	Lung Disease	Myeloid Neoplasm	Solid Tumor and Other Neoplasms	Transplant	Deceased	Family History	Telomere Length - Lymphocytes	Telomere Length - Granulocytes	Germline Variant	Evidence of Clonal Hematopoiesis
7569-009.01	16	M	Hispanic	Y		E		Y			Y	Y							Y	VL	n/a	*TERT* (VUS x2)	Y
7569-012.01	13	M	White					Y			Y	Y							Y	VL	VL	Unknown	N
7569-012.02	40	F	White																Y	VL	n/a	Unknown	Y
7569-023.01 (WUSTL 320.01)	53	M	White	Y							Y	Y				oSCC			N	VL	n/a	*DKC1*	Y
7569-047.01	12	M	White	Y	T	E					Y	Y							Y	n/a	n/a	*RTEL1* x2	N
7569-047.04	5	M	White								Y	Y							Y	n/a	n/a	*RTEL1*	N
7569-060.01	20	M	White	Y	WF						Y								Y	n/a	n/a	*TERC*	Y
7569-060.02	54	F	White				Y	Y			Y	Y		Y	MDS	cSCC, cBCC			Y	VL	n/a	*TERC*	Y
7569-103.03	20	F	White																Y	VL	n/a	*TERC*	N
7569-103.04	32	F	White																Y	VL	n/a	*TERC*	Y
7569-103.05	17	F	White	Y															Y	VL	n/a	*TERC*	N
7569-103.09	29	F	White																Y	VL	n/a	*TERC*	Y
7569-103.10	25	M	White																Y	n/a	n/a	*TERC*	N
7569-103.15	33	M	White																Y	n/a	n/a	*TERC*	N
7569-109.01	31	M	White	Y	GF	U		Y			Y	Y	Y	Y			BM	31	Y	n/a	n/a	*TERT*	N
7569-134.03 (WUSTL 141.03)	24	M	White													CRC		24	Y	VL	n/a	*DKC1*	N

7569-146.01	11	F	Unknown								Y	Y					BM		Y	VL	n/a	*TERT* x2	Y
7569-199.01	16	M	White	Y		E, U		Y			Y	Y							N	n/a	n/a	*DKC1*	N
7569-257.01	17	M	White	Y		E		Y	Y		Y	Y				HA*	BM	17	N	VL	VL	*DKC1*	N
7569-328.01	11	M	Hispanic	Y				Y	Y	Y	Y						Lu	18	N	VL	L	*DKC1* (VUS)	N
7569-373.01	14	M	White	Y							Y	Y							Y	VL	VL	*TERC*	N
7569-373.02	41	F	White	Y															Y	VL	VL	*TERC*	Y
7569-373.04	10	F	White	Y							Y								Y	VL	VL	*TERC*	Y
7569-398.01	21	M	White	Y		E, U, LD					Y	Y	Y						Y	VL	VL	*DKC1*	Y
7569-522.01	35	M	White	Y	EG	U	Y	Y			Y	Y	Y				Li		Y	VL	VL	*TERT* (VUS)	Y
7569-529.01	16	M	Hispanic								Y								Y	L	L	*RTEL1*	N
7569-534.01	17	M	White	Y												oSCC			N	VL	L	Unknown	N
7569-587.01	3	F	White	Y															Y	L	NSQ	*TERT* (VUS)	N
7569-596.01	8	M	White	Y			Y			Y	Y						K		N	VL	VL	Unknown	Y
7569-627.01	55	F	White			E		Y			Y			Y				60	Y	VL	VL	*TERT*	N
7569-629.01	60	M	White	Y							Y	Y		Y	MDS*		Lu	64	Y	VL	VL	*TERC*	Y
7569-629.04	33	F	White				Y				Y								Y	VL	VL	*TERC*	Y
7569-634.01	45	M	White		EG			Y			Y	Y	Y		MDS-MLD/AML			47	Y	VL	VL	*TERT (VUS) RTEL1 (VUS x2)*	Y
7569-647.01	62	M	White								Y	Y				SmCC		63	Y	VL	VL	Unknown	Y
7569-654.01	1	F	White	Y		E, LD		Y	Y	Y	Y						BM		N	n/a	n/a	*RTEL1* x2	N
7569-683.01	38	M	White								Y								Y	VL	VL	Unknown	Y
7569-687.01	12	M	White						Y		Y								N	VL	VL	*ACD* (VUS)	Y
7569-688.01	4	M	White	Y					Y		Y								Y	VL	VL	*RTEL1*	Y
7569-688.03	34	M	White																Y	VL	VL	*RTEL1*	N
7569-689.01	14	F	White	Y							Y	Y					BM		Y	VL	VL	*TERC*	Y
7569-690.01	10	F	White	Y															Y	VL	VL	*TERC*	N
7569-691.01	44	M	Unknown		EG		Y				Y			Y					Y	VL	VL	*TERT*	N
7569-695.01	51	M	White		EG						Y	Y		Y			Lu		Y	VL	NSQ	*TERC* (VUS)	N
7569-703.01	67	M	Asian					Y			Y			Y	MDS-SF3B1				Y	VL	VL	*TERT* (VUS)	Y

7569-708.01	65	M	Asian								Y	Y	Y	Y					N	VL	VL	*RTEL1* (VUS)	N
7569-713.01	72	M	White					Y			Y	Y		Y		CRC, oSCC		73		VL	VL	*TERT* (VUS x2)	Y
7569-716.01	60	M	White		EG						Y		Y	Y	MDS-MLD*		Lu	65	Y	VL	VL	Unknown	Y
7569-717.01	71	M	White	Y										Y				72	Y	VL	VL	*TERC* (VUS)	Y
7569-721.01	32	F	Asian	Y	EG		Y				Y	Y							N	VL	VL	*TERT* (VUS) *RTEL1* (VUS)	N
7569-723.01	65	M	White											Y			Lu		Y	VL	VL	*TERT* (VUS)	Y
7569-737.01	37	M	White			U													Y	VL	VL	*TERT*	N
7569-741.01	39	M	White								Y	Y			MDS-MLD				Y	VL	VL	*TERT* (VUS)	Y
7569-742.01	61	M	White								Y			Y			Lu		Y	L	L	Unknown	N
7569-755.01	29	M	White	EG															Y	VL	VL	TERT (VUS)	N
7569-757.01	52	M	White								Y			Y				52		VL	VL	TERT (VUS)	N
7569-759.01	69	M	White					Y			Y			Y			Lu		N	L	VL	Unknown	N
BCH-1	16	M	White								Y								Y	VL	n/a	*TERC*	Y
PENN_OSUMC01	37	M	White	Y	Y						Y	Y	Y	Y			Lu		Y	VL	VL	*TERC* (VUS) *ZCCHC8* *(VUS)*	Y
Penn-DC01	56	M	White			U		Y			Y	Y	Y					60	N	L	L	*TERC*	Y
Penn-DC02	28	M	White	Y							Y	Y			MDS/AML	A		30	N	VL	VL	*DKC1*	Y
Penn-DC03	70	M	White								Y			Y			Lu	71	N	VL	VL	*ACD* (VUS)	Y
Penn-DC04	55	M	White								Y	Y	Y	Y	MDS/AML			57	N	VL	VL	*RTEL1* (VUS)	Y
Penn-DC05	52	M	White	Y							Y	Y		Y	MDS-MLD	CRC, cSCC, cBCC		59	Y	n/a	n/a	*DKC1*, *RTEL1*	Y
Penn-DC06	33	M	Black	Y	EG				Y		Y	Y		Y				33	Y	VL	VL	*ZCCHC8*	Y
Penn-DC08	60	M	White		EG						Y			Y					Y	VL	VL	Unknown	N
Penn-DC09	62	M	Black								Y			Y		PTLD-DLBCL*	Lu		N	VL	VL	Unknown	N
Penn-DC10	36	M	Asian																Y	L	L	*RTEL1* (VUS)	N
Penn-DC11	22	M	White							Y	Y								Y	VL	VL	*SON* *TERT* (VUS)	N

Penn-DC12	55	M	White		EG			Y			Y				MDS-MLD				Y	L	VL	Unknown	Y
Penn-DC14	58	F	White	Y	EG						Y		Y	Y					Y	VL	VL	*TERT*	Y
Penn-DC15	55	F	White		EG			Y						Y		cBCC			Y	VL	VL	*PARN*, *TERT* (VUS)	Y
Penn-DC16	65	M	White		EG						Y			Y				65	N	L	VL	*TERT* (VUS)	Y
Penn-DC17	31	F	White								Y	Y							N	L	VL	Unknown	Y
Penn-DC18	77	F	White								Y			Y		cSCC	Lu		N	VL	VL	*RTEL1*	N
Penn-DC19	67	F	White								Y		Y						Y	L	VL	*RTEL1*	Y
Penn-DC20	72	F	White			E		Y						Y			Lu		Y	L	L	*TERT*	Y
Penn-DC21	68	M	White		EG						Y			Y		IgG MGUS*	Lu		N	L	L	*RTEL1* (VUS)	Y
Penn-DC22	62	F	White											Y					Y	L	VL	*TERC*	Y
TBD_DF_021	54	M	Asian								Y			Y	MDS-MLD		BM	56	Y	VL	VL	TERC	Y
TBD_DF_025	63	M	White		Y										MDS-MLD				N	L	VL	TERC	Y
TBD_DF_028	51	M	White	Y	EG		Y	Y			Y	Y		Y		CRC		53	Y	VL	VL	TERC	Y
TBD_DF_032	58	F	White		Y						Y				MDS-biTP53		BM		N	L	NA	TERC	Y
TBD_DF_091	58	F	White	Y	Y														Y	VL	VL	TERC	Y
TBD_DF_092	27	M	Asian	Y	T						Y	Y							Y	VL	VL	TERC	Y
TBD_DF_094	35	F	White																Y	L	VL	TERC	N
TBD_DF_117	57	F	White		Y						Y	Y	Y	Y						VL	VL	TERC	N
TBD_DF_128	31	F	White		Y					Y	Y								Y	VL	VL	TERC	N
TBD_DF_132	48	F	White								Y				MDS-IB1		BM			VL	VL	TERC	Y
TCH01	12	F	Hispanic	Y			Y		Y		Y								Y	L	VL	*TERT* *RTEL1* *WRAP53*	N
TCH02	10	M	Hispanic	Y															Y	VL	VL	*TERT*	N
TCH03	4	M	White						Y		Y	Y							Y	VL	VL	*DKC1*	N
TCH04	5	F	Hispanic						Y		Y	Y					BM		N	VL	VL	Unknown	N
TCH05	8	M	Hispanic									Y	Y				BM		N	VL	VL	*DKC1*	N
TCH06	16	M	Asian	Y	EG				Y		Y	Y							N	VL	VL	*DKC1* *WRAP53* (VUS)	N
TCH07	9	M	Hispanic	Y							Y	Y							Y	VL	VL	*TERT* *RTEL1* (VUS)	N
TCH08	14	M	Black	Y							Y	Y					K		N	VL	VL	Unknown	Y
TCH09	7	M	Hispanic	Y	EG						Y	Y							Y	VL	VL	*PARN*	Y
TCH10	5	M	Black	Y					Y		Y	Y							N	VL	VL	*TINF2*	N

TCH11	4	M	White								Y	Y							N	VL	VL	*TINF2*	N
TCH12	10	M	Black	Y		Y					Y	Y				GC/ARC*	BM	Y	N	VL	VL	*DKC1*	N
TCH13	4	M	Hispanic	Y							Y	Y		Y			BM	Y	N	VL	VL	*TINF2*	N
TCH14	2	F	Asian						Y			Y						Y	N	VL	VL	*ACD* x2	N
TCH15	1	M	Hispanic	Y							Y	Y					BM	Y	N	VL	VL	*TINF2*	N
TCH16	2	M	Asian	Y							Y	Y	Y			HA*	BM		Y	VL	VL	*TERT*	N
TCH17	9	M	Hispanic	Y			Y				Y	Y					BM, K		N	VL	VL	*DKC1*	N
TCH18	17	F	White																Y	VL	VL	*TERT*	N
TCH19	10	M	White								Y	Y					BM		N	VL	NSQ	Unknown	N
TCH20	4	M	Hispanic		EG						Y	Y					BM		N	VL	NSQ	RTEL1	N
UK 1241	13	M	Unknown	Y							Y	Y			Y				Y	VL	n/a	*TERC*	Y
UK 1455	3	M	White								Y	Y					BM			VL	n/a	*TERC*	N
UK 1580	54	M	White																Y	N	n/a	*TERC*	N
UK 1594	24	F	White	Y							Y	Y							Y	VL	n/a	*TERC*	Y
UK 2113	3	F	Black	Y	T							Y							N	N	n/a	*TERC*	N
UK 2867	52	M	White																Y	n/a	n/a	*TERC*	Y
UK 3475	53	F	White							Y	Y	Y							Y	VL	n/a	*TERC*	N
UK 3692	47	M	White	Y	T						Y	Y	Y	Y				47	Y	VL	n/a	*TERC*	Y
UK 440	10	M	White	Y							Y	Y	Y	Y					Y	VL	n/a	*TERC*	Y
UK 4804	44	M	Asian	Y							Y		Y	Y	Y		BM	47		VL	n/a	*TERC*	Y
UK 520	52	F	White	Y							Y	Y		Y					Y	VL	n/a	*TERC*	Y
UK 641	7	M	White																Y	VL	n/a	*TERC*	N
UK 813	27	M	White								Y	Y							Y	VL	n/a	*TERC*	Y
UK 850	42	F	White	Y	EG		Y	Y			Y	Y						Y	Y	VL	n/a	*TERC*	Y
UW001	42	M	White	Y	EG						Y	Y							Y	L	VL	*TERC*	Y
UW002	27	M	White	Y		U					Y	Y				oSCC*	BM		Y	VL	n/a	*RTEL1* (VUS) *TERC* (VUS)	N
UW003	29	M	White	Y							Y								Y	n/a	n/a	*TINF2*	Y
UW004	42	M	White	Y	EG						Y	Y	Y				Li		N	VL	VL	*PARN* (VUS)	N
UW005	57	M	White	Y				Y			Y	Y		Y	MDS-RAEB-2	cSCC*	BM		Y	n/a	n/a	*TERC*	N
UW007	35	F	White	Y	EG										APL	M			Y	L	VL	*PARN*	Y
UW008	66	M	White			U								Y			Lu		Y	L	VL	*PARN*	N

UW009	63	M	Black		EG			Y			Y			Y				63	N	L	L	*TERT*	N
UW010	69	F	White					Y			Y	Y	Y	Y	MDS-SF3B1	aSCC*	Li	69	Y	VL	VL	Unknown	Y
UW011	58	M	White	Y				Y			Y			Y			Lu		Y	VL	VL	*RTEL1*	N
UW012	45	M	White	Y	EG				Y		Y	Y		Y	CML		BM, Lu	66	Y	n/a	n/a	*RTEL1* x2 (1x VUS)	Y
UW013	55	F	White		EG									Y			Lu		Y	L	VL	*RTEL1* (VUS x 2)	N
UW014	65	M	White	Y				Y			Y	Y		Y					Y	L	VL	*RTEL1* (VUS) *ACD* (VUS)	N
UW015	60	F	White	Y				Y			Y			Y			Lu		Y	VL	VL	*RTEL1* (VUS)	N
UW016	52	M	White	Y	EG			Y			Y		Y	Y			Li		Y	VL	VL	*TERT* (VUS)	N
UW017	33	M	White	Y							Y		Y						Y	VL	VL	*TERT* (VUS)	N
UW018	69	M	White											Y					Y	L	L	*RTEL1*	N
UW019	51	M	White	Y							Y			Y				52	Y	VL	VL	*RTEL1*	Y
UW020	21	M	White	Y	EG														Y	VL	VL	*TERT*	N
UW021	70	M	White											Y				72	Y	L	L	*RTEL1*	N
UW022	28	M	White	Y				Y											Y	VL	VL	*RTEL1*	Y
UW023	31	F	White	Y	EG														Y	L	L	*TERT*	N
UW024	71	F	White	Y				Y			Y			Y					Y	VL	VL	*TERT* *ACD* (VUS)	N
UW025	63	M	White	Y	EG						Y			Y					Y	L	VL	*TERT* (VUS)	N
UW026	68	F	White	Y	EG						Y			Y		pSCC		69	Y	L	VL	Unknown	N
UW027	61	M	White	Y							Y			Y		oSCC	Lu, H		Y	L	VL	Unknown	N
UW028	43	M	White	Y															Y	VL	VL	Unknown	N
UW029	66	F	White											Y					Y	VL	VL	Unknown	N
UW030	78	F	White	Y							Y			Y		cervSCC			Y	VL	VL	Unknown	Y
UW032	50	F	White	Y										Y					Y	VL	L	*PARN* (VUS)	N
UW033	53	M	White	Y	Y			Y			Y			Y			Lu		Y	VL	VL	*TERT* (VUS)	Y
UW034	65	M	White	Y	Y						Y			Y					Y	VL	VL	RTEL1	Y
WU006-52	59	M	White							Y	Y			Y			Y			n/a	n/a	*TERC* (VUS)	Y
WU008-40	65	M	White											Y			Y	Y		n/a	n/a	*TERT*	Y
WU008-57	56	M	White								Y			Y			Y			n/a	n/a	*RTEL1*	Y

WU009-04	65	F	White								Y			Y			Y	Y		n/a	n/a	*TERT*	N
WU010-20	59	M	White							Y	Y			Y			Y			n/a	n/a	*PARN*	N
WU010-25	68	M	White								Y			Y			Y			n/a	n/a	*TERT*	Y
WU013-59	61	F	White								Y			Y			Y			n/a	n/a	*PARN*	Y
WU013-65	48	F	White							Y	Y			Y			Y			n/a	n/a	*RTEL1*<br>*TERT*	Y
WU015-03	60	M	White								Y			Y			Y			n/a	n/a	*RTEL1*	N
WU015-15	64	M	White								Y			Y			Y			n/a	n/a	*RTEL1*	N
WU015-34	75	M	White								Y			Y			Y	Y		n/a	n/a	*RTEL1*	N
WUSTL420.1	1	F	Black																	L	n/a	*TERT* x2<br>(1x VUS)	Y

Age reported is at the time of first specimen collection or first specimen collection with clonal hematopoiesis. Deceased indicates age at death. Telomere length reported is based on lymphocyte subset. Evidence of clonal hematopoiesis refers to skewed X-inactivation, clonal karyotype abnormalities, or clonal gene variants identified on blood or bone marrow specimens. (T = thinning hair, EG = early graying, WF/GF = white/gray forelock, E = esophageal web or stricture, U = urethral stricture, La = lacrimal duct stenosis, PD = parotid duct stenosis, MDS = myelodysplastic syndrome, MLS = multilineage dysplasia, RAEB = refractory anemia with excess blasts, APL = acute promyelocytic leukemia, AML = acute myeloid leukemia, oSCC = oral cavity squamous cell carcinoma, cSCC = cutaneous squamous cell carcinoma, cBCC = cutaneous basal cell carcinoma, *post-transplant, PTLD = post-transplant lymphoproliferative disorder, SmCC = small cell carcinoma, CRC = colorectal adenocarcinoma, HA = hepatic angiosarcoma, A = astrocytoma, DLBCL = diffuse large B cell lymphoma, GC/ARC = synchronous gastric and anorectal adenocarcinoma, MGUS = monoclonal gammopathy of unknown significance, PC = pulmonary carcinoid, M = melanoma, aSCC = anal squamous cell carcinoma, pSCC = pulmonary squamous cell carcinoma, cervSCC = cervical squamous cell carcinoma, Lu = lung transplant, Li = liver transplant, K = kidney transplant, BM = hematopoietic stem cell transplant, H = heart transplant, VL = very low (<1%ile), L = low (1-10%ile).

Supplemental Table S2: Summary of clinical findings used to establish a TBD diagnosis in subjects without a genetic confirmation of TBD or subjects for whom results of telomere length testing were unavailable.

Patient ID	Findings
7569-009.01	Telomere lengths <1st %ile in lymphocytes<br>Manifestations include bone marrow failure, oral leukoplakia, esophageal webbing/stricture, osteoporosis<br>Family history of aplastic anemia<br>Compound heterozygous *TERT* variants of uncertain significance identified on germline testing
7569-012.01	Telomere lengths <1st %ile in all of 6 tested subsets<br>Manifestations include bone marrow failure, osteopenia<br>Mother with short telomeres (7569-012.02); other ancestors with short telomeres, osteopenia, cytopenias, bone marrow failure, solid tumors<br>No germline variants identified
7569-012.02	Telomere lengths <1st%ile in lymphocytes<br>No current manifestations<br>Son (7569-012.01) with short telomeres, osteopenia, bone marrow failure; other ancestors with short telomeres, osteopenia, cytopenias, bone marrow failure, solid tumors<br>No germline variants identified
7569-328.01	Telomere lengths <1st %ile in lymphocytes, 1-10th %ile in granulocytes<br>Manifestations include osteopenia, short stature (delayed growth on growth hormone), adrenal insufficiency, developmental and speech delay, B-cell immunodeficiency with hypogammaglobulinemia, pulmonary adenovirus s/p bilateral lung transplant complicated by bronchiolitis obliterans with organizing pneumonia, chronic kidney disease, pancytopenia, nail dystrophy<br>Family history of premature graying<br>Hemizygous *DKC1* variant of uncertain significance identified on germline testing
7569-522.01	Telomere lengths <1st %ile in lymphocytes and granulocytes<br>Manifestations include cirrhosis liver transplant, aplastic anemia, osteoporosis, osteonecrosis of left knee, urethral stricture, nail dystrophy, early graying<br>Family history notable for cirrhosis and early graying<br>Heterozygous *TERT* variant of uncertain significance identified on germline testing
7569-534.01	Telomere lengths <1st %ile in lymphocytes, 1-10th %ile in granulocytes<br>Manifestations include mucocutaneous features, squamous cell carcinoma of buccal mucosa in early adulthood<br>No relevant family history<br>No germline variants identified
7569-587.01	Telomere lengths <1st %ile in naïve T cells and memory T cells; 1-10th %ile in lymphocytes, B cells, and NK cells (granulocytes NSQ)<br>Manifestations include poor dentition, thin hair and nails, developmental delay, mild ataxia, microcephaly<br>Family history notable for shared *TERT* variant with short telomeres, premature graying, prostate cancer, breast cancer<br>Heterozygous *TERT* variant of uncertain significance identified on germline testing
7569-596.01	Telomere lengths <1st %ile in lymphocytes and granulocytes<br>Manifestations include reticular skin pigmentation, nail abnormalities, cytopenias, developmental delay and short stature, and avascular necrosis of the hip<br>No relevant family history<br>No germline variants identified
7569-634.01	Telomere lengths <1st %ile in lymphocytes and granulocytes<br>Disease manifestations include pancytopenia, MDS-MLD with transformation to to AML, branch retinal vein occlusion and cystoid macular edema, liver cirrhosis with ascites and esophageal varices, osteopenia, early greying<br>Family history notable for short telomeres with shared germline variants and Hodgkin lymphoma<br>Two heterozygous *RTEL1* variants of uncertain significance as well as one heterozygous *TERT* variant of uncertain significance identified on germline testing
7569-647.01	Telomere lengths <1st %ile in lymphocytes and granulocytes<br>Manifestations include bone marrow failure and small cell carcinoma of the lung<br>Son (7569-683.01) also has short telomeres and cytopenias; no ancestors with TBD<br>No germline variants identified

7569-683.01	Telomere lengths <1st %ile in lymphocytes and granulocytes<br>Manifestations include dental abnormalities and bone marrow failure<br>Family history notable for short telomeres, solid tumor, and bone marrow failure<br>No germline variants identified
7569-687.01	Telomere lengths <1st %ile in lymphocytes and granulocytes<br>Manifestations include cytopenias, developmental delay, and immune deficiency (T/NK lymphopania and impaired T/B cell function)<br>No relevant family history<br>Heterozygous *ACD* variant of uncertain significance identified on germline testing
	Telomere lengths <1st %ile in lymphocytes, granulocytes, naïve T cells, memory T cells, and B cells; 1-10th %ile NK cells<br>Manifestations include interstitial lung disease s/p *bilateral* lung transplant, early graying, post-transplant neutropenia (resolved) and T-LGL lymphocytosis<br>Family history notable for early graying, breast cancer, and colon cancer<br>Heterozygous *TERC* variant of uncertain significance identified on germline testing
7569-703.01	Telomere lengths <1st %ile in lymphocytes and granulocytes<br>Manifestations include early graying, osteoporosis, bone marrow failure, MDS, interstitial lung disease<br>Family history notable for interstitial lung disease<br>Heterozygous *TERT* variant of uncertain significance identified on germline testing
7569-708.01	Telomere lengths <1st %ile in lymphocytes and granulocytes<br>Manifestations include bone marrow failure, interstitial lung disease, and cirrhosis<br>Family history notable for liver disease and son (Penn-DC10) with shared *RTEL1* mutation and telomeres <10th %ile<br>Heterozygous *RTEL1* variant of uncertain significance identified on germline testing
7569-713.01	Telomere lengths <1st %ile in lymphocytes and granulocytes<br>Manifestations include bone marrow failure, cytopenias with lifelong macrocytic anemia, interstitial lung disease, squamous cell carcinoma of the tongue, rectal adenocarcinoma, and thoracic osteopenia<br>Family history unknown (adopted)<br>Two heterozygous *TERT* variants of uncertain significance identified on germline testing
7569-716.01	Telomere lengths <1st %ile in lymphocytes and granulocytes<br>Manifestations include early graying, bone marrow failure, MDS, interstitial lung disease, and cirrhosis<br>Family history notable for pulmonary fibrosis, cirrhosis, and solid tumors<br>No germline variants identified
7569-717.01	Telomere lengths <1st %ile in lymphocytes, granulocytes, naïve T cells, and B cells; 1-10th %ile memory T cells and NK cells<br>Manifestations include interstitial lung disease, nail dystrophy<br>Family history *notable* for interstitial lung disease, prostate cancer, and Paget disease<br>Heterozygous *TERC* variant of uncertain significance identified on germline testing
7569-721.01	Telomere lengths <1st %ile in lymphocytes and granulocytes<br>Manifestations include plastic anemia, early graying, skin hypopigmentation, avascular necrosis of the hip<br>Family history notable for early graying, cirrhosis (alcohol-related)<br>Heterozygous *TERT* variant of uncertain significance and heterozygous *RTEL1* variant of uncertain significance identified on germline testing
7569-723.01	Telomere lengths <1st %ile in lymphocytes and granulocytes<br>Manifestations include pulmonary fibrosis s/p lung transplant<br>Family history notable for father and brother with interstitial lung disease, brother with short telomeres and died of lymphoma at age 24<br>Heterozygous *TERT* variant of uncertain significance identified on germline testing
7569-741.01	Telomere lengths <1st %ile in lymphocytes and granulocytes<br>Manifestations include bone marrow failure and MDS<br>Family history notable for pulmonary fibrosis, oral and liver cancers<br>Heterozygous *TERT* variant of uncertain significance identified on germline testing
7569-742.01	Telomere lengths 1-10%ile in lymphocytes and granulocytes<br>Manifestations include cytopenias and interstitial lung disease<br>Family history notable for interstitial lung disease in multiple generations<br>No germline variants identified

7569-755.01	Telomere lengths <1st %ile in lymphocytes and granulocytes<br>Manifestations include early graying<br>Family history notable for early graying, father with interstitial lung disease and shared *TERT* variant, paternal uncle with interstitial lung disease and leukemia<br>Heterozygous *TERT* variant of uncertain significance identified on germline testing
7569-757.01	Telomere lengths <1st %ile in lymphocytes, granulocytes, naïve T cells, memory T cells, and B cells; 1-10th %ile NK cells<br>Manifestations include pulmonay fibrosis, cytopenias<br>No relevant family history<br>Heterozygous *TERT* variant of uncertain significance identified on germline testing
7569-759.01	Telomere lengths <1st %ile in granulocytes and <10th %ile in lymphocytes<br>Manifestations include interstitial lung disease s/p transplant, cytopenias, osteoporosis<br>No relevant family history<br>No germline variants identified
PENN_OSUMC01	Telomere lengths <1st %ile in lymphocytes and granulocytes<br>Manifestations include mucocutaneous features, early graying/white forelock, lymphopenia, cytopenias, bone marrow failure, cirrhosis, lung disease<br>Family history of related disease<br>Heterozygous *TERT* variant of uncertain significance and heterozygous *ZCCHC8* variant of uncertain significance identified on germline testing
Penn-DC03	Telomere lengths <1st %ile in lymphocytes, granulocytes, naïve T cells, memory T cells, and B cells; 1-10th %ile NK cells<br>Manifestations include interstitial lung disease s/p transplant, pancytopenia<br>Family history notable for lung cancer<br>Heterozygous *ACD* variant of uncertain significance identified on germline testing
Penn-DC04	Telomere lengths at 1st %ile in lymphocytes and <1st %ile in granulocytes<br>Manifestations include bone marrow failure, MDS/AML, cirrhosis, interstitial lung disease<br>Family history norable for breast cancer<br>Heterozygous *RTEL1* variant of uncertain significance identified on germline testing
Penn-DC08	Telomere lengths <1st %ile in lymphocytes and granulocytes<br>Manifestations include early graying, cytopenias, and pulmonary fibrosis<br>Family history notable for pulmonary fibrosis<br>No germline variants identified
Penn-DC09	Telomere lengths <1st %ile in lymphocytes and granulocytes<br>Manifestations include cytopenias and pulmonary fibrosis<br>No relevant family history<br>No germline variants identified
Penn-DC10	Telomere lengths 1-10 %ile in lymphocytes and granulocytes<br>No current manifestations<br>Family history notable for father (708.01) with short telomeres, shared RTEL1 mutation, and severe phenotype<br>Heterozygous *RTEL1* variant of uncertain significance identified on germline testing
Penn-DC12	Telomere lengths <1st %ile in granulocytes and <10th %ile in lymphocytes<br>Manifestations include early graying, osteoporosis, cytopenias, and MDS<br>Family history notable for early graying, osteoporosis, solid tumors, and leukemia<br>No germline variants identified
Penn-DC15	Telomere lengths <1st %ile in lymphocytes, granulocytes, naïve T cells, memory T cells, and B cells; 1-10th %ile NK cells<br>Manifestations include early graying, avascular necrosis, interstitial lung disease, basal cell carcinoma of skin<br>Family history notable for early graying, avascular necrosis, interstitial lung disease<br>Heterozygous *TERT* variant of uncertain significance and pathogenic *PARN* variant identified on germline testing
Penn-DC16	Telomere lengths <1st %ile in granulocytes and <10th %ile in lymphocytes<br>Manifestations include early graying, cytopenias, hepatic fibrosis, and interstitial lung disease<br>Family history notable for early graying<br>Heterozygous *TERT* variant of uncertain significance identified on germline testing
Penn-DC17	Telomere lengths <1st %ile in granulocytes and <10th %ile in lymphocytes<br>Manifestations include dental abnormalities and bone marrow failure<br>No relevant family history<br>No germline variants identified

Penn-DC21	Telomere lengths 1st-10th %ile in lymphocytes and granulocytes<br>Manifestations include early graying, macrocytic anemia, and interstitial lung disease<br>No relevant family history<br>Heterozygous *RTEL1* variant of uncertain significance identified on germline testing
TCH04	Telomere lengths <1st %ile in lymphocytes and granulocytes<br>Manifestations include immunodeficiency and bone marrow failure<br>No relevant family history<br>No germline variants identified
TCH08	Telomere lengths <1st %ile in lymphocytes and granulocytes<br>Manifestations include mucocutaneous features and bone marrow failure<br>No relevant family history<br>No germline variants identified
TCH19	Telomere lengths <1st %ile in lymphocytes and granulocytes<br>Manifestations include mucocutaneous features and bone marrow failure<br>No relevant family history<br>No germline variants identified
UW002	Telomere lengths <1st %ile in lymphocytes<br>Manifestations include mucocutaneous features, urethral stricture, solid tumor, and bone marrow failure<br>Family history of related disease<br>Heterozygous *RTEL1* variant of uncertain significance and heterozygous *TERC* variant of uncertain significance identified on germline testing
UW004	Telomere lengths <1st %ile in lymphocytes and lymphocytes<br>Manifestations include mucocutaneous features, early graying, bone marrow failure, and cirrhosis<br>No relevant family history<br>Heterozygous *PARN* variant of uncertain significance identified on germline testing
UW010	Telomere lengths <1st %ile in lymphocytes and lymphocytes<br>Manifestations include osteropenia/porosis, bone marrow failure, myeloid neoplasm, cirrhosis, interstitial lung disease, and solid tumor<br>Family history of related disease<br>No germline variants identified
UW012	Telomere lengths not available (post bone marrow transplant<br>Manifestations include mucocutaneous features, early graying, immunodeficiency, bone marrow failure, interstitial lung disease s/p lung transplant, CML s/p bone marrow transplant<br>Family history of related disease<br>Heterozygous, pathogenic *RTEL1* variant as well as a heterozygous *RTEL1* variant of uncertain significance identified on germline testing
UW013	Telomere lengths 1-10th %ile in lymphocytes, <1st %ile in granulocytes<br>Manifestations include early graying, cytopenias, interstitial lung disease<br>Family history of related disease<br>Two heterozygous *RTEL1* variants of uncertain significance identified on germline testing
UW014	Telomere lengths 1-10th %ile in lymphocytes, <1st %ile in granulocytes<br>Manifestations include mucocutaneous features, osteopenia/porosis, bone marrow failure, and interstitial lung disease<br>*Family* history of related disease<br>Heterozygous *RTEL1* variant of uncertain significance as well as a heterozygous *ACD* variant of uncertain significance identified on germline testing
UW015	Telomere lengths <1st %ile in lymphocytes and granulocytes<br>Manifestations include mucocutaneous features, osteopenia/porosis, cytopenias, interstitial lung disease<br>Family history of related disease<br>Heterozygous *RTEL1* variant of uncertain significance identified on germline testing
UW016	Telomere lengths <1st %ile in lymphocytes and granulocytes<br>Manifestations include mucocutaneous features, early graying, osteopenia/porosis, cytopenias, cirrhosis, and interstitial lung disease<br>Family history of related disease<br>Heterozygous *TERT* variant of uncertain significance identified on germline testing

UW017	Telomere lengths <1st %ile in lymphocytes and granulocytes<br>Manifestations include mucocutaneous features, cytopenias, and cirrhosis<br>Family history of related disease<br>Heterozygous *TERT* variant of uncertain significance identified on germline testing
UW025	Telomere lengths 1-10th %ile in lymphocytes, <1st %ile in granulocytes<br>Manifestations includes mucocutaneous features, early graying, cytopenias, interstitial lung disease<br>Family history of related disease<br>Heterozygous *TERT* variant of uncertain significance identified on germline testing
UW026	Telomere lengths 1-10th %ile in lymphocytes, <1st %ile in granulocytes<br>Manifestations include mucocutaneous features, early graying, cytopenias, solid tumor, and interstitial lung disease<br>Family history of related disease<br>No germline variants identified
UW027	Telomere lengths 1-10th %ile in lymphocytes, <1st %ile in granulocytes<br>Manifestations include mucocutaneous features, early graying, cytopenias, solid tumor, and interstitial lung disease<br>Family history of related disease<br>No germline variants identified
UW028	Telomere lengths <1st %ile in lymphocytes and granulocytes<br>Manifestations include mucocutaneous features<br>Family history of related disease (related to UW029)<br>No germline variants identified
UW029	Telomere lengths <1st %ile in lymphocytes and granulocytes<br>Manifestations include interstitial lung disease<br>Family history of related disease (related to UW028)<br>No germline variants identified
UW030	Telomere lengths <1st %ile in lymphocytes and granulocytes<br>Manifestations include mucocutaneous features, cytopenias, and solid tumor, and interstitial lung disease<br>Family history of related disease<br>No germline variants identified
UW032	Telomere lengths <1st %ile in lymphocytes, 1-10th %ile in granulocytes<br>Manifestations include mucocutaneous features and interstitial lung disease<br>Family history of related disease<br>Heterozygous *PARN* variant of uncertain significance identified on germline testing
UW033	Telomere lengths <1st %ile in lymphocytes and granulocytes<br>Manifestations include mucocutaneous features, early graying, osteopenia/porosis, cytopenias, and interstitial lung disease s/p transplant<br>Family history or related disease<br>Heterozygous *TERT* variant of uncertain significance identified on germline testing
WU006-52	Telomere lengths not available<br>Manifestations include endocrine dysfunction, cytopenias, and interstitial lung disease s/p transplant<br>Heterozygous *TERT* variant of uncertain significance identified on germline testing

Supplemental Table S3: Clonal hematopoiesis findings

Patient ID	Age (yrs)	Clonality Studies	Germline Variants in TBD-Related Genes	Acquired Somatic Alterations (variant allele fraction)	Miscellaneous Presumed or Confirmed Germline Variants
7569-009.01	16	Karyo, SNP-A, WES, NGS-TBD	*TERT* c.1610G>A p.R537H (VUS) het<br>*TERT* c.2173_2187del15insACAG p.L725_I729delfs*39 (VUS) het	Age 16: *LPHN1* c.1189G>A p.G397R (16%)	None identified
7569-012.01	13	Karyo, NGS-TBD	None identified	None identified at age 13	None identified
7569-012.02	40	HA, NGS-TBD	None identified	Age 40: Skewed X-chromosome inactivation (>74%)	None identified
7569-023.01 (WUSTL 320.01)	53	WES, NGS-TBD	*DKC1* c.91C>G p.Q31E hem	Age 53: *TTN* NM_001267550.2:c.10030C>T p.P3344S (33%)	None identified
7569-047.01	12	Karyo, NGS-TBD	*RTEL1* NM_032957:c.1845G>T p.E615D het<br>*RTEL1* NM_032957:c.2992C>T p.R998X het	None identified at age 12	None identified
7569-047.04	5	NGS-TBD	*RTEL1* NM_032957:c.G1845T p.E615D *het*	None identified at age 5	*ATM* c.8419-1delGTGA p.? (43%)
7569-060.01	20	Karyo, SNP-A, NGS-TBD	*TERC* NR_001566.2:n.96_97delCT het	Age 20: 46,XY,-17,+mar[1];46,XY[19]<br>Age 22: No sequencing variants identified	None identified
7569-060.02	54	Karyo, SNP-A, HA, WES, NGS-TBD	*TERC* NR_001566.2:n.96_97delCT het	Age 54:<br>Skewed X-chromosome inactivation (>74%)<br>46,XX,der(16)t(1;16)(q21;q24)[14]/46,XX[6]	None identified
7569-103.03	20	HA, NGS-TBD	*TERC* NR_001566.2:n.35C>T het	None identified at age 20	None identified
7569-103.04	32	HA, NGS-TBD	*TERC* NR_001566.2:n.35C>T het	Age 32:<br>Skewed X-chromosome inactivation (>74%)<br>*AHNAK2* NM_001350929:c.G6751A p.D2251N (17.8%)<br>*GATA2* NM_001145661:c.C481G p.P161A (17.5%)<br>*ITSN2* NM_001348181:c.2215+1G>A p.? (15.3%)<br>*NEK4* NM_001193533:c.T1432C p.F478L (17.1%)<br>*PALB2* NM_024675:c.G2014C p.E672Q (15.0%)<br>*TELO2* NM_01611:c.C1726T p.R576C (17.2%)	None identified
7569-103.05	17	HA, NGS-TBD	*TERC* NR_001566.2:n.35C>T het	None identified at age 17	None identified
7569-103.09	29	HA	*TERC* NR_001566.2:n.35C>T het	Age 29: Skewed X-chromosome inactivation (>74%)	None identified
7569-103.10	25	NGS-TBD	*TERC* NR_001566.2:n.35C>T het	None identified at age 25	None identified
7569-103.15	33	NGS-TBD	*TERC* NR_001566.2:n.35C>T het	None identified at age 33	None identified
7569-109.01	31	Karyo	*TERT* p.K902N het	None identified at age 31	46,XY,inv(2)(p11q13)[20]
7569-134.03 (WUSTL 141.03)	24	WES	*DKC1* NM_001363.5:c.1058C>T p.A353V hem	None identified at age 24	None identified
7569-146.01	11	HA	*TERT* NM_198253.3:c.2593C>G p.R865G het<br>*TERT* NM_198253.3:c.2683C>G p.L895F het	Age 11: Skewed X-chromosome inactivation (>74%)	None identified
7569-199.01	16	Karyo, NGS-TBD	*DKC1* NM_001363.2:c.1058C>T p.A353V hem	None identified at age 16	None identified
7569-257.01	17	Karyo, SNP-A, NGS-TBD	*DKC1* NM_001363.5:c.29C>T p.P10L hem	None identified at age 17	None identified

7569-328.01	11	Karyo, SNP-A, WES, NGS-TBD	*DKC1* NM_001363.5:c.838A>C p.S280R hem (VUS)	None identified at age 11	None identified
7569-373.01	14	Karyo, SNP-A, WES, NGS-TBD	*TERC* n.173A>G het	None identified at age 14	None identified
7569-373.02	41	Karyo, SNP-A, HA, NGS-TBD	*TERC* n.173A>G het	Age 41: Skewed X-chromosome inactivation (>74%)	None identified
7569-373.04	10	Karyo, SNP-A, HA, NGS-TBD	*TERC* n.173A>G het	Age 10: Skewed X-chromosome inactivation (>74%)	None identified
7569-398.01	21	Karyo, SNP-A, WES, NGS-HMP, NGS-PS, NGS-TBD	*DKC1* c.-35G>A hem	Age 21: *DKC1* c.-35G>A (14%)<br>Age 22: Normal karyotype<br>Age 27: Normal karyotype, no somatic variants<br>Age 28: Normal karyotype, no somatic variants<br>Age 30: *U2AF1* c.101C>T p.S34F NM_006758.2 (VAF 2%)<br>Age 31: *U2AF1* c.101C>T p.S34F NM_006758.2 (VAF 2%)<br>Age 33: Normal karyotype, no somatic variants	*HFE* NM_000410.3:c.187C>G p.H63D het<br>*SF3A1* NM_005877:c.1083T>A p.D361E (48% VUS)<br>cnLOH over *CTC1* and *TERC*
7569-522.01	35	Karyotype, SNP-A, NGS-TBD	*TERT* NM_198253:c.3115A>G p.T1039A het (VUS)	Age 35: *TERT* NM_198253.2:c.-124C>T p.? (13%)	*HFE* NM_000410.3:c.187C>G p.H63D het
7569-529.01	16	Karyo, SNP-A, NGS-TBD	*RTEL1* NM_032957.4:c.3578C>A p.S1193* het	None identified at age 16	3q21.1 CNL (*DIRC2*)<br>del12q23.1 CNL (*HAL*, *LTA4H*)
7569-534.01	17	Karyo, SNP-A, NGS-TBD	None identified	None identified at age 17	14q32.33 CNL<br>19q13.42 CNL (KIR2DL4)
7569-587.01	3	Karyo	*TERT* NM_198253.2:c.2852G>A p.R951Q het (VUS)	None identified at age 3	*KMT2B* NM_014727.2:c.3057dupA p.G1020Rfs<br>1p22.2 dup
7569-596.01	8	Karyo, SNP-A, NGS-TBD	None identified	Age 8: *KMT2C* NM_170606.2 p.R973* (6% VUS)	17p12dup<br>*ATP2B3* NM_001001344.2:c.2414C>T p.A805V hem (VUS)<br>*TRPC6* NM_004521.5:c.1354A>G p.I452V het (VUS)
7569-627.01	55	Karyo, SNP-A, NGS-HMP, NGS-PS, NGS-TBD	*TERT* NM_198253.3:c.2240del p.V747fs het	None identified at ages 55, 59	*IL7R* NM_002185.3:c.616C>T p.R206* (51% VUS)
7569-629.01	60	Karyo, SNP-A, NGS-HMP, NGS-PS, NGS-TBD	*TERC* NR_001566.1:r.314T>A het	Age 60:<br>*ATM* NM_000051:c.2839-2G>A p.? (6%)<br>*ATM* NM_000051:c.6219_6256dup p.Y2086Sfs*9 (14%)<br>*ATM* NM_000051:c.8495G>A p.R2832H (6% VUS)<br>*ATM* NM_000051:c.8672G>A p.G2891D (21% VUS)<br><br>Age 61 years:<br>*ATM* NM_000051:c.2839-2G>A p.? (<5%)<br>*ATM* NM_000051:c.6219_6256dup p.Y2086Sfs*9 (15%)<br>*ATM* NM_000051:c.8495G>A p.R2832H (6% VUS)<br>*ATM* NM_000051:c.8672G>A p.G2891D (23% VUS)<br><br>Age 64 years (MDS):<br>*ATM* NM_000051:c.6219_6256dup p.Y2086Sfs*9 (41%)<br>*NPM1* NM_002520.6:c.860_863dup p.W288Cfs*12 (32%)<br>*ATM* NM_000051:c.8672G>A p.G2891D (44% VUS)	*ASXL1* NM_015338:c.3755A>G p.D1252G (VUS)

7569-629.04	33	Karyo, SNP-A, NGS-PS	*TERC* NR_001566.1:r.314T>A het	Age 33.3:<br>*ATM* NM_000051.3:c.7629+1G>A p.? (VAF <7%)<br>*ATM* NM_000051.3:c.7880A>G p.Y2627C (VAF <7%)<br>*PPM1D* NM_003620.3:c.1430_1431insT p.C478Lfs*3 (1.8%)<br><br>Age 35.6:<br>*ATM* NM_000051.3:c.7629+1G>A p.? (VAF 10%)<br>*ATM* NM_000051.3:c.7880A>G p.Y2627C (VAF 8%)<br>*PPM1D* NM_003620.3:c.1596_1603del p.N533Kfs*16 (VAF 0.4%)<br><br>Age 36.3<br>*ATM* NM_000051.3:c.7629+1G>A p.? (VAF 13%)<br>*ATM* NM_000051.3:c.7880A>G p.Y2627C (VAF 15%)<br>*PPM1D* NM_003620.3:c.1430_1431insT p.C478Lfs*3 (0.3%)<br>*PPM1D* NM_003620.3:c.1596_1603del p.N533Kfs*16 (0.6%)<br>*PPM1D* NM_003620.3:c.1405_1406insAAGATCC p.E472Rfs*6 (0.4%)	*ASXL1* NM_015338.5:c.3755_3759delinsGCAGC p.D1252G (VUS)<br>*FANCD2* NM_033084.4:c.28T>C p.S10P (VUS)<br>*FANCM* NM_020937.3:c.4531T>C p.F1511L (VUS)
7569-634.01	45	Karyo, FISH, NGS-Neo, NGS-S, NGS-TBD	*TERT* NM_198253.3:c.2936G>A p.R979Q het (VUS)<br>*RTEL1* NM_001283009.1:c.572T>G p.V191G het (VUS)<br>*RTEL1* NM_001283009.1:c.1261C>G p.Q421E het (VUS)	Age 45 (MDS):<br>45~49,XY,+1,add(1)(q12),-5,i(6)(p10),del(7)(p15),add(7)(q11.2),dic(7;17)(q11.2;p11.2),i(8)(q10),+9,+11,-17,-18,+14mar[cp17]/96<4n>,idemx2[2]/46,XY[1]<br>FISH: del5q31-q33 [122/200]; del7q22-q31 [150/200]; three copies of *KMT2A*<br><br>Age 46 (MDS):<br>*TP53* NM_000546.5:c.782+1G>A p.? (7%)<br>46,XY[20]<br><br>Age 47 (MDS):<br>*TP53* NM_000546.5:c.1009C>T p.R337C (60%)<br>*TP53* NM_000546.5:c.782+1G>A p.? (6%)<br>*RUNX1* NM_001754.4 p.R204L (7%)<br>*TP53* deletion [49/200]<br>41~49,XY,-5,der(7;17)(p10;q10),+8,+9,+11,+13,+1~4mar[cp8]/45,XY,add(9)(p13),-18[1]/46,XY[11]<br><br>Age 47.5 (MDS):<br>45~46,XY,-5,add(7)(q11.2),+9,+11,add(12)(p11.2),add(13)(q14),der(14;21)(q10;q10),-17,-20,der(21)del(21)(q22)add(21)(p11.2)[10]/45~46,idem,-14,+1~2mar[cp9]/46,XY[5]<br>nuc ish(*RUNX1T1*x2,*RUNX1*x1)[190/200]	None identified
7569-647.01	62	Karyo, SNP-A, NGS-HMP, NGS-TBD	None identified	Age 62: *ASXL1* NM_015338:c.1934dup p.G646Wfs*12 (10%)	3q21.3dup het (VUS - shared with son)<br>*RAD21* NM_006265.2:c.868A>G p.M290V (46% VUS)
7569-654.01	1	Karyo	*RTEL1* NM_001283009.1:c.1255C>T p.Q419* het<br>*RTEL1* NM_001283009.1:c.3730T>C p.C1244R het	None identified at age 1	None identified

7569-683.01	38	Karyo, SNP-A, NGS-HMP, NGS-TBD	None identified	Age 38:<br>46,XY,add(14)(q32)[10]/46,idem,der(21)t(1;21)(q12;p11.2)[10] nuc ish(IGHx2)(3'IGHC sep 5'IGHVx1)[190/200] ish t(1;14)(q43;q32)(5'IGHV+;5'IGHV-)[6]<br><br>Age 39:<br>46,XY,add(14)(q32)c,der(21)t(1;21)(q12;p11.2)[3]/46,XY,add(14)c[17]	3q21.3dup het (VUS - shared with father)
7569-687.01	12	Karyo, SNP-A, NGS-CHOP, NGS-TBD	*ACD* NM_001082486.1:c.1232C>T p.T411I het (VUS)	Age 13:<br>*KAT6B* NM_001370135:c.877_888del p.D296_E299del (21.6%)<br>*RTEL1* NM_001283009:c.C1940T p.P647L (26.4%)<br>*TOP1* NM_003286:c.T1787C p.I596T (26.6%)	*SETD2* NM_014159.6:c.33G>C pL11N het (VUS)<br>*KMT2A* NM_005933.3:c.5543T>G; p.I1848S het (VUS)<br>*PRPF40B* NM_001031698.2:c.2313T>G p.D771E het (VUS)<br>*PHF6* NM_032458.2:c.1070T>G; p.L357R het (VUS)
7569-688.01	4	Karyo, SNP-A, NGS-TBD	*RTEL1* NM_001283009.1:c.1940C>T p.P647L het	Age 4: *MIR4453HG* chr4 152536365 A>G (21.2%)	*ETS1* NM_005238.3:c.471T>A p.D157E (48% VUS)<br>*ETV6* NM_001987.4:c.463+2T>C p.? het (VUS)<br>*CARD11* NM_032415.5:c.1994C>T p.S665L (VUS)<br>*MEFV* NM_000243.2:c.2084A>G p.L695R het (VUS)<br>*SPOP* NM_001007226.1:c.208C>T pR70* het (VUS)
7569-688.03	34	Karyo, NGS-PS	*RTEL1* NM_001283009.1:c.1940C>T p.P647L het	None identified at age 34	*BRCA1* NM_007300.3:c.3037G>C p.E1013Q (VUS)<br>*CBL* NM_005188.3:c.1754G>T p.R585L (VUS)<br>*PLCG1* p.I1209V c.3625A>G NM_002660.2 (VUS)
7569-689.01	14	Karyo, SNP-A, NGS-CHOP, NGS-TBD	*TERC* NR_001566.2:n.303G>C het	Age 14:<br>*U2AF1* NM_006758.2:c.101C>T p.S34F (3%)<br><br>Age 15:<br>*U2AF1* NM_006758.2:c.101C>T p.S34F (11%)<br><br>Age 15.5:<br>*U2AF1* NM_006758.2:c.101C>T p.S34F (17%)	*MSH2* NM_000251.2:c.1906G>C p. A636P het
7569-690.01	10	Karyo, SNP-A, NGS-TBD	*TERC* NR_001566.2:n.303G>C het	None identified at ages 10, 11, and 12	*MSH2* NM_000251.2:c.1906G>C p.A636P het<br>*MSH2* NM_000251.2:c.1045C>G p.P349A het (VUS)
7569-691.01	44	Karyo, SNP-A, NGS-HMP, NGS-TBD	*TERT* c.G439T p.D147Y het	None identified at age 44	None identified
7569-695.01	51	Karyo, SNP-A, NGS-HMP, NGS-TBD	*TERC* r.376delG het (VUS)	None Identified at age 51	None identified

7569-703.01	67	Karyo, NGS-PS	*TERT* NM_198253.2:c.553C>T p.R185W het (VUS)	Age 67 (MDS):<br>*FANCC* NM_000136.3:c.276G>A p.W92* (14%)<br>*SF3B1* NM_012433.3:c.2098A>G p.K700E (41%)<br><br>Age 70 (MDS):<br>*FANCC* NM_000136.3:c.276G>A p.W92* (10%)<br>*SF3B1* NM_012433.3:c.2098A>G p.K700E (27%)	*GNAS* NM_080425.3:c.1658G>A p.G553D (49% VUS)
7569-708.01	65	Karyo, NGS-PS	*RTEL1* NM_001283009.1:c.2903G>C p.C968S het (VUS)	None identified at ages 65 and 67	*BRCA2* NM_000059.3:c.7580dup p.G2528Rfs*11
7569-713.01	72	Karyo, NGS-PS	*TERT* NM_198253.2:c.2072G>A p.R691H het (VUS)<br>*TERT* NM_198253.2:c.1457G>A p.R486H het (VUS)	Age 72.5:<br>*U2AF1* NM_001025203.1:c.101C>T p.S34F (30%)<br><br>Age 73:<br>*U2AF1* NM_001025203.1:c.101C>T p.S34F (31%)	*FANCM* NM_020937.3:c.5791C>T p.R1931* (VUS)<br>*PLCG2* NM_002661.4:c.5791C>T p.H193Q (VUS)
7569-716.01	60	Karyo, FISH, NGS-HMP, NGS-PS	None identified	Age 60:<br>del20q (66% by FISH)<br>46,XY,del(20)(q11.2q13.3)[20]<br><br>Age 61:<br>*ATM* NM_000051:c.4293dupT p.V1432Cfs*3 (17%)<br>*TP53* NM_000546:c.361_362insAAGT p.S121* (14%)<br>*ATM* NM_000051:c.8672G>A p.G2891D (16% VUS)<br><br>Age 65 (MDS):<br>*TP53* NM_000546:c.361_362insAAGT p.S121* (41%)<br>*ZMYM3* NM_005096.3:c.3085C>T p.Q1029* (86%)<br>*ATM* NM_000051:c.4293dupT p.V1432Cfs*3 (<7%)<br>*ATM* NM_000051:c.8672G>A p.G2891D (<7% VUS)<br>46,XY,del(20)(q11.2q13.3)[5]/46,XY[16]	*HFE* NM_000410.4:c.845G>A p.C282Y het<br>*HFE* NM_000410.4:c.187C>G p.H63D het<br>*CREBBP* NM_004380.2:c.500C>G p.A167G (VUS)
7569-717.01	71	Karyo, NGS-PS	*TERC* NR_001566.1:n.58G>C het (VUS)	Age 71:<br>*TP53* NM_001123385:c.755T>G p.L252R (VUS 16%)	*BCOR* NM_199015.2:c.179C>T p.T60M (VUS)<br>*BRINP3* NM_000546.5:c.1816C>A p.Q606K (VUS)
7569-721.01	32	Karyo, SNP-A, NGS-PS	*TERT* NM_198253.2:c.2080G>A p.V694M het (VUS)<br>*RTEL1* NM_001283009.2:c.2652+5G>A p.? het (VUS)	None identified at age 32	*CALR* NM_004343.3:c.69C>A p.F23L (VUS)<br>*ZRSR2* NM_005089.3:c.361G>A p.E121K (VUS)
7569-723.01	65	Karyo, NGS-PS	*TERT* NM_198253.3:c.3083A>C p.N1028T (VUS)	Age 65: *ATM* NM_000051.3:c.9022C>T p.R3008C (42%)	*BTK NM_001287344.1:c.996G>A p.? (100% VUS)*<br>*IL7R NM_002185.3:c.701G>A p.S234N (47% VUS)*<br>*NOTCH1 NM_017617.4:c.4025G>A p.G1342D (47% VUS)*
7569-737.01	37	Karyo, SNP-A, NGS-PS	*TERT* NM_198253.3:c.2105C>T p.P702L (likely pathogenic)	None	*ABL1* NM_007313.3:c.1990G>T p.A664S (48%)<br>*ERCC4* NM_005236.2:c.1630T>C p.F544L (50%)

7569-741.01	39	Karyo, SNP-A, NGS-PS, WGS	*TERT* NM_198253.2:c.2459T>G p.I820S (VUS)	Age 39 (MDS):<br>*U2AF1* NM_001025203.1:c.101C>T p.S34F (43%)<br>46,XY,t(5;9)(p15.3;q32)(*LINC02226::SNX30*)[7]/46,idem,del(20)(q11.2q13.1)[17]<br><br>Age 39.25 (MDS):<br>*U2AF1* NM_001025203.1:c.101C>T p.S34F (44%)<br>46,XY,add(9)(q22)[7]<br>arr[GRCh37] 20q11.22q13.13(32467139_48739342)x1-2 (15-20%)<br>arr[GRCh37] 21q22.12(36166651_36387807)x1-2 (15-20%)<br>arr[GRCh37] Xq26.2q26.3(132890802_134034459)x0-1 (15-20%)<br><br>Age 39.5 (MDS):<br>*U2AF1* NM_001025203.1:c.101C>T p.S34F (52%)<br>46,XY,t(5;9)(p15.3;q32)(*LINC02226::SNX30*)c,del(20)(q11.2q13.1)[20]"<br>arr[GRCh37] 20q11.22q13.13(32467139_48739342)x1-2 (40-45%)<br>arr[GRCh37] 21q22.12(36166651_36387807)x1-2 (40-45%)<br>arr[GRCh37] Xq26.2q26.3(132890802_134034459)x0-1 (40-45%)	*IDH2* NM_002168.3:c.652G>T p.G218C (VUS)
7569-742.01	61	Karyo, NGS-PS	None identified	None identified at age 61	*RAD21* NM_006265.3:c.1352T>G p.L451R (VUS)
7569-755.01	29	Karyo, NGS-PS	*TERT* NM_198253.3:c.2516C>T p.T839M het (VUS)	None identified at age 29	None identified
7569-757.01	52	Karyo, NGS-PS	*TERT* NM_198253.2:c.3170G>A p.G1057E het (VUS)	None identified at age 52	None identified
7569-759.01	69	Karyo	None identified	None identified at age 69	None identified
BCH-1	16	Karyo, NGS-RHP	*TERC* NR_001566.1:r.319G>A het	Age 12-18: Negative karyotype<br><br>Age 14: Negative NGS<br><br>Age 16 (*TERC* 37.8%):<br>*ATM* ENST00000278616.4:c.9022C>A p.R3008S (2.1%)<br>*ATM* ENST00000278616.4:c.3836_3837insGAAAAGTCTT p.L1283Efs*22 (1.4%)<br><br>Age 17 (*TERC* 27.3% then 34.1%):<br>*ATM* ENST00000278616.4:c.9022C>A p.R3008S (1.7% then 0.9%)<br>*ATM* ENST00000278616.4:c.3836_3837insGAAAAGTCTT p.L1283Efs*22 (1.1% then 0%)<br><br>Age 18: (*TERC* 37.8%)<br>*ATM* ENST00000278616.4:c.9022C>A p.R3008S (0.3%)<br>*ATM* ENST00000278616.4:c.3836_3837insGAAAAGTCTT p.L1283Efs*22 (0.1%)	None identified
PENN_OSUMC 01	37	NGS-O	*TERC* n.184_199delins16 (VUS)<br>*ZCCHC8* NM_017612.5:c.280A>T p.I94L (VUS)	Age 37:<br>*ATM* NM_000051.3:c.2572_2573ins76 p.F858fs*6 (12.8%)<br>*ATM* NM_000051.3:c.6698delT p.I2233fs* (36.5%)	None identified

Penn-DC01	56	Karyo, SNP-A, NGS-HMP, NGS-TBD	*TERC* NR_001566.1:n.72C>G (20% - mosaic, likely pathogenic)	Age 54: Normal karyotype<br>Age 57: Normal karyotype, no somatic variants<br>Age 56:<br>cnLOH 3q21.3q29 in 50%<br>Normal karyotype, no somatic variants<br>Age 58: Normal karyotype, no somatic variants<br>Age 60: Normal karyotype, no somatic variants	*HFE* NM_000410.4:c.845G>A p.C282Y het<br>*HFE* NM_000410.4:c.187C>G p.H63D het<br>*TET2* NM_001127208.2:c.3609C>G p.S1203R (VUS)
Penn-DC02	28	Karyo, NGS-TBD	*DKC1* NM_001363:c.1255T>A p.Y419N hem	Age 28 (MDS):<br>*TP53* NM_000546:c.514G>T p.V172F (54%)<br>39~40,XY, or -Y,-5,-7,add(7)(q22),add(9)(q34),-10, add(12)(p11.2),add(17)(p11.2),-18,-20,-21,-22,+12mar[cp6]<br>FISH positive for del5q31(16%), 7q31/D7Z1(32%), and 20q12(16%)<br><br>Age 28.8 (MDS)<br>39,X,-Y,-5,add(7)(q22),add(9)(q34),-10,add(12)(p11.2),add(17)(p11.2),-18,-20,-21,-22,+mar[1]/46,XY[1]	None identified
Penn-DC03	70	NGS-PS	*ACD* NM_001082486.1:c.137del p.R46Lfs*38 het (VUS)	Age 70:<br>*PPM1D* NM_003620.3 p.D509Gfs*6, c.1524_1525dup (3%)<br><br>Age 71.5:<br>*DNMT3A* NM_022552:c.855+1G>A p.? (<7%)<br>*PPM1D* NM_003620.3 p.D509Gfs*6, c.1524_1525dup (6%)	None identified
Penn-DC04	55	Karyo, NGS-HMP, NGS-PS	*RTEL1* NM_001283009:c.2098C>T p.AR700W het (VUS)	Age 55 (MDS):<br>*PTPN11* NM_002834:c.181G>T p.D61Y (6%)<br>*U2AF1* NM_001025203:c.101C>T p.S34F (40%)<br><br>Age 56 (AML):<br>*FLT3* NM_004119.3:c.1770_1793dup p.Y597_E598insDYVDFREY (31%)<br>*U2AF1* NM_001025203:c.101C>T p.S34F (35%)<br>*WT1* NM_024426.6:c.1151_1158dup p.A387Yfs*70 (23%)<br>*XPO1* NM_003400.3:c.2170C>T p.L724F (34% VUS)	*HFE* NM_000410.4:c.187C>G p.H63D het
Penn-DC05	52	Karyo, FISH, NGS-HMP	*DKC1* NM_001363.3:c.915+10 G>A hem<br>*RTEL1* NM_032957.4:c.2980 C>T p.Q994X het	Age 52:<br>46,XY,psu dic(12;7)(p13;q22),+add(19)(q13.1)[17]/46,XY[3]<br><br>Age 57<br>46,XY,psu dic(12;7)(p13;q22),+add(19)(q13.1)[17]/46,XY[2]<br>No somatic variants	None identified
Penn-DC06	33	Karyo, SNP-A, NGS-PS	*ZCCHC8* NM_017612.4:c.337G>A p.E113K het	Age 33:<br>*PPM1D* NM_003620.3:c.1535dup p.N512Kfs*16 (2%)<br>*ATM* NM_000051.3:c.6059G>A p.G2020D (7% VUS)<br>46,X,der(Y)t(Y;1)(q12;q21)[8]/46,XY[12]<br>arr[GRCh37] 1q21.1q44(143932349_249224684)x2-3 (10%)	*BRCA2* NM_000059.3c.88A>C p.N30H (50% VUS)<br>*IDH2* NM_002168.3:c.1214C>T p.T405M (54% VUS)<br>*PLCG2* NM_002661.4:c.1916A>T p.N639I (57% VUS)
Penn-DC08	60	Karyo	None identified	None identified at age 60	None identified
Penn-DC09	62	Karyo	None identified	None identified at age 62	None identified
Penn-DC10	36	Karyo, NGS-PS	*RTEL1* NM_001283009.1:c.2903G>C p.C968S het (VUS)	None identified at age 36	*BRCA2* NM_000059.3:c.10089A>G p.I3363M (49% VUS)

Penn-DC11	21	Karyo, SNP-A, NGS-PS	*SON* c.2365delA p.S789Afs het<br>*TERT* c.544A>T p.T182S het (VUS)	None identified at age 21	*KCNH2* NM_000238.4c.2863C>G p.L955V
Penn-DC12	55	Karyo, SNP-A, NGS-Neo, NGS-RHP	None identified	Age 55 (MDS):<br>*ASXL1* NM_015338:c.1934dup p.G646Wfs*12 (8.6%)<br>*ASXL1* NM_015338.5:c.3374C>T p.A1125V (VUS 30.5%)<br>*CUX1* NM_001202543.2:c.4214G>A p.G1405D (VUS 27.5%)<br>*U2AF1* NM_001025203.1:c.101C>T p.S34F (36.6%)<br>*U2AF1* NM_001025203.1:c.101C>A p.S34Y (4.0%)<br>(*BRCC3*, *RAD21* not assessed)<br><br>Age 55.8 (MDS):<br>*ASXL1* ENST00000306058.4:c.1934dup p.G646Wfs*12 (2.9%)<br>*ASXL1* ENST00000306058.4:c.3374C>T p.A1125V (VUS 43%)<br>*BRCC3* ENST00000369462.1:c.800-1G>A p.? (2.6%)<br>*RAD21* ENST00000297338.2:c.1162-5_1162-2delAATA p.? (1.1%)<br>*U2AF1* ENST00000291552.4:c.101C>T p.S34F (41.9%)<br>*U2AF1* ENST00000291552.4:c.101C>A p.S34Y (2.1%)	*ATM* NM_000051.3:c.6481C> p.R2161C het (VUS)
Penn-DC14	58	Karyo, SNP-A, NGS-PS	*TERT* NM_198253.2:c.2080G>A p.V694M	Age 58:<br>*TERT* NM_198253.2:c.-57A>C (23-33%)	*CREBBP* NM_004380.2:c.6656C>T p.A2219V (51% VUS)<br>*EGR2* NM_000399.4:c.906_911dup p.A308_A309dup (47% VUS)<br>*FANCM* NM_020937.3:c.4376C>G p.P1459R (48% VUS)<br>*PDGFRA* NM_006206.4:c.1285G>A p.G429R (46% VUS)
Penn-DC15	55	Karyo, NGS-PS	*TERT* NM_198253.2:c.2224C>T p.R742C het (VUS)<br>*PARN* NM_002582.3:c.994C>T p.Q332* het	Age 55:<br>*PPM1D* NM_003620.3:c.1714C>T p.R572* VAF 2%	None identified
Penn-DC16	65	Karyo, FISH, NGS-PS	*TERT* NM_198253.2:c.2329G>A p.V777M (VUS)	Age 65:<br>*TERT* NM_198253.2:c.-57A>C p.? (7%)<br>*TERT* NM_198253.2:c.-124C>T p.? (12%)	*SLX4* NM_032444.2:c.4259dup p.I1421Nfs*4
Penn-DC17	31	Karyo, SNP-A, FISH, NGS-HMP, NGS-PS	None identified	Age 31: *EZH2* NM_004456.4:c.392T>C p.I131T (<5% VUS)<br>Age 32: *EZH2* NM_004456.4:c.392T>C p.I131T (6% VUS)<br>Age 33: *EZH2* NM_004456.4:c.392T>C p.I131T (10% VUS)<br><br>Age 34:<br>*EZH2* NM_004456.4:c.392T>C p.I131T (<7% VUS)<br>*U2AF1* NM_001025203.1:c.101C>T p.S34F (37%)<br>46,XX,+1,der(1;7)(q10;p10)[2]/46,XX[18]<br><br>Age 35.5:<br>*EZH2* NM_004456.4:c.392T>C p.I131T (8% VUS)<br>*U2AF1* NM_001025203.1:c.101C>T p.S34F (15%)<br>46,XX,+1,der(1;7)(q10;p10)[5]/46,XX[15]<br><br>Age 36:<br>*EZH2* NM_004456.4:c.392T>C p.I131T (<7% VUS)<br>*U2AF1* NM_001025203.1:c.101C>T p.S34F (18%)<br>46,XX[20]	*PRPF40B* NM_001031698.2:c.302C>T p.P101L (51% VUS)<br>*CD79B* NM_001039933.2:c.392C>T p.S131L (50% VUS)

Penn-DC18	77	Karyo, NGS-PS	*RTEL1* NM_001283009.1:c.3762_3763delinsA p.F1255Sfs*109 het	None identified at age 77 years	*PALB2* NM_024675.3:c.908T>C p.L303P (46% VUS)<br>*SLX4* NM_032444.2:c.5260G>A p.E1754K (50% VUS)
Penn-DC19	67	Karyo, NGS-PS	*RTEL1* NM_001283009.1:c.2920C>T p.Arg974* het	Age 67:<br>*ATM* NM_000051.3:c.9156G>A p.W3052* (40%)<br>*TET2* NM_001127208.2:c.513C>A p.C171* (42%)<br><br>Age 70:<br>*ATM* NM_000051.3:c.9156G>A p.W3052* (41%)<br>*TET2* NM_001127208.2:c.513C>A p.C171* (46%)	*CIITA* NM_001286402.1:c.2774C>A p.S925Y (47% VUS)<br>*KLF2* NM_016270.3:c.361G>A p.V121I (52% VUS)
Penn-DC20	72	Karyo, NGS-PS	*TERT* NM_198253.2:c.336dup p.E113Rfs*79 het	Age 72:<br>*PPM1D* NM_003620.3:c.1434C>A p.C478* (3.7%)<br>*PPM1D* NM_003620.3:c.1567del p.A523Pfs*16 (1.2%)	None identified
Penn-DC21	68	Karyo, NGS-PS	*RTEL1* NM_001283009.1:c.2233G>C p.V745L het (VUS)	Age 68:<br>*TERT* NM_198253.3:c.-124C>T p.? (8%)	*BRAF* NM_004333.6:c.1024A>G p.I342V (52% VUS)
Penn-DC22	62	SNP-A	*TERC* NR_001566.1:r.35C>G het	Age 62:<br>*TERT* NM_198253.3:c.-124C>T p.? (<7%)	*CARD11* NM_001324281.1:c.2627A>C p.E876A (50% VUS)
TBD_DF_021	54	Karyo, NGS-RHP	*TERC* ENST00000602385:n.112C>G het	Age 54 (MDS):<br>46,XY,del(20)(q11.2)[6]/46,XY[14]<br><br>Age 54.2 (MDS):<br>*U2AF1* ENST00000291552.4:c.101C>T p.S34F (37.1%)<br>*ASXL1* ENST00000306058.4:c.1926_1927insG p.G646Wfs*12 (14.9%)<br>*ATM* ENST00000278616.4:c.1819G>A p.V607I (49.3%)<br><br>Age 54.8 (MDS):<br>*U2AF1* ENST00000291552.4:c.101C>T p.S34F (40.2%)<br>*ASXL1* ENST00000306058.4:c.1926_1927insG p.G646Wfs*12 (31.7%)<br>*ATM* ENST00000278616.4:c.1819G>A p.V607I (43.1%)<br>Karyotype: 46,XY,del(20)(q11.2)[13]/46,XY[7]	
TBD_DF_025	70	Karyo, NGS-RHP	*TERC* ENST00000602385:n.62A>G het	Age 70 (MDS):<br>*BRCC3* ENST00000369462.1:c.756-1G>C (7%)<br>*TET2* ENST00000380013.4:c.5681C>T p.P1894L (22%)<br>*TET2* ENST00000380013.4:c.5172T>A p.Y1724* (17.4%)<br>*U2AF1* ENST00000291552.4:c.101C>T p.S34F (34.2%)<br>*CTCF* ENST00000264010.4:c.971G>A p.C324Y (19.8% VUS)<br>*EP300* ENST00000263253.7:c.1905_1906insAAA p.E635_K636insK (19.2% VUS)<br>Karyotype: 46,XY[cp20]	
TBD_DF_028	51	Karyo, NGS-RHP	*TERC* ENST00000602385:n.182G>C het	*PPM1D* ENST00000305921.3:c.1281G>A p.W427* (2.7%)<br>Karyotype: 46,XY[20]	
TBD_DF_032	58	Karyo, NGS-RHP	*TERC* ENST00000602385:n.211C>T het	Age 32 (MDS)<br>*TP53* ENST00000420246.4:c.613T>G p.Y205D (14.1%)<br>*TP53* ENST00000420246.4:c.614A>G p.Y205C (13.3%)<br>*CTCF* ENST00000264010.4:c.1016G>T p.R339L (7.6% VUS)<br>45,XX,+X,der(4)t(4;12)(p14;q13)[3],der(5)t(5;7)(q15;p13),-12[7],-18,-18[7],-21[7],+1-4mar[cp11]/46,XX[9]	

TBD_DF_091	58	Karyo, NGS-RHP	*TERC* ENST00000602385:n.303G>C het	*Age 58:*<br>*ATM* ENST00000278616.4:c.6808-1G>C p.? (1.8%)<br>*ATM* ENST00000278616.4:c.9022C>T p.R3008C (0.6%)<br>*TP53* ENST00000420246.4:c.488A>G p.Y163C (1.2%)<br>*TP53* ENST00000420246.4:c.536A>G p.H179R (0.6%)<br>Karyotype: 46,XX[20]<br><br>Age 59:<br>*ATM* ENST00000278616.4:c.6808-1G>C p.? (2.1%)<br>*ATM* ENST00000278616.4:c.9022C>T p.R3008C (0.5%)<br>*TP53* ENST00000420246.4:c.488A>G p.Y163C (1.1%)<br>*TP53* ENST00000420246.4:c.536A>G p.H179R (0.5%)	
TBD_DF_092	27	NGS-RHP	*TERC* ENST00000602385:n.54_57delAACT het	*Age 27:*<br>*ATM* ENST00000278616.4:c.8614C>A p.H2872N (3.4%)<br><br>Age 28:<br>*ATM* ENST00000278616.4:c.8614C>A p.H2872N (2.8%)	
TBD_DF_094	35	NGS-RHP	*TERC* ENST00000602385:n.37A>T het	None identified at age 35	
TBD_DF_117	57	NGS-RHP	*TERC* ENST00000602385:n.35C>A het	None identified at age 57	
TBD_DF_128	31	NGS-RHP	*TERC* ENST00000602385:n.35C>A het	None identified at age 31	
TBD_DF_132	48	Karyo, NGS-RHP	*TERC* ENST00000602385:n.35C>T het	Age 48 (MDS):<br>46,XX,del(7)(q?22q?32)[4]//46,XY[16]	
TCH01	20	Karyo, NGS-TCH	*TERT* NM_198253.2:c.2078T>G p.F693C het (likely pathogenic)<br>*RTEL1* NM_032957:c.1261C>G p.O421E het<br>*WRAP53* NM_018081:c.407C>G p.P136R het	None identified at age 20	*PSTPIP1* NM_0039878:c.748G>A p.E250K het
TCH02	13	Karyo, NGS-TCH	*TERT* NM_198253.2:c.2006G>C p.R669P het (likely pathogenic)	None identified at age 13	*APC* NM_000038.5:c.5363G>A p.R1788H (44% VUS)<br>*ATM* NM_000051.3:c.4981C>T p.H1661Y (46% VUS)
TCH03	8	Karyo, NGS-TCH	*DKC1* NM_001363.4:c.1259+5G>C p.? hem (likely pathogenic)	None identified at age 8	*CUX1* NM_181552.3:c.2068C>G p.P690A (49% VUS)
TCH04	7	Karyo, NGS-TCH	None identified	None identified at age 7	*ALK* NM_004304.4:c.3601G>T p.G1201W (49% VUS)<br>*LEF1* NM_016269.4:c.433G>T p.V145L (51% VUS)<br>*ZFHX3* NM_006885.3:c.3424C>T p.R1142C (48% VUS)
TCH05	9	Karyo, SNP-A, NGS-TCH	*DKC1* NM_001363.4:c.1476+1G>C p.? hem (likely pathogenic)	None identified at age 9	*SYNE1* NM_182961.3:c.2630A>G p.Q877R (48% VUS)<br>*ZFHX3* NM_006885.3:c.3424C>T p.R1142C (48% VUS)
TCH06	20	Karyo, SNP-A, NGS-TCH	*DKC1* NM_001363:c.1259G>A p.S420N hem (likely pathogenic)<br>*WRAP53* NM_018081:c.230C>T p.T77I het (VUS)	None identified at age 20	*FAT1* NM_005245.3:c.291A>G p.E971G (50% VUS)<br>*FAT4* NM_024582.4:c.94445G>A p.A3149T (49% VUS)<br>*TET1* NM_030625.2:c.280C>T p.P94S (54% VUS)<br>arr[GRCh37] 8p21.1(27,722.309_27,856,331]x1

TCH07	15	Karyo, SNP-A, NGS-TCH	*TERT* NM_198253.3:c.2006G>C p.R669P het (likely pathogenic)<br>*RTEL1* NM_032957:c.2846C>G p.S949C het (VUS)	None identified at age 15	*ATM* NM_000051.3:c.4981C>T p.H1661Y (46% VUS)<br>*NOTCH3* NM_000435.2:c.5879A>G p.K1960R (53%)
TCH08	20	Karyo, NGS-TCH	None identified	Age 20:<br>46,XY,t(7;18)(p11.2;p11.3)[11]/46,XY[9].nuc ish(D5S23,EGR1)x2[200]/(D7Z1,D7S522)x2 [200]/(D8Z2x2)[200]/(D20S108x2)[200] and subsequent resolution	*BCL11B* NM_138576.2:c.911G>C p.G304A (50% VUS)<br>*BRAF* NM_004333.4:c.76G>A p.E26K (53%)<br>*KAT6B* NM_012330.3:c.3312_3314delAAA p.E1104_N1105delinsD (39% VUS)<br>*NOTCH2* NM_024408.3:c.646C>G p.Q216E (46%)
TCH09	22	Karyo, SNP-A, NGS-TCH	*PARN* NM_002582:c.272A>G p.Y91C het	Age 22: 46,XY,der(19)t(1;19)(q12;p13.3)[2]/46,XY[18].ish der(19)t(1;19)(ABL2+, TCF3+)	None identified
TCH10	15	Karyo, SNP-A, NGS-TCH	*TINF2* NM_001099274.3:c.844C>T p.R282C het	None identified at age 15	None identified
TCH11	14	Karyo, NGS-TCH	*TINF2* c.905_907delAGCinsGGTCATAT het	None identified at age 14	*KMT2D* NM_003482.3:c.6518C>T p.S2173L (50% VUS)
TCH12	10	Karyo	*DKC1* NM_001363.5:c.5C>T p.A2V hem	None identified at age 10	None identified
TCH13	4	Karyo	*TINF2* NM_001099274.3:c.805C>T p.Q269* het	None identified at age 4	None identified
TCH14	2	Karyo	*ACD* NM_001082486.2:c.505_507delGAG p.E169del<br>*ACD NM_001082486.2:*c.619delG p.D207Tfs∗22	None identified at age 2	None identified
TCH15	1	Karyo	*TINF2* NM_001099274.3:c.839del p.K280Rfs*36 het	None identified at age 1	None identified
TCH16	1	Karyo	*TERT* NM_198253.2:c.1700C>T p.T567M het	None identified at age 1	None identified
TCH17	9	Karyo	*DKC1* NM_001363.5:c.915+10G>A p.? hem	None identified at age 9	None identified
TCH18	17	Karyo	*TERT* NM_198253.3:c.2110C>T p.P704S het	None identified at age 17	None identified
TCH19	10	Karyo	None identified	None identified at age 10	None identified
TCH20	4	Karyo, SNP-A	*RTEL1* NM_032957:c.395G>A p.R132H het	None identified at age 4	None identified
UK 1241	13	NGS-PS	*TERC* NR_001566.1:n.54_57del	Age 13:<br>*KMT2C* NM_170606.2:c.2646del p.V884Wfs*29 (<7%)<br>*KMT2C* NM_170606.2:c.2647A>G p.K883E (<7% VUS)	None identified
UK 1455	3	NGS-PS	*TERC* NR_001566.1:n.178G>A	None identified at age 3	None identified
UK 1580	54	NGS-PS	*TERC* NR_001566.1:n.48A>G	None identified at age 54	*ATM* NM_000051.3:c.902-1G>T p.? (45%)
UK 1594	24	SNP-A, NGS-PS	*TERC* NR_001566.1:n.53_87del	Age 24:<br>Germline variant VAF 26%, no cnLOH seen on SNP-A<br>*PPM1D* NM_003620.3:c.1528_1529del p.Gln510Lysfs*17 (0.8%)	None identified
UK 2113	3	SNP-A, NGS-PS	*TERC* NR_001566.1:n.242C>T	None identified at age 3	None identified
UK 2867	52	SNP-A, NGS-PS	*TERC* NR_001566.1:n.54_57del	Age 52:<br>*FUBP1* NM_001303433.1:c.726del p.M242Ifs*32 (15%)<br>3q cnLOH	None identified

UK 3475	53	SNP-A, NGS-PS	*TERC* NR_001566.1:n.212C>G	None identified at age 53	None identified
UK 3692	47	SNP-A, NGS-PS	*TERC* NR_001566.1:n.323_336delinsAGACCC	Age 47:<br>*TET2* NM_001127208.2:c.996C>A p.C332* (4%)<br>*PPM1D* NM_003620.3:c.1559del p.Met521* (1.1%)	None identified
UK 440	10	SNP-A, NGS-PS	*TERC* NR_001566.1:n.408C>G	Age 10: *RAD50* NM005732.3:c.206A>G p.D69G (4% VUS)	*RAD50* NM_005732.3:c.94dup p.T32Nfs*16 (52%)
UK 4804	44	SNP-A, NGS-PS	*TERC* NR_001566.1:n.54A>G	Age 44 (MN):<br>*U2AF1* NM_001025203.1:c.101C>T p.S34F (VAF 12%)<br>*ASXL1* NM_015338.5:c.2053G>T p.G685* (5%)<br>*ETV6* NM_001987.4:c.1080G>A p.W360* (6%)<br>*NTRK3* NM_001012338.2:c.917G>C p.R306P (<7% VUS)<br>*PLCG2* NM_002661.4:c.1759 p.R587W (13% VUS)	None identified
UK 520	52	SNP-A, NGS-PS	*TERC* NR_001566.1:n.107_108delinsAG	Age 52:<br>3q cnLOH<br>*FOXL2* NM_023067.3:c.724G>T p.A242S (26% VUS)	None identified
UK 641	7	SNP-A, NGS-PS	*TERC* NR_001566.1:n.378_?del	None identified at age 7	None identified
UK 813	27	SNP-A, NGS-PS	*TERC* NR_001566.1:n.107_108delinsAG	Age 27: 3q cnLOH	*POLD1* NM_001308632.1:c.583C>T p.R195*
UK 850	42	SNP-A, NGS-PS	*TERC* NR_001566.1:n.96_97del	Age 42: 3q cnLOH	None identified
UW001	42	Karyo, NGS-Tempus, NGS-Mayo	*TERC* NR_001566.1:r.114_115delTT het	Age 42<br>46,XY,+1,der(1;21)(q10;q10)[19]/46,XY[1]<br>*TERT* NM_198253:c.-124C>T (3.9%)<br><br>Age 43<br>46,XY,+1,der(1;21)(q10;q10)[17]/46,XY[3]<br>*TERT* NM_198253:c.-124C>T (3.7%)	Age 42<br>*CEBPA* NM_004364:c.406G>A p.A136T (52% VUS)<br>*XRCC1* NM_006297:c.1713-5G>A (41% VUS)<br><br>Age 43<br>CEBPA NM_004364:c.406G>A p.A136T (56% VUS)<br>XRCC1 NM_006297:c.1713-5G>A (47% VUS)<br>CREBBP NM_004380:c.712G>C p.V238L (46% VUS)
UW002	27	Karyo, FISH	*RTEL1* NM_001283009.1:c.1274T>C p.I425T het (VUS)<br>*TERC* NM_001566.1:r.318A>C het (VUS)	None identified at ages 27 or 28	None identified
UW003	29	Karyo, WES	*TINF2* c.838A>G p.K280E NM_012461 het	Age 29<br>*POT1* NM_015450.3:c.282G>C p.Q94H (11% likely pathogenic)<br>*POT1* NM_015450.3:c.114C>G p.S38R (13% VUS)	*CHEK2* NM_007194.4:c.1563del p.R523Vfs (25% VUS)
UW004	42	Karyo, WES	*PARN* NM_002582.4:c.554+35G>A p.? het (VUS)	None identified at age 42	None identified
UW005	57	Karyo	*TERC* NM_001566.1:r.36C>T het	None identified at age 57	*DDX41* NM_016222.4:c.3G>A p.M1I Het
UW007	35	Karyo, NGS-UW	*PARN* NM_002582.3:c.1749_1750del p.E585Dfs*5 het	Age 35: 46,XX,t(15;17)(q24;q21)[10],46,XX[10]	None identified
UW008	66	Karyo, WES	*PARN* NM_002582.3:c.1749_1750del p.E585Dfs*5 het	None identified	None identified
UW009	63	Karyo	*TERT* NM_198253.2:c.2131-2A>G p.? het	None identified	None identified

UW010	69	Karyo, NGS-UW	None identified	Age 69: 46,XX,add(1)(p34)[12]/46,sl,del(13)(q12q14)[4]/46,XX[4]<br>*SF3B1* NM_012433.2:c.2098A>G p.K700E (45.8%)	*KIT* NM_000222.2:c.1621A>C p.M541L (51.3% VUS)<br>*NF1* NM_001042492.2:c.596T>A p.F199Y (50.1% VUS)
UW011	58	Karyo, WES	*RTEL1* NM_001283009.1:c.2956C>T p.R986* het	None identified	None identified
UW012	45	Karyo	*RTEL1* NM_001283009.1:c.2920C>T p.R974* het<br>*RTEL1* NM_001283009.1:c.245C>T p.P82L het (VUS)	Age 45: 46,XY,t(9;22)(q34;q11.2)[3]	None identified
UW013	55	Karyo, WES	*RTEL1* NM_001283009.1:c.2249G>T p.R750L het (VUS)<br>*RTEL1* NM_001283009.1:c.2651C>T p.P884L het (VUS)	None identified at age 55	None identified
UW014	65	Karyo, WES	*RTEL1* NM_001283009.1:c.2206_2208del p.D736del het (VUS)<br>*ACD* NM_001082486.1:c.1126G>A p.V376I het (VUS)	None identified at age 65	None identified
UW015	60	Karyo, WES	*RTEL1* NM_001283009.1:c.1675T>A p.F559I het (VUS)	None identified at age 60	*FANCM* NM_020937.2:c.1849C>G p.Q617E het (VUS)<br>*FANCM* NM_020937.2:c.538A>G p.I180V het (VUS)
UW016	52	Karyo, NGS-Mayo	*TERT* NM_198253.2:c.3107T>C p.I1036T het (VUS)	None identified at age 52	*MBD4* gain (VUS)<br>*FANCM* NM_020937.2:c.190G>T p.A64S het (VUS)<br>*CHEK2* NM_007194.3:c.911T>C p.M304T het (VUS)
UW017	33	WES	*TERT* NM_198253.3:c.172G>C p.V58L het (VUS)	None identified at age 33	None identified
UW018	69	WES	*RTEL1* NM_001283009.1:c.2587_2590delTCTG p.S863Rfs* het	None identified at age 69	None identified
UW019	51	WES	*RTEL1* NM_001283009.1:c.2988del p.T997Lfs* het	*U2AF1* NM_006758.3:c.101C>T p.S34F (18%)	None identified
UW020	21	WES	*TERT* NM_198253.3:c.2110C>T p.P704S het	None identified at age 21	None identified
UW021	70	WES	*RTEL1* NM_001283009.1:c.2587_2590delTCTG p.S863Rfs* het	None identified at age 70	None identified
UW022	28	WES	*RTEL1* NM_001283009.1:c.2956C>T P.R986* het	*BCORL1* NM_021946.5:c.145_155delinsAGGTAGGACGG p.P482_L485delinsQVGR (7% VUS)	None identified
UW023	31	WES	*TERT* c.2110C>T p.P704S NM_198253.3 het	None identified at age 31 years	None identified
UW024	71	WES	*TERT* NM_198253.3:c.1174_1175delCT p.L392Vfs* het<br>*ACD* NM_001082486.2:c.109G>A p.D37N het (VUS)	None identified at age 71 years	None identified
UW025	63	WES	*TERT* NM_198253.3:c.2379G>T p.E793D het (VUS)	None identified at age 63 years	*FANCM* NM_020937.4:c.5791C>T p.R1931* (48%)<br>*NTHL1* NM_002528.7:c.244C>T p.Q82* (53%)<br>*MUTYH* NM_001128425.2:c.167G>T p.G56V (50%)

UW026	68	WES	None identified	None identified at age 68 years	None identified
UW027	61	WES	None identified	None identified at age 61 years	None identified
UW028	43	WES	None identified	None identified at age 43 years	None identified
UW029	66	WES	None identified	None identified at age 66 years	None identified
UW030	78	WES	None identified	Age 78:<br>*ATM* NM_000051.4:c.8672G>A p.G2891D (15% VUS)	*FANCM* NM_020937.4:c.1892delA p.D631Vfs* (56%)<br>*ATM* NM_000051.4:c.68G>A p.R23Q (54% VUS)
UW032	50	WES	*PARN* NM_002582.4:c.338T>A p.I113N het (VUS)	None identified by age 50 years	None identified
UW033	53	karyo, NGS-Tempus	*TERT* NM_198253.2:c.2329G>A p.V777M het (VUS)	Age 53:<br>*XPO1* NM_003400:c.1630A>G p.I544V (28% VUS)<br>*DNMT3A* NM_022552:c.1922A>T p.D641V (25% VUS)	*PLCG2* NM_002661:c.50A>C p.Q17P (50% VUS)
UW034	64	karyo, NGS-Tempus	*RTEL1* NM_001283009.1:c.3791G>A p.R1264H het	*TERT* NM_198253.3:c.-124C>T p.? (26.2%)<br>*U2AF1* NM_006758.3:c.101C>A p.S34Y (3.1%)	*ERCC4* NM_005236.2:c.2395C>T p.R799W het<br>*MBD4* NM_003925.2:c.1163C>G p.P388R het (VUS)<br>*MN1* NM_002430:c.1550C>T p.P517L (48% VUS)<br>*KMT2C* c.13534C>A p.H4512N NM_170606 (48% VUS)
WU006-52	59	WES, NGS-WU	*TERC* n.206C>T het (VUS)	*DNMT3A* chr2:g.25457266T>C (3.8%)<br>*TP53* chr17:g.7577120C>T (11.69%)	None identified
WU008-40	65	WES, NGS-WU	*TERT* p.P627R	*BCORL1* chrX:g.129189835C>A (3.59%)<br>*PPM1D* chr17:g.58740383del (2.94%)<br>*ASXL1* chr20:g.31024320del (0.75%)<br>*CEBPB* chr20:g.48808127G>T (0.84%)	None identified
WU008-57	56	WES, NGS-WU	*RTEL1* p.Q397E	*PPM1D* chr17:g.58740444del (2.13%)	None identified
WU009-04	65	WES, NGS-WU	*TERT* p.K710N	None identified at age 65	None identified
WU010-20	59	WES, NGS-WU	*PARN* p.Y384C	None identified at age 59	None identified
WU010-25	68	WES, NGS-WU	*TERT* p.R466W	*RUNX1* chr21:g.36252869C>T (6.01%)	None identified
WU013-59	61	WES, NGS-WU	*PARN* p.Y91C	*CREBBP* chr16:g.3781420T>G (25%)	None identified
WU013-65	48	WES, NGS-WU	*RTEL1* p.R702H<br>*TERT* p.R698W	*TET2* chr4:g.106156036dup (5.36%)	None identified
WU015-03	60	WES, NGS-WU	*RTEL1* p.M652T	None identified at age 60	None identified
WU015-15	64	WES, NGS-WU	*RTEL1* p.C968S	None identified at age 64	None identified
WU015-34	75	WES, NGS-WU	*RTEL1* p.E180D	None identified at age 75	None identified
WUSTL420.1	1	HA	*TERT* c.2638G>A p.A880T<br>*TERT* c.1589C>T p.P530T (VUS)	Age 1: Skewed X-chromosome inactivation	None identified

HA, Humara Assay; WES, whole exome sequencing; NGS, next generation sequencing; SNP-A, single nucleotide polymorphism array; FISH, fluorescence in situ hybridization; Karyo, karyotype; NGS-TBD, Custom NGS panel (Supplemental Table S2); NGS-PS, NGS-Pennseq panel (Supplemental Table S3a); NGS-HMP, NGS-hematopoietic malignancies panel (Supplemental Table S3b); NGS-CHOP, NGS-CHOP panel (Supplemental Table S3c); NGS-TCH, Texas Children's Heme panel (Supplemental Table S3d); NGS-RHP, Dana Farber rapid heme panel (Supplemental Table S3e); NGS-WU, WashU panel (Supplemental Table S3g); NGS-O, Ohio State (Supplemental Table S3f); NGS-Neo, Neogenomics; NGS-S, Stanford heme panel; NGS-Onco, Oncometrix heme panel; NGS-Tempus, Tempus heme panel; NGS-UW, UWisconsin heme panel; NGS-Mayo, Mayo heme panel.

Supplemental Table S4: Somatic *ATM* variants

The complete table, containing information on population frequencies and in silico variant pathogenicity predictions, is available in a separate supplemental excel table (**Supplemental Table S4.xlsx**). A subset of that table with Clinvar IDs and pathogenicity classifications is here for ease of use.

Variant	Number Affected	Variant Type	Exonic Variant Classification	Pathogenicity	ClinVar ID	CLNALLE LEID	CLNSIG	Interpro_domain	COSMIC Genomic Variant ID
***ATM* NM_000051 c.2839-2G>A p.?**	1	splicing		P/LP	No entry				No entry
***ATM* NM_000051 c.6219_6256dup p.Y2086Sfs*9**	1	exonic	frameshift insertion	P/LP	No entry				No entry
***ATM* NM_000051 c.8495G>A p.R2832H**	1	exonic	nonsynonymous SNV	VUS	133638	137377	Uncertain_significance	Phosphatidylinositol 3-/4-kinase, catalytic domain|Protein kinase-like domain	COSV5374 9441
***ATM* NM_000051 c.8672G>A p.G2891D**	3	exonic	nonsynonymous SNV	VUS	216235	212915	Uncertain_significance	Phosphatidylinositol 3-/4-kinase, catalytic domain|Protein kinase-like domain	COSV5373 3895
***ATM* NM_000051.3 c.7629+1G>A p.?**	1	splicing		P/LP	581154	564334	Likely_pathogenic	.	COSV5372 7963
***ATM* NM_000051.3 c.7880A>G p.Y2627C**	1	exonic	nonsynonymous SNV	LP	220064	222119	Uncertain_significance	Protein kinase-like domain	COSV9958 8162
***ATM* NM_000051 c.4293dupT p.V1432Cfs*3**	1	exonic	frameshift substitution	P/LP	No entry				No entry
***ATM* NM_000051.3 c.9022C>T p.R3008C**	2	exonic	nonsynonymous SNV	P/LP	142187	151901	Pathogenic/Likely_path ogenic	Phosphatidylinositol 3-/4-kinase, catalytic domain	COSV5373 0980
***ATM* ENST00000278616. 4 c.9022C>A p.R3008S**	1	exonic	nonsynonymous SNV	LP	822905	810740	Conflicting_interpretatio ns_of_pathogenicity	Phosphatidylinositol 3-/4-kinase, catalytic domain	COSV5373 9432
***ATM* ENST00000278616. 4**	1	exonic	frameshift insertion	P/LP	No entry				No entry

c.3836_3837insGA AAAGTCTT p.L1283Efs*22									
ATM NM_000051.3 c.2572_2573ins76 p.F858fs*6	1	exonic	frameshift insertion	P/LP	No entry				No entry
ATM NM_000051.3 c.6698del p.I2233fs*	1	exonic	frameshift deletion	P/LP	No entry				No entry
ATM NM_000051.3 c.6059G>A p.G2020D	1	exonic	nonsynonymous SNV	VUS	826160	810544	Uncertain_significance	PIK-related kinase	COSV5373 0415
ATM NM_000051.3 c.9156G>A p.W3052*	1	exonic	stopgain	P/LP	947996			FATC domain|Phosphatidyl inositol 3-/4-kinase, catalytic domain	No entry
ATM ENST00000278616.4 c.6808-1G>C p.?	1	splicing		P/LP	924503	916089	Likely_pathogenic	.	No entry
ATM ENST00000278616.4 c.8614C>A p.H2872N	1	exonic	nonsynonymous SNV	VUS	No entry			Phosphatidylinositol 3-/4-kinase, catalytic domain|Phosphatidyl inositol 3/4-kinase, conserved site|Protein kinase-like domain	No entry
ATM ENST00000278616.4 c.1819G>A p.V607I	1	exonic	nonsynonymous SNV	VUS	236679	240934	Uncertain_significance	.	No entry

Pathogenicity classification: P, pathogenic; LP, likely pathogenic; VUS, variant of uncertain significance.

Supplemental Table S5: *ATM* variants, somatic status confirmation, and immunosuppressive and cytotoxic therapy exposures preceding identification

Patient ID	Age (years)	Acquired *ATM* Variants	Somatic Evidence	Exposures Preceding *ATM* Variant Identification
7569-629.01	60	*ATM* NM_000051: c.2839-2G>A p.?<br>*ATM* NM_000051: c.6219_6256dup p.Y2086Sfs*9<br>*ATM* NM_000051: c.8495G>A p.R2832H<br>*ATM* NM_000051: c.8672G>A p.G2891D	Absent in constitutional tissue (skin)	Steroids prior to lung transplant, and sirolimus and prednisone after lung transplant
7569-629.04	33	*ATM* NM_000051.3: c.7629+1G>A p.?<br>*ATM* NM_000051.3: c.7880A>G p.Y2627C	Absent in constitutional tissue (skin)	No immunosuppression/cytotoxic exposures
7569-716.01	60	*ATM* NM_000051: c.4293dupT p.V1432Cfs*3 (17%)<br>*ATM* NM_000051: c.8672G>A p.G2891D (16% VUS)	VAF ≤17%, subsequent drop in VAF to <7%.	Prednisone before lung transplant; prednisone, tacrolimus, and mycophenolate mofetil after lung transplant
7569-723.01	65	*ATM* NM_000051.3: c.9022C>T p.R3008C (42%)	Absent in constitutional tissue (lung explant and lymph node)	No immunosuppression/cytotoxic exposures
BCH-1	16	*ATM* ENST00000278616.4: c.9022C>A p.R3008S<br>*ATM* ENST00000278616.4: c.3836_3837insGAAAAGTCTT p.L1283Efs*22	VAF <3%, originally absent on NGS	No immunosuppression/cytotoxic exposures
PENN_OSUMC01	37	*ATM* NM_000051.3: c.2572_2573ins76 p.F858fs*6<br>*ATM* NM_000051.3: c.6698del p.I2233fs*	Absent in constitutional tissue (skin)	Mycophenolate mofetil; variants identified before lung transplant
Penn-DC06	33	*ATM* NM_000051.3: c.6059G>A p.G2020D	VAF 7%	No immunosuppression/cytotoxic exposures
Penn-DC19	67	*ATM* NM_000051.3: c.9156G>A p.W3052*	Presumed somatic with VAF 40%, similar to somatic *TET2* variant in same patient, and lower than heterozygous presumed germline variants in same patient.	No immunosuppression/cytotoxic exposures
TBD_DF_021	54	*ATM* ENST00000278616.4: c.1819G>A p.V607I	Absent in constitutional tissue (skin)	No immunosuppression/cytotoxic exposures
TBD_DF_091	58	*ATM* ENST00000278616.4: c.6808-1G>C p.?<br>*ATM* ENST00000278616.4: c.9022C>T p.R3008C	VAF <2%	No immunosuppression/cytotoxic exposures
TBD_DF_092	27	*ATM* ENST00000278616.4: c.8614C>A p.H2872N	VAF <2%	No immunosuppression/cytotoxic exposures
UW030	78	*ATM* NM_000051.4: c.8672G>A p.G2891D	15% VAF	No immunosuppression/cytotoxic exposures

Supplemental Table S6: Custom panel including DNA damage response, senescence, and cell cycle genes (NGS-TBD)

ACD	*AHCY*	*AHNAK2*	*AKR1B1*	*AKT1*	*AP2M1*	*ARF1*	*ARID3A*	*ASXL1*
ATM	*ATR*	*ATRIP*	*ATXN10*	*AURKA*	*BABAM1*	*BARD1*	*BBC3*	*BCL6*
BCOR	*BCORL1*	*BMI1*	*BRCA1*	*BRCA2*	*BRD7*	*BRF1*	*BRIP1*	*BUB1*
CBX7	*CDC25A*	*CDC25B*	*CDC25C*	*CDK1*	*CDK6*	*CDKN1A*	*CDKN1C*	*CDKN2A*
CDKN2B	*CDKN2C*	*CHEK1*	*CHEK2*	*CHUK*	*CIAO2B*	*CLSPN*	*CNOT6*	*CRISPLD2*
CSN2	*CSNK1A1*	*CSNK1E*	*CSNK2A1*	*CTC1*	*CXCR2*	*DCLRE1C*	*DKC1*	*DLX2*
DLX6	*DNMT3A*	*DUSP1*	*DUSP3*	*DUSP4*	*DUSP11*	*DUSP13*	*E2F1*	*E2F2*
E2F3	*E2F7*	*EHBP1*	*EHMT1*	*EHMT2*	*ENY2*	*EPHA3*	*ERCC1*	*ERCC2*
ERG	*ETV1*	*ETV6*	*EXO1*	*EXOSC3*	*EZH2*	*FANCF*	*FBXO5*	*FBXO31*
FBXW7	*FEN1*	*FOXO4*	*GADD45A*	*GATA2*	*GATA4*	*GIT2*	*H2AX*	*HAS1*
HMGB1	*HMGN1*	*HMGN2*	*HOTAIR*	*HRAS*	*IDH1*	*IDH2*	*IGFBP5*	*IKBKB*
IL6	*IL6R*	*ING5*	*IRAK2*	*IRAK3*	*ITGB3*	*ITSN2*	*JAK1*	*JAK2*
JUN	*KAT5*	*KAT6A*	*KAT6B*	*KAT8*	*KCNA1*	*KDM2B*	*KDM4A*	*KDM4B*
KDM4D	*KDM5A*	*KDM5B*	*KDM6B*	*KLF4*	*KLF11*	*KRAS*	*KSR2*	*LATS1*
LAYN	*LIG3*	*LMNB1*	*LTBP2*	*LTBP3*	*MAD2L2*	*MAP2K3*	*MAP4K1*	*MAPK8*
MAPK14	*MAPKAPK2*	*MCTP1*	*MDC1*	*MDK*	*MDM2*	*MDM4*	*MIR9-1*	*MIR9-2*
MIR18A	*MIR22*	*MIR26B*	*MIR29C*	*MIR34A*	*MIR125A*	*MIR125B1*	*MIR125B2*	*MIR128-1*
MIR137	*MIR138-1*	*MIR138-2*	*MIR141*	*MIR181A1*	*MIR181A2*	*MIR181B1*	*MIR181B2*	*MIR192*
MIR194-1	*MIR194-2*	*MIR195*	*MIR203A*	*MIR203B*	*MIR205*	*MIR210*	*MIR212*	*MIR217*
MIR335	*MIR424*	*MIR449A*	*MIR449B*	*MIR451A*	*MIR494*	*MIR519A1*	*MIR519A2*	*MIR605*
MIR668	*MIR1827*	*MIRLET7E*	*MLH1*	*MORF4L1*	*MRE11*	*MSH2*	*MTOR*	*MYC*
MYLK	*NAF1*	*NBN*	*NEK1*	*NEK4*	*NFKB2*	*NHP2*	*NOP10*	*NOTCH1*
NRAS	*NUAK1*	*OXTR*	*PALB2*	*PANDAR*	*PARN*	*PARP1*	*PARP2*	*PAXIP1*
PBRM1	*PHF6*	*PIAS1*	*PIF1*	*PIK3C2A*	*PINX1*	*PLA2R1*	*POT1*	*PPM1D*
PPP1CA	*PPP4R2*	*PPP4R3A*	*PRKACA*	*PRKACB*	*PTEN*	*RAB3B*	*RAB14*	*RAD17*
RAD50	*RAD51*	*RASGEF1B*	*RB1*	*RBBP8*	*REST*	*RFC1*	*RHOA*	*RIF1*
RNF8	*RNF168*	*RPA1*	*RTEL1*	*RUNX1*	*RUNX3*	*RUVBL1*	*RUVBL2*	*SERPINE1*
SETBP1	*SF3B1*	*SIK1*	*SIRT1*	*SMAD2*	*SMG1*	*SOCS1*	*SOCS3*	*SPRY2*
SRSF2	*STAG2*	*STAT1*	*STAT3*	*STK11*	*STN1*	*SUZ12*	*TBX2*	*TBX3*
TCF20	*TELO2*	*TEN1*	*TENT4B*	*TERC*	*TERF1*	*TERF2*	*TERF2IP*	*TERT*
TET2	*TGFB1*	*TGFBR1*	*TINF2*	*TLR2*	*TLR6*	*TLR9*	*TMEM9B*	*TNFRSF17*
TNFRSF19	*TOP1*	*TOPBP1*	*TP53*	*TP53BP1*	*TP53I13*	*TRAF6*	*TRIM28*	*TTI1*
TTI2	*U2AF1*	*UBE2N*	*UBTD1*	*UCHL1*	*USP15*	*USP28*	*WRAP53*	*WT1*
XRCC1	*XRCC5*	*ZBTB7A*	*ZBTB48*	*ZCCHC8*	*ZRSR2*	*ZSCAN9*		

Green: TBD genes; Pink: MDS-associated genes; Blue: genes associated with DDR and cell senescence. Sensitivity of detection of VAF>4%. This panel had a coverage of a median 321 reads (ranging from 19 to 3410 reads) over the promoter region of TERT gene (chr:1293230-1295280, hg38). This region was manually reviewed for potential variants in the hotspot areas -124 C/T variant (C228T, chr5:1,295,228 C>T) or -146 C/T variant (C250T, chr5: 1,295,250 C>T).

Supplemental Table S7: Clinical NGS panels

Supplemental Table S7a: PennSeq hematologic malignancies panel (NGS-PS)

ABL1	*ASXL1*	*ATM*	*B2M*	*BCL2*	*BCOR*	*BCORL1*
BIRC3	*BRAF*	*BRCA1*	*BRCA2*	*BRINP3*	*BRIP1*	*BTK*
CALR	*CARD11*	*CBL*	*CD79A*	*CD79B*	*CDKN2A*	*CEBPA*
CIITA	*CREBBP*	*CSF1R*	*CSF3R*	*CXCR4*	*DDX3X*	*DDX41*
DICER1	*DNMT3A*	*EGR2*	*ERCC4*	*ETV6*	*EZH2*	*FANCA*
FANCC	*FANCD2*	*FANCE*	*FANCF*	*FANCG*	*FANCL*	*FANCM*
FBXW7	*FLT3*	*GATA2*	*GNA13*	*GNAS*	*HNRNPK*	*ID3*
IDH1	*IDH2*	*IKZF1*	*IL7R*	*JAK2*	*JAK3*	*KIT*
KLF2	*KLHL6*	*KRAS*	*MAP2K1*	*MAPK1*	*MIR142*	*MPL*
MYC	*MYCN*	*MYD88*	*NF1*	*NFKBIE*	*NOTCH1*	*NOTCH2*
NPM1	*NRAS*	*PALB2*	*PDGFRA*	*PHF6*	*PLCG1*	*PLCG2*
POT1	*PRPF40B*	*PTEN*	*PTPN11*	*RAD21*	*RAD51*	*RAD51C*
RHOA	*RIT1*	*RPS15*	*RRAGC*	*RUNX1*	*SETBP1*	*SF1*
SF3A1	*SF3B1*	*SLX4*	*SMC1A*	*SOCS1*	*SRSF2*	*STAG2*
STAT3	*STAT5B*	*TBL1XR1*	*TCF3*	*TERT*[1]	*TET2*	*TNFAIP3*
TNFRSF14	*TP53*	*TPMT*	*TRAF3*	*U2AF1*	*U2AF2*	*WT1*
XPO1	*XRCC2*	*ZMYM3*	*ZRSR2*	**PPM1D*		

**PPM1D* was additionally analyzed with the panel for this research study.

Sensitivity of detection VAF of 4% for initial variant calls, and lower for subsequent calls. Hotspots in *U2AF1* S34 and *PPM1D* exon 6 were manually reviewed with a sensitivity of detection to <1%.

TERT promoter region: The Pennseq panel covered the promoter region of TERT gene (chr:1293230-1295280, hg38), with the depth of coverage ranging from 500-4278). The minimum criteria for the depth of coverage in was 200-250 reads. The two genomic locations of the classic TERT promoter variants c.-146C>T (chr5:g.1295135G>A, C250T) and c.124C>T (chr5:g.1295113G>A, C228T) had a mean coverage of 1681X (± 426 SD) with a median 1499X.

Supplemental Table S7b: Penn original hematologic malignancy panel (NGS-HMP)

ABL1	*ASXL1*	*ATM*	*BCOR*	*BCORL1*	*BIRC3*	*BRAF*
CALR	*CBL*	*CDKN2A*	*CEBPA*	*CSF1R*	*CSF3R*	*DDX3X*
DNMT3A	*ETV6*	*EZH2*	*FAM5C*	*FBXW7*	*FLT3*	*GATA2*
GNAS	*HNRNPK*	*IDH1*	*IDH2*	*IL7R*	*JAK2*	*KIT*
KLHL6	*KRAS*	*MAP2K1*	*MAPK1*	*MIR142*	*MPL*	*MYC*
MYCN	*MYD88*	*NF1*	*NOTCH1*	*NOTCH2*	*NPM1*	*NRAS*
PDGFRA	*PHF6*	*POT1*	*PRPF40B*	*PTEN*	*PTPN11*	*RAD21*
RIT1	*RUNX1*	*SETBP1*	*SF1*	*SF3A1*	*SF3B1*	*SMC1A*
SRSF2	*STAG2*	*TBL1XR1*	*TET2*	*TP53*	*TPMT*	*U2AF1*
U2AF2	*WT1*	*XPO1*	*ZMYM3*	*ZRSR2*		

Sensitivity of detection VAF >4%. TERT promoter region is not covered.

Supplemental Table S7c: CHOP comprehensive hematological cancer panel (NGS-CHOP)

Sequenced Genes	*ABL1*	*ASXL1*	*ASXL2*	*ATRX*	*BCL11B*	*BCL6*	*BCOR*
	BCORL1	*BRAF*	*BRINP3*	*CALR*	*CBL*	*CCND3*	*CD79A*
	CD79B	*CDC25C*	*CDKN2A*	*CDKN2B*	*CEBPA*	*CREBBP*	*CRLF2*
	CSF1R	*CSF3R*	*CTCF*	*DDX41*	*DNM2*	*DNMT1*	*DNMT3A*
	DOT1L	*EBF1*	*EED*	*ELANE*	*EP300*	*EPOR*	*ERG*
	ESR1	*ETNK1*	*ETS1*	*ETV6*	*EZH2*	*FBXW7*	*FLT3*
	GATA1	*GATA2*	*GATA3*	*HNRNPK*	*HRAS*	*IDH1*	*IDH2*
	IKZF1	*IKZF3*	*IL7R*	*JAK1*	*JAK2*	*JAK3*	*KDM6A*
	KIT	*KRAS*	*LEF1*	*LYL1*	*KMT2A*	*KMT2C*	*KMT2D*
	MAP2K1	*MPL*	*MSH2*	*MSH6*	*MYB*	*MYD88*	*NF1*
	NOTCH1	*NPM1*	*NRAS*	*NSD1*	*NT5C2*	*NUDT15*	*PAX5*
	PDGFRA	*PHF6*	*PIK3R1*	*PRPF40B*	*PRPF8*	*PTEN*	*PTPN11*
	RAD21	*RB1*	*RELN*	*RPL10*	*RETEI1*	*RUNX1*	*SETBP1*
	SETD2	*SF1*	*SF3A1*	*SF3B1*	*SH2B3*	*SMC1A*	*SMC3*
	SRSF2	*STAG2*	*STAT3*	*SUZ12*	*TAL1*	*TCF3*	*TERT*
	TET2	*TINF2*	*TLX1*	*TLX3*	*TP53*	*TPMT*	*U2AF1*
	U2AF2	*UBA2*	*USH2A*	*USP7*	*WHSC1*	*WT1*	*ZRSR2*
Fusion Partner	*ABL1*	*ABL2*	*AKT3*	*ALK*	*ARHGAP26*	*AXL*	*BCL2*
	BCL6	*BCR*	*BRAF*	*BRD3*	*BRD4*	*CAMTA1*	*CBFA2T3*
	CBFB	*CCNB3*	*CCND1*	*CIC*	*CREBBP*	*CRFL2*	*CSF1R*

	DNAJB1	*DUSP22*	*EGFR*	*EPC1*	*EPOR*	*ERG*	*ESR1*
	ESRRA	*ETV1*	*ETV4*	*ETV5*	*ETV6*	*EWSR1*	*FGFR1*
	FGFR2	*FGFR3*	*FGR*	*FOXO1*	*FUS*	*GLI1*	*GLIS2*
	HMGA2	*IL2RB*	*IL3*	*IL3RA*	*INSR*	*JAK2*	*JAZF1*
	KAT6A	*KMT2A*	*MALT1*	*MAML2*	*MAST1*	*MAST2*	*MEAF6*
	MECOM	*MET*	*MKL1*	*MKL2*	*MSMB*	*MUSK*	*MYB*
	MYC	*NCOA2*	*NOTCH1*	*NOTCH2*	*NRG1*	*NTRK1*	*NTRK2*
	NTRK3	*NUMBL*	*NUP214*	*NUP98*	*NUT*	*PAX5*	*PAX8*
	PDGFB	*PDGFRA*	*PDGFRB*	*PICALM*	*PIK3CA*	*PKN1*	*PLAG1*
	PPARG	*PRKACA*	*PRKCA*	*PRKCB*	*PTK2B*	*RAF1*	*RARA*
	RBM15	*RELA*	*RET*	*ROS1*	*RSPO2*	*RSPO3*	*RUNX1*
	RUNX1T1	*SS18*	*STAT6*	*TAF15*	*TAL1*	*TCF12*	*TCF3*
	TERT	*TFE3*	*TFEB*	*TFG*	*THADA*	*TLX3*	*TMPRSS2*
	TSLP	*TYK2*	*USP6*	*VGLL2*	*YWHAE*		

TERT promoter region is not included.

Supplemental Table S7d: Texas Children's Hospital heme DNA mutation panel, version 2

ABL1	ABL2	AKT1	AKT2	ALK	ANK3	APC	ARAF	ARID1A
ASXL1	ASXL2	ATM	ATRX	BAZ1A	BCL11B	BCOR	BCORL1	BIRC6
BRAF	CALR	CBL	CBLB	CCND1	CCND2	CCND3	CCT6B	CDKN1B
CDKN2A	CDKN2B	CEBPA	CHD4	CNOT3	CREBBP	CRLF2	CSF1R	CSF3R
CTCF	CUX1	DDX3X	DDX41	DHX15	DNM2	DNMT3A	ECT2L	EED
EIF6	ELF1	EP300	EPOR	ERG	ETNK1	ETV6	EZH2	FAT1
FAT4	FBXW7	FLT3	FPGS	GATA1	GATA2	GATA3	GNA13	GNAS
H3F3A	HDAC9	HRAS	HUWE1	ID3	IDH1	IDH2	IKZF1	IKZF2
IKZF3	IL7R	JAK1	JAK2	JAK3	KANSL1	KAT6B	KDM5A	KDM6A
KIT	KMT2A	KMT2C	KMT2D	KRAS	LEF1	MAP2K1	MBNL1	MED12
MGA	MLH1	MLLT3	MPL	MSH2	MSH6	MYB	MYC	MYCN
MYD88	NF1	NIPBL	NOTCH1	NOTCH2	NOTCH3	NPM1	NR3C1	NR3C2
NRAS	NSD2	NT5C2	ORAI1	PAX5	PCBP1	PDGFRA	PDGFRB	PHF6
PHIP	PIK3C2A	PIK3CA	PIK3CD	PIK3R1	PMS2	PPM1D	PRDM2	PRPS1
PRPS2	PTEN	PTPN11	PTPRC	RAD21	RB1	RELN	RHOA	RIT1
RPL10	RPL5	RUNX1	SAMD9	SAMD9L	SETBP1	SETD2	SETX	SF3A1
SF3B1	SH2B3	SMARCA4	SMARCB1	SMC1A	SMC3	SOS1	SRSF2	STAG2
STAT3	STAT5B	SUZ12	SYNE1	TERT	TET1	TET2	TNFRSF14	TP53
TSPYL2	U2AF1	U2AF2	UBTF	USP7	USP9X	VPREB1	WT1	ZBTB7A
ZEB2	ZFHX3	ZRSR2						

Minimum reportable VAF for single nucleotide variants is 5%. TERT promoter region covered with at least 100x read depth, but often >200x, with an additional manual review of the hotspot -124 C>T and -146 C>T variants.

Supplemental Table S7e: Dana Farber/BWH Rapid Heme Panel

ABL1 ENST00000372348 5 alt e1; ABL1 ENST00000318560 5 e1-e10
ASXL1 ENST00000306058 4 e11-e12
ATM ENST00000278616 4 e2-e63
ATRX ENST00000373344 5 e1-e35
BCOR ENST00000378444 4 e2-e15
BCORL1 ENST00000540052 1 e1-e12; BCORL1 NM_001184772 1 alt e8
BRAF ENST00000288602 6 e12-e16
BRCC3 ENST00000369462 1 e1-e11
BTK ENST00000308731 7 e11, e15-e16
CALR ENST00000316448 5 e9
CBL ENST00000264033 4 e7-e9
CCND1 ENST00000227507 2 e1-e5
CD79B ENST00000392795 3 e5-e6
CDKN2A ENST00000498124 1 e1-e4; CDKN2A NM_058195 1 alt e1
CDKN2B ENST00000276925 6 e1-e2
CEBPA ENST00000498907 2 e1
CREBBP ENST00000262367 5 e1-e31
CRLF2 ENST00000381567 3 e5
CSF3R ENST00000373103 1 e14-e17
CSNK1A1 ENST00000377843 2 e1-e10; CSNK1A1 ENST00000515768 2 alt e5
CTCF ENST00000264010 4 e3-e12
CUX1 ENST00000292535 7 e1-e24; CUX1 ENST00000292538 7 alt e1, e15-e23
CXCR4 ENST00000241393 3 e3
DDX41 ENST00000507955 1 e1-e17
DKC1 ENST00000369550 5 e1-e15
DNMT3A ENST00000380746 3 alt e1-e2; DNMT3A ENST00000264709 3 e2-e23; ; DNMT3A NM_001320893 3 alt e1
EP300 ENST00000263253 7 e1-e31
ERG ENST00000288319 2 alt e1; ERG ENST00000417133 2 e3-e12; ERG NM_001243432 2 alt e12
ETNK1 ENST00000266517 4 e3
ETV6 ENST00000396373 4 e1-e8
EZH2 ENST00000320356 2 e2-e20
FBXW7 ENST00000281708 4 e10-e14
FLT3 ENST00000241453 7 e14, e16-e17,e20
GATA1 ENST00000376670 3 e2-e6
GATA2 ENST00000341105 2 e2-e6
GNAS ENST00000371085 3 e8-e9
GNB1 ENST00000378609 4 e5-e6
IDH1 ENST00000345146 2 e3-e10
IDH2 ENST00000330062 3 e1-e11
IKZF1 ENST00000331340 3 e2-e8; IKZF1 ENST00000413698 3 alt e4; IKZF1 ENST00000492782 3 alt e5
IL7R ENST00000303115 3 e5-e7
JAK1 ENST00000342505 4 e10-e25
JAK2 ENST00000381652 3 e12-e20
JAK3 ENST00000458235 1 e16-e24
KIT ENST00000288135 5 e8-e11, e17
KRAS ENST00000311936 3 e2-e6
KRAS ENST00000256078 3 alt e5 3
KMT2A ENST00000534358 1 e1-e13, e24-e26
MAP2K1 ENST00000307102 5 e2-e3, e6 e28-e30
MPL ENST00000372470 3 e4, e10
NF1 ENST00000356175 3 e1-e57; NF1 ENST00000358273 3 alt e31
MYC ENST00000377970 2 e1-e3
MYD88 ENST00000396334 3 e5
NOTCH2 ENST00000256646 2 e24-e28, e34
NFE2 ENST00000553070 1 e3-e4
NOTCH1 ENST00000277541 6 e24-e28, e34
NSD2 ENST00000382895 3 e20-e21
NPM1 ENST00000296930 5 e10-e11
NRAS ENST00000369535 4 e2-e5
PIGA ENST00000333590 4 e1-e62
NT5C2 ENST00000343289 5 e10-e18
PHF6 ENST00000394292 1 e1-e9
PRPF8 ENST00000304992 6 e25-e34
PLCG2 ENST00000359376 3 e18-e19, e23 e26, e29
PPM1D ENST00000305921 3 e6
RAD21 ENST00000297338 2 e2-e14
PTEN ENST00000371953 3 e1-e9
PTPN11 ENST00000351677 2 e1-e15
SBDS ENST00000246868 2 e1-e5
RIT1 ENST00000368322 3 alt e1; RIT1 ENST00000368323 3 e2-e6
RUNX1 ENST00000437180 1 e2-e9; RUNX1 ENST00000358356 1 alt e5
SF3B1 ENST00000335508 6 e12-e18
SETBP1 ENST00000282030 5 e4
SETD2 ENST00000409792 3 e1-e21
SMC3 ENST00000361804 4 e1-e29
SH2B3 ENST00000538307 2 alt e1; SH2B3 ENST00000341259 2 e2-e8
SMC1A ENST00000322213 4 e1-e25; SMC1A NM_001281463 4 alt e2
STAT3 ENST00000264657 5 e2-e24
SRSF2 ENST00000359995 5 e1
STAG2 ENST00000218089 9 e3-e35
TERT ENST00000310581 5 e1-e16
STAT5B ENST00000293328 3 e13-e19
TERC ENST00000602385 1 e1
U2AF1 ENST00000291552 4 e2,e6
TET2 ENST00000380013 4 e3-e11
TP53 ENST00000269305 4 e2-e11; TP53 ENST00000420246 4 alt e10
ZRSR2 ENST00000307771 7 e1-e11
WT1 ENST00000379079 3 alt e1; WT1 ENST00000332351 3 e1-e10
XPO1 ENST00000401558 2 e15-e16

Sensitivity of detection of >2% VAF, but in certain regions may allow for detection of variants down to 1%. For serial analyses, known variants detected in previous studies were examined manually, allowing detected on <1% variants. TERT promoter region is not covered.

Supplemental Table S7f: The Ohio State James Comprehensive Hematology Genomic Panel

Comprehensive Hematology Panel (CHP) Gene List (core germline assessment genes in AML/MDS boxed)															
ABCB1	BCL10	CCR4	CREB1	**ELANE**	FANCG	GLI1	IGF2	KLF2	MLH3	NTRK1	POLD1	RICTOR	**SBDS**	SPOP	TOP1
ABL1	BCL11A	CD19	CREBBP	EP300	FANCI	GNA11	IKBKB	KLF4	MLLT10	NTRK2	POLE	RIF1	SDHA	SRC	TOP2A
**ACD**	BCL11B	CD27	CRKL	EPC1	FANCL	GNA13	IKBKE	KLHL14	MPL	NTRK3	**POT1**	RIPK1	SDHAF2	**SRP72**	**TP53**
ADAMTS1	BCL2	CD28	CRLF2	EPCAM	FANCM	GNAQ	IKZF1	KLHL6	MRE11A	NUP98	PPARG	RIT1	SDHB	SRSF2	TRAF2
AFF2	BCL2A1	CD36	CSF1R	EPHA2	FAS	GNAS	IKZF2	KMT2A	MSH2	NUTM1	PPM1D	RMRP	SDHC	SS18	TRAF3
AICDA	BCL2L1	CD3EAP	CSF3R	EPHA3	FAT3	GNB1	IKZF3	KMT2B	MSH6	P2RY8	PPP2R1	RNF43	SDHD	STAG2	TRAF7
AKT1	BCL6	CD40LG	CSMD1	EPHA5	FBXO11	GPC3	IL21R	KMT2C	MTOR	PALB2	PRDM1	ROBO1	SEC23B	STAT1	TSC1
AKT2	BCL7A	CD58	**CTC1**	EPHA7	FBXW7	GRIN2A	IL7R	KMT2D	MUTYH	PARK2	PRF1	ROBO2	SEPT2	STAT3	TSC2
AKT3	BCOR	CD70	CTCF	EPHB1	FGF19	GSK3B	INO80	KRAS	MXRA5	**PARN**	PRKAR1	ROS1	SETBP1	STAT5A	TSHR
ALAS2	BCORL1	CD74	CTLA4	EPHB2	FGF23	H3F3A	INPP4B	LIG4	MYC	PAX5	PRKCB	RPL5	SETD2	STAT5B	TYK2
ALK	BIRC3	CD79A	CTNNA1	EPHB4	FGF3	**HAX1**	INPP5D	LMO1	MYCL1	PBRM1	PRKD2	RPL10	SETDB1	STAT6	U2AF1
AMER1	BLM	CD79B	CTNNB1	EPO	FGF4	HGF	IRF1	LRP1B	MYCN	PCBP1	PRKDC	RPL11	SF1	STK11	U2AF2
**ANKRD26**	BMPR1A	CD274	CUX1	ERBB2	FGFR1	HIST1H1C	IRF2BP	LUC7L2	MYD88	PCDHG	PRPF40	RPL15	SF3A1	STX11	UBE2T
APC	BRAF	CD276	CXCR4	ERBB3	FGFR2	HIST1H1D	IRF4	LYST	MYH11	PDCD1	PTCH1	RPL26	SF3B1	STXBP2	UBR5
AR	**BRCA1**	CDAN1	CYLD	ERBB4	FGFR3	HIST1H1E	IRF8	MAGED1	MYH9	PDCD1L	PTEN	RPL27	SGK1	SUFU	UNC13D
ARAF	**BRCA2**	CDC73	DAXX	ERCC1	FGFR4	HIST1H2AC	IRS2	MAGT1	MYSM1	PDGFRA	PTPN11	RPL31	SH2B3	SUZ12	USB1
ARHGAP2	BRCC3	CDH1	DDR2	ERCC2	FH	HIST1H2A	ITGA9	MALT1	**NAF1**	PDGFRB	PTPRC	RPL35A	SH2D1A	SYK	VEGFA
ARID1A	BRIP1	CDH2	DDX3X	ERCC3	FLCN	HIST1H2BC	ITGAM	MAP2K1	NBN	PHF6	RAB35	RPN1	SHOC2	TAL1	VHL
ARID1B	BTG1	CDK12	**DDX41**	ERCC4	FLI1	HIST1H2BK	ITGAV	MAP2K2	NCOR1	PHIP	RAC1	RPS7	SLIT2	TAZ	VPS13B
ARID2	BTG2	CDK4	DICER1	ERCC5	FLT1	HIST1H2B	ITK	MAP2K4	NF1	PHOX2	RAC2	RPS10	SLX4	TBL1XR1	VPS45
ARID3A	BTK	CDK6	DIS3	ERG	FLT3	HIST1H3B	ITPKB	MAP3K1	NF2	PICALM	RAD21	RPS15	SMAD2	TCEB1	WAS
ARID5B	BUB1B	CDK8	**DKC1**	ESR1	FLT4	HIST1H3C	JAK1	MAP3K1	NFE2L2	PIGA	RAD50	RPS17L	SMAD3	TCF3	WHSC1
ASMTL	C15ORF65	CDKN1A	DLL1	ETV1	FOXA1	HNF1A	JAK2	MAPK1	NFKB2	PIK3CA	RAD51	RPS19	SMAD4	TCF7L2	WIPF1
ASXL1	CALR	CDKN1B	DNAH5	ETV5	FOXL2	HOXA11	JAK3	MCL1	NFKBIA	PIK3CB	RAD51C	RPS24	SMARCA2	**TERC**	WISP3
ASXL2	CARD11	CDKN2A	DNM2	**ETV6**	FOXO1	HOXA9	JAZF1	MDM2	**NHP2**	PIK3CD	RAD51D	RPS26	SMARCA4	**TERT**	**WRAP53**
ATM	CASP8	CDKN2B	DNMT3A	EWSR1	FOXO3	HRAS	JUN	MDM4	NKX2-1	PIK3CG	RAF1	RPS27	SMARCB1	TET2	WRN
ATR	CASP10	CDKN2C	DOCK2	EZH2	FOXP1	HUWE1	KAT6A	MECOM	**NOP10**	PIK3R1	RARA	RPS29	SMC1A	TET3	WT1
ATRX	CBFB	**CEBPA**	DOCK8	FAM46	FUBP1	ID1	KDM5A	MED12	NOTCH1	PIK3R2	RB1	RPTOR	SMC3	TFE3	XIRP2
AURKA	CBL	CHD2	DOT1L	FAM5C	FUS	ID2	KDM5C	MEF2B	NOTCH2	PIM1	RBBP6	RRAGC	SMO	TGFBR2	XPO1
AURKB	CBLB	CHEK1	DTX1	FANCA	FYN	ID3	KDM6A	MEN1	NOTCH3	PLAG1	RBM8A	**RTEL1**	SOCS1	THPO	XRCC2
AXIN1	CBLC	CHEK2	DUSP2	FANCB	G6PC3	IDH1	KDR	MET	NOTCH4	PLCG1	RBM10	**RUNX1**	SOCS2	**TINF2**	ZAP70
AXL	CCND1	CHIC2	EBF1	FANCC	GATA1	IDH2	KEAP1	MIR142	NPM1	PLCG2	REL	RUNX1T1	SOCS3	TLL2	ZFHX4
B2M	CCND2	CIC	EFL1	FANCD2	**GATA2**	IFNGR1	KIF23	MITF	NRAS	PLXNA1	RELA	S1PR2	SOS1	TLR4	ZMYM3
BAP1	CCND3	CIITA	EGFR	FANCE	GATA3	IFNGR2	KIT	MKL1	NSD1	PML	RET	**SAMD9**	SOX2	TMPRSS2	ZNF217
BARD1	CCNE1	CNOT3	EGR2	FANCF	GFI1	IGF1R	KLF1	MLH1	NT5C2	PMS1	RHEB	**SAMD9L**	SOX9	TNFAIP3	ZRSR2
										PMS2	RHOA	SAMHD1	SPEN	TNFRSF14	

The panel includes TERT promoter region, with targeted read depth for all genes of at least 200X.

Supplemental Table S7g: Washington University error corrected NGS panel

ASXL1	*FLT3*	*RUNX1*	*ZNF318*
ATM	*GNAS*	*SETBP1*	*ZRSR2*
ATRX	*GNB1*	*SETD2*	
BCOR	*HBB*	*SF1*	
BCORL1	*IDH1*	*SF3B1*	
BRCC3	*IDH2*	*SH2B3*	
BUB1B	*IL17RA*	*SMARCD2*	
CBL	*JAK2*	*SMC1A*	
CEBPA	*KDM6A*	*SMC3*	
CEBPB	*KRAS*	*SRCAP*	
CREBBP	*LUC7L2*	*SRSF2*	
CSF3R	*MYD88*	*STAG2*	
CUX1	*NPM1*	*STAT3*	
CXCL12	*NRAS*	*TET2*	
CXCR2	*PHF6*	*TFR2*	
CXCR4	*PKLR*	*TP53*	
DNMT3A	*PPM1D*	*TUBB1*	
EPO	*PTPN11*	*U2AF1*	
EZH2	*RAD21*	*ZBTB33*	

Error corrected sequencing, with sensitivity of detection of variants down to VAF of >0.1%. TERT promoter region was not covered.

Supplemental Table S8: Published 10X Genomics single cell RNA datasets of unaffected controls

Control Name	Cell Type	Citation
10k PBMCs Healthy Donor v3 Chemistry	PBMC	10x Genomics (https://www.10xgenomics.com/resources/datasets/10-k-pbm-cs-from-a-healthy-donor-v-3-chemistry-3-standard-3-0-0)
Frozen PBMCs Donor A	PBMC	Zheng, G., Terry, J., Belgrader, P. et al. Massively parallel digital transcriptional profiling of single cells. Nat Commun 8, 14049 (2017). https://doi.org/10.1038/ncomms14049
Frozen PBMCs Donor B	PBMC	Zheng, G., Terry, J., Belgrader, P. et al. Massively parallel digital transcriptional profiling of single cells. Nat Commun 8, 14049 (2017). https://doi.org/10.1038/ncomms14049
Frozen PBMCs Donor C	PBMC	Zheng, G., Terry, J., Belgrader, P. et al. Massively parallel digital transcriptional profiling of single cells. Nat Commun 8, 14049 (2017). https://doi.org/10.1038/ncomms14049
Frozen_BMMCs_Healthy_Control_1	BMMC	Zheng, G., Terry, J., Belgrader, P. et al. Massively parallel digital transcriptional profiling of single cells. Nat Commun 8, 14049 (2017). https://doi.org/10.1038/ncomms14049
Frozen_BMMCs_Healthy_Control_2	BMMC	Zheng, G., Terry, J., Belgrader, P. et al. Massively parallel digital transcriptional profiling of single cells. Nat Commun 8, 14049 (2017). https://doi.org/10.1038/ncomms14049

Supplemental Table S9: Skin fibroblast cell lines used in cell growth experiments

Study ID	Diagnosis	Germline variant
257.01	TBD	*DKC1* c.29C>T p.Pro10Leu
328.01	TBD	*DKC1* c.838A>C p.Ser280Arg
398.01	TBD	*DKC1* c.-35G>A
373.01	TBD	*TERC* n.173A>G
373.04	TBD	*TERC* n.173A>G
629.4	TBD	*TERC* r.314T>A
43.01	Control (acquired BMF, PNH)	n/a
58.01	Control (acquired BMF, PNH)	n/a
370.01	Control (acquired BMF, PNH)	n/a
489.01	Control (acquired BMF, PNH)	n/a

Supplemental Table S10: Antibodies used for western blotting in ATM inhibition experiments

Antibody	Dilution	Clone	Catalog Number	Company
anti-ATM	1:1000	D2E2	2873	Cell Signaling Technology, Danvers, MA
anti-pATM (S1981)	1:1000	EP1890Y	ab81292	Abcam, Waltham, MA
anti-Chk2	1:250	19/Chk2	611570	BD Biosciences, San Jose, CA
anti-pChk2 (T68)	1:1000	C13C1	2197	Cell Signaling Technology, Danvers, MA
anti-KAP1	1:1000	polyclonal	A300-274A	Bethyl Laboratories, Montgomery, TX
anti-pKAP1 (S824)	1:1000	BL-246-7B5	A700-013	Bethyl Laboratories, Montgomery, TX
anti-GAPDH	1:1000	14C10	2118	Cell Signaling Technology, Danvers, MA
anti-Vinculin	1:2000	E1E9V	13901	Cell Signaling Technology, Danvers, MA
anti-mouse IgG, HRP-linked	1:5000	n/a	7076	Cell Signaling Technology, Danvers, MA
anti-rabbit IgG, HRP-linked	1:5000	n/a	7074	Cell Signaling Technology, Danvers, MA

Supplemental Script 1: Fiji macro to analyze nuclear 53BP1 and telomere foci

Instructions for thresholding script:
1. Load up the original image in Fiji
2. Upload the script via Plug-in —> Edit
3. Adjust the name of the image for the one that is selected
4. Ensure saving pathways are accurate (i.e. the folder the data is being saved in).
5. Output should include saved Tiff images of thresholded, masked images along with the number of dots/selected regions of interest within the nuclei.

```
#select nuclei channel
selectImage("DAPI_CHANNEL_IMAGE");
run("Duplicate...", "title=nuclei");
run("Grays");
run("Median...", "radius=6");
setAutoThreshold("Otsu dark");
//run("Threshold...");
setThreshold(10, 255, "raw");
//setThreshold(10, 255);
setOption("BlackBackground", true);
run("Convert to Mask");
run("Fill Holes");
run("Open");


run("Set Measurements...", "area mean min bounding integrated limit display redirect=None decimal=2");
selectImage("nuclei");
run("Analyze Particles...", "size=10-Infinity exclude summarize add");
Table.rename("Summary", "nuclei");
saveAs("Results", "/ENTER_FILE_PATH_HERE/nuclei.csv");


#select 53bp1 channel
selectImage("53BP1_CHANNEL_IMAGE");
run("Duplicate...", "title=53bp1");
run("Grays");
run("Median...", "radius=2");
setAutoThreshold("Percentile dark");
//run("Threshold...");
//setThreshold(89, 255);
setThreshold(89, 255, "raw");
run("Convert to Mask");
run("Open");
run("Watershed");
run("Set Measurements...", "area mean min bounding integrated limit display redirect=None decimal=2");


selectImage("53bp1");
n = roiManager("count");

             for (i = 0; i < n; i++) {
```

```
            roiManager("Select", i);
            run("Analyze Particles...", "size=0-Infinity exclude summarize add");
            roiManager("Show All without labels");
            }

Table.rename("Summary", "53bp1");
saveAs("Results", "/ENTER_FILE_PATH_HERE/53bp1.csv");
roiManager("Select", 1);
run("Select All");
roiManager("Save", "/ENTER_FILE_PATH_HERE/RoiSet_nuclei_53bp1.zip");
selectImage("53bp1");
saveAs("Tiff", "/ENTER_FILE_PATH_HERE/53bp1.tif");
selectImage("nuclei");
saveAs("Tiff", "/ENTER_FILE_PATH_HERE/nuclei.tif");

#select telomere channel
selectImage("TELOMERE_CHANNEL_IMAGE ");
run("Duplicate...", "title=telomere");
selectImage("telomere");
run("Grays");
run("Median...", "radius=0.5");
setAutoThreshold("Default dark");
//run("Threshold...");
setThreshold(50, 255, "raw");
//setThreshold(50, 255);
setOption("BlackBackground", true);
run("Convert to Mask");
run("Open");
run("Watershed");

selectImage("telomere");
n = roiManager("count");

            for (i = 0; i < n; i++) {
            roiManager("Select", i);
            run("Analyze Particles...", "size=0-Infinity exclude summarize add");
            roiManager("Show All without labels");
            }

Table.rename("Summary", "telomere");
saveAs("Results", "/ENTER_FILE_PATH_HERE/telomere.csv");
roiManager("Select", 1);
run("Select All");
roiManager("Save", "/ENTER_FILE_PATH_HERE/RoiSet_nuclei_telomere.zip");
selectImage("telomere");
saveAs("Tiff", "/ENTER_FILE_PATH_HERE/telomere.tif");
```

Supplemental Script 2: Fiji macro to analyze β-galactosidase staining

Supplemental Script 2a: Fiji macro to count the number of nuclei based on DAPI-stained image.

```
// Fiji (ImageJ2) Macro
// This macro processes TIFF files in a specified folder:
// 1. Opens each TIFF image in the folder.
// 2. Applies a preset threshold (100-255) and then allows manual adjustments.
// 3. Analyzes particles (size: 50-Infinity, no circularity constraint, include holes).
// 4. Saves ROI measurements and the total number of cells per image into an Excel file.

// Set the input folder
inputFolder = getDirectory("Choose a folder containing TIFF images");

// Start processing the folder
fileList = getFileList(inputFolder);

for (i = 0; i < fileList.length; i++) {
    if (endsWith(fileList[i], ".tif") || endsWith(fileList[i], ".tiff")) {
        // Open the image
        open(inputFolder + fileList[i]);

        // Apply preset threshold and allow manual adjustments
        setThreshold(100, 255);
        waitForUser("Adjust the threshold if needed, then click OK to continue.");
        run("Convert to Mask");

        // Analyze particles
        run("Analyze Particles...", "size=50-Infinity add include");

        // Get the total number of cells
        totalCells = roiManager("count");

        // Measure ROIs
        run("Set Measurements...", "area mean standard centroid perimeter bounding redirect=None decimal=5");
        roiManager("Measure");

      // Add total cell count to the Results table
        setResult("Number_of_cells", 0, totalCells);

        // Save the results to Excel
        path = inputFolder + fileList[i] + "_Measurements.xlsx";
        run("Read and Write Excel", "file=[" + path + "]");

       // Clear previous measurements
        run("Clear Results");

        // Close the image and clear results
        close();
        roiManager("reset");
    }
}
```

```
print("Analysis completed. ROI measurements and cell counts saved for each file.");
```

Supplemental Script 2b: Fiji macro to convert β-galactosidase-stained color tiff image into binary mask based on pre-specified Hue, Saturation and Brightness thresholds.

```
// Fiji (ImageJ2) Macro
// Processes TIFF files and creates binary masks based on Hue, Saturation, and Brightness thresholds.

// Function to perform color thresholding and create binary mask
function applyColorThreshold() {
    min = newArray(3);
    max = newArray(3);
    run("RGB Color"); // Ensure image is in RGB format before HSB Stack
    run("HSB Stack");
    run("Stack to Images");

    // Process Hue channel
    selectImage(1); // First slice is Hue
    min[0] = 127; max[0] = 148;
    setThreshold(min[0], max[0]);
    run("Convert to Mask");
    rename("TempHueMask");

    // Process Saturation channel
    selectImage(2); // Second slice is Saturation
    min[1] = 66; max[1] = 230;
    setThreshold(min[1], max[1]);
    run("Convert to Mask");
    rename("TempSatMask");

    // Process Brightness channel
    selectImage(3); // Third slice is Brightness
    min[2] = 0; max[2] = 227;
    setThreshold(min[2], max[2]);
    run("Convert to Mask");
    rename("TempBrightMask");

    // Combine masks (Hue, Saturation, Brightness)
    imageCalculator("AND create", "TempHueMask", "TempSatMask");
    rename("TempHS_Mask");
    imageCalculator("AND create", "TempHS_Mask", "TempBrightMask");
    rename("BinaryImage");

    // Close intermediate windows
    selectWindow("TempHueMask"); close();
    selectWindow("TempSatMask"); close();
    selectWindow("TempBrightMask"); close();
    selectWindow("TempHS_Mask"); close();
}

// Set the input and output folders
inputFolder = getDirectory("Choose a folder containing TIFF images");
outputFolder = getDirectory("Choose a folder to save binary masks");
```

```
fileList = getFileList(inputFolder);

for (i = 0; i < fileList.length; i++) {
    if (endsWith(fileList[i], ".tif") || endsWith(fileList[i], ".tiff")) {
        // Open the image
        open(inputFolder + fileList[i]);

        // Apply color thresholding and create binary mask
        applyColorThreshold();

        // Save the binary mask to the output folder
        binaryMaskPath = replace(outputFolder + fileList[i] + "_BinaryMask.tif", "/", "\\");
        saveAs("Tiff", binaryMaskPath);

        // Close the image
        close();
    }
}

print("Binary mask creation completed. Masks saved to output folder.");
```

Supplemental Script 2c: Fiji macro to quantify staining based on converted binary mask files of color threshold-adjusted β-galactosidase-stained images.

```
// Fiji (ImageJ2) Macro
// Processes binary images, applies threshold 255, 255, analyzes particles, and saves results individually.

// Set the input and output folders
inputFolder = getDirectory("Choose a folder containing binary images");
outputFolder = getDirectory("Choose a folder to save analysis results");
fileList = getFileList(inputFolder);

for (i = 0; i < fileList.length; i++) {
    if (endsWith(fileList[i], ".tif") || endsWith(fileList[i], ".tiff")) {
        // Open the binary image
        open(inputFolder + fileList[i]);

        // Apply Threshold on Binary Image
        setThreshold(255, 255);
        run("Convert to Mask");

        // Analyze particles over 50 pixels, include holes, and use pixels as units
        run("Set Measurements...", "area mean standard centroid perimeter bounding integrated median redirect=None decimal=5");
        run("Analyze Particles...", "size=50-Infinity include holes display include pixel");



        // Measure and save results
        roiManager("Measure");
        resultPath = replace(outputFolder + fileList[i] + "_Results.csv", "/", "\\");
        saveAs("Results", resultPath);

        // Clear results and reset for the next image
```

```
        run("Clear Results");
        roiManager("reset"); // Reset ROI Manager
        close(); // Close current image
    }
}

// Print completion message
print("Analysis completed. Results saved to output folder.");
```

Supplemental references

1. Revy P, Kannengiesser C, and Bertuch AA. Genetics of human telomere biology disorders. *Nat Rev Genet.* 2023;24(2):86-108.
2. Savage SA, and Niewisch MR. In: Adam MP, Feldman J, Mirzaa GM, Pagon RA, Wallace SE, Bean LJH, Gripp KW, and Amemiya A eds. *GeneReviews((R)).* Seattle (WA); 1993.
3. McKenna A, Hanna M, Banks E, Sivachenko A, Cibulskis K, Kernytsky A, Garimella K, Altshuler D, Gabriel S, Daly M, et al. The Genome Analysis Toolkit: a MapReduce framework for analyzing next-generation DNA sequencing data. *Genome Res.* 2010;20(9):1297-303.
4. Chakravarty D, Gao J, Phillips SM, Kundra R, Zhang H, Wang J, Rudolph JE, Yaeger R, Soumerai T, Nissan MH, et al. OncoKB: A Precision Oncology Knowledge Base. *JCO Precis Oncol.* 2017;2017(
5. Perdigones N, Perin JC, Schiano I, Nicholas P, Biegel JA, Mason PJ, Babushok DV, and Bessler M. Clonal hematopoiesis in patients with dyskeratosis congenita. *Am J Hematol.* 2016;91(12):1227-33.
6. Amemiya HM, Kundaje A, and Boyle AP. The ENCODE Blacklist: Identification of Problematic Regions of the Genome. *Sci Rep.* 2019;9(1):9354.
7. Aubert G, Baerlocher GM, Vulto I, Poon SS, and Lansdorp PM. Collapse of telomere homeostasis in hematopoietic cells caused by heterozygous mutations in telomerase genes. *PLoS Genet.* 2012;8(5):e1002696.
8. Alder JK, Hanumanthu VS, Strong MA, DeZern AE, Stanley SE, Takemoto CM, Danilova L, Applegate CD, Bolton SG, Mohr DW, et al. Diagnostic utility of telomere length testing in a hospital-based setting. *Proc Natl Acad Sci U S A.* 2018;115(10):E2358-E65.
9. Young MD, and Behjati S. SoupX removes ambient RNA contamination from droplet-based single-cell RNA sequencing data. *Gigascience.* 2020;9(12).
10. McGinnis CS, Murrow LM, and Gartner ZJ. DoubletFinder: Doublet Detection in Single-Cell RNA Sequencing Data Using Artificial Nearest Neighbors. *Cell Syst.* 2019;8(4):329-37 e4.
11. Zheng GX, Terry JM, Belgrader P, Ryvkin P, Bent ZW, Wilson R, Ziraldo SB, Wheeler TD, McDermott GP, Zhu J, et al. Massively parallel digital transcriptional profiling of single cells. *Nat Commun.* 2017;8(14049.
12. Korsunsky I, Millard N, Fan J, Slowikowski K, Zhang F, Wei K, Baglaenko Y, Brenner M, Loh PR, and Raychaudhuri S. Fast, sensitive and accurate integration of single-cell data with Harmony. *Nat Methods.* 2019;16(12):1289-96.
13. Hao Y, Hao S, Andersen-Nissen E, Mauck WM, 3rd, Zheng S, Butler A, Lee MJ, Wilk AJ, Darby C, Zager M, et al. Integrated analysis of multimodal single-cell data. *Cell.* 2021;184(13):3573-87 e29.
14. Hu BC. The human body at cellular resolution: the NIH Human Biomolecular Atlas Program. *Nature.* 2019;574(7777):187-92.
15. Love MI, Huber W, and Anders S. Moderated estimation of fold change and dispersion for RNA-seq data with DESeq2. *Genome Biol.* 2014;15(12):550.
16. Korotkevich G, Sukhov V, Budin N, Shpak B, Artyomov MN, and Sergushichev A. Fast gene set enrichment analysis. *bioRxiv.* 2021:060012.
17. Liberzon A, Subramanian A, Pinchback R, Thorvaldsdottir H, Tamayo P, and Mesirov JP. Molecular signatures database (MSigDB) 3.0. *Bioinformatics.* 2011;27(12):1739-40.
18. Liberzon A, Birger C, Thorvaldsdottir H, Ghandi M, Mesirov JP, and Tamayo P. The Molecular Signatures Database (MSigDB) hallmark gene set collection. *Cell Syst.* 2015;1(6):417-25.
19. Subramanian A, Tamayo P, Mootha VK, Mukherjee S, Ebert BL, Gillette MA, Paulovich A, Pomeroy SL, Golub TR, Lander ES, et al. Gene set enrichment analysis: a knowledge-based approach for interpreting genome-wide expression profiles. *Proc Natl Acad Sci U S A.* 2005;102(43):15545-50.
20. Robinson TM, Bowman RL, Persaud S, Liu Y, Neigenfind R, Gao Q, Zhang J, Sun X, Miles LA, Cai SF, et al. Single-cell genotypic and phenotypic analysis of measurable residual disease in acute myeloid leukemia. *Sci Adv.* 2023;9(38):eadg0488.
21. Yang TB, Chen Q, Deng JT, Jagannathan G, Tobias JW, Schultz DC, Wang S, Lengner CJ, Rustgi AK, Lynch JP, et al. Mutual reinforcement between telomere capping and canonical Wnt signalling in the intestinal stem cell niche. *Nat Commun.* 2017;8(14766.

22. Wang H, Chen Q, Lee SH, Choi Y, Johnson FB, and Pignolo RJ. Impairment of osteoblast differentiation due to proliferation-independent telomere dysfunction in mouse models of accelerated aging. *Aging Cell.* 2012;11(4):704-13.
23. Schindelin J, Arganda-Carreras I, Frise E, Kaynig V, Longair M, Pietzsch T, Preibisch S, Rueden C, Saalfeld S, Schmid B, et al. Fiji: an open-source platform for biological-image analysis. *Nat Methods.* 2012;9(7):676-82.
