# Supplementary material for: ATM-dependent DNA damage response constrains cell growth and drives clonal hematopoiesis in telomere biology disorders
Source: J Clin Invest. 2025 Apr 3;135(8):e181659. doi: 10.1172/JCI181659 (PMC11996883; doi:10.1172/JCI181659)
Supplement: Unedited blot and gel images [file jci-135-181659-s296.pdf]

Full unedited blots for Figure 8A, the top section (pATM, ATM, and vinculin blots)

pATM, ATM blots

Gel 2

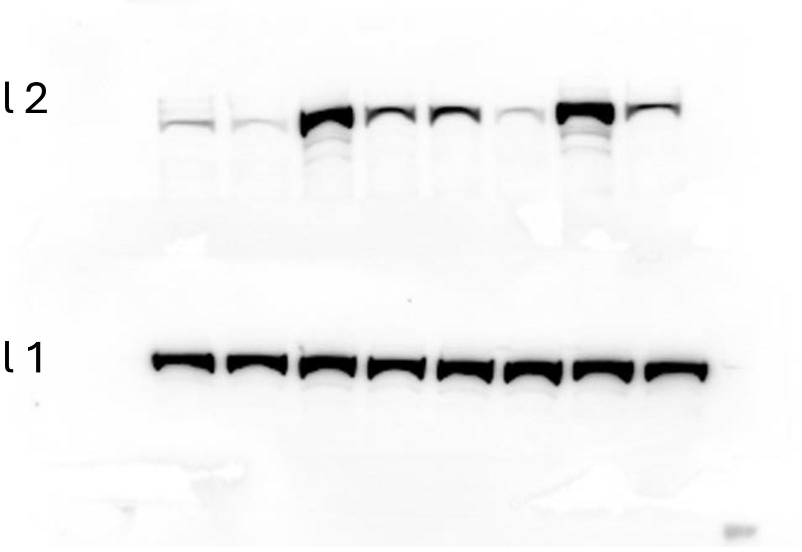

Rabbit anti-pATM, 1:500

Gel 1

Rabbit anti-total ATM, 1:500

Vinculin loading control blots

Gel 2

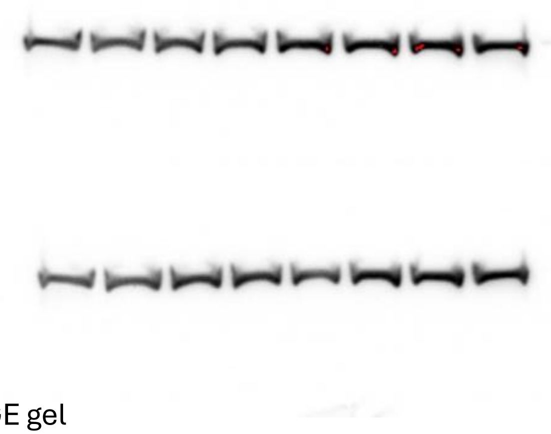

Rabbit anti-vinculin, 1:2000

Gel 1

Rabbit anti-vinculin, 1:2000

7% SDS-PAGE gel

Notes:
The same samples were run on 2 gels, one of which was used to probe pATM (gel 1), and the other—total ATM (gel 2).

Each gel was probed for its own loading control (vinculin), which was used for band intensity normalization for summary statistics.

Vinculin for gel1 was used in the figure.

Full unedited blots for Figure 8A, the bottom section (pKAP1, KAP1, pChk2, Chk2, GAPDH blots)

pKAP1, KAP1 blots

Gel 3

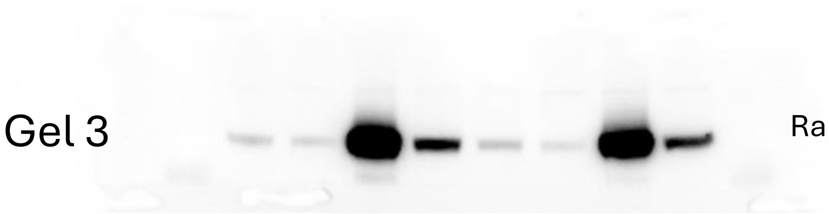

Rabbit anti-pKAP1, 1:2000

Gel 4

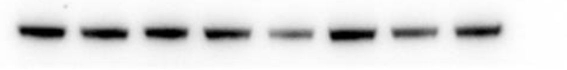

Rabbit anti-KAP1, 1:2000

GAPDH loading control blots

Gel 3

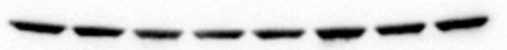

Mouse anti-GAPDH, 1:5000

Gel 4

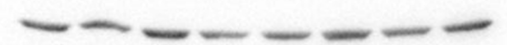

Mouse anti-GAPDH, 1:5000

4-12% Tris-Bis gels

pChk2, Chk2 blots

Gel 3

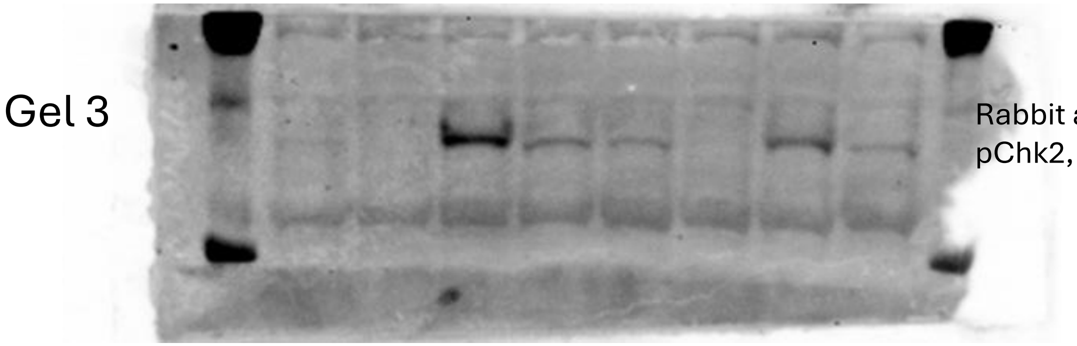

Rabbit anti-pChk2, 1:500

Gel 4

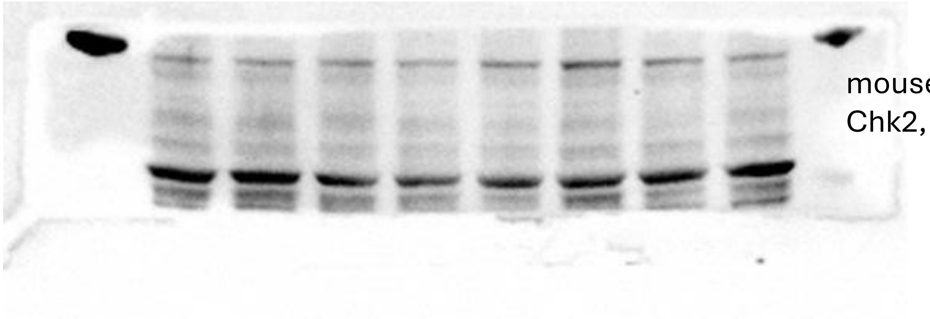

mouse anti-Chk2, 1:500

Notes:

The same samples were run on 2 gels as indicated. Each gel was probed for its own loading control (GAPDH), which was used for band intensity normalization for summary statistics.

GAPDH from gel 3 was used for the figure.

Full unedited blots for Figure 9 G

Total ATM and vinculin (gel 1)

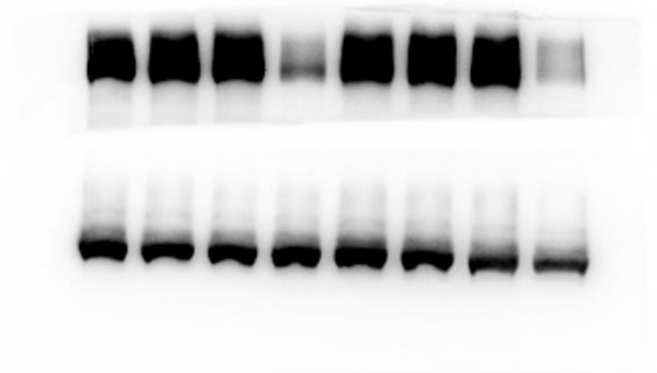

Rabbit anti-ATM, 1:700

Rabbit anti-vinculin, 1:3000

pATM and vinculin (gel 2)

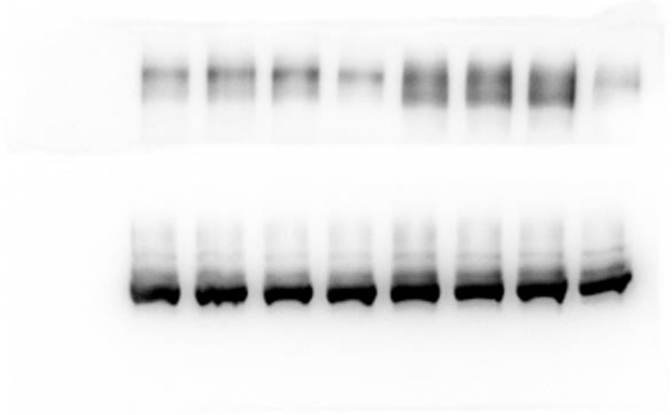

Rabbit anti-pATM, 1:700

Rabbit anti-vinculin, 1:3000

Secondary antibody: goat anti-rabbit 1:5000
7% SDS-PAGE gel

Notes:
The same samples were run on 2 gels as indicated. Each gel had its own loading control immunoblot (vinculin). Vinculin blot from gel 2 was used for the figure.
